# Multi-state catch bond formed in the Izumo1:Juno complex that initiates human fertilization

**DOI:** 10.1038/s41467-025-62427-0

**Published:** 2025-08-26

**Authors:** Sean Boult, Paulina Pacak, Byeongseon Yang, Haipei Liu, Viola Vogel, Michael A. Nash

**Affiliations:** 1 Department of Biosystems Science and Engineering, ETH Zürich, Basel, Switzerland; 2 Institute of Physical Chemistry, Department of Chemistry, University of Basel, Basel, Switzerland; 3 Department of Health Sciences and Technology, ETH Zürich, Zürich, Switzerland; 4 National Center for Competence in Research (NCCR), Molecular Systems Engineering, Basel, Switzerland; 5 Swiss Nanoscience Institute, Basel, Switzerland

**Keywords:** Single-molecule biophysics, Proteins, Atomic force microscopy, Computational biophysics

## Abstract

Izumo1:Juno-mediated adhesion between sperm and egg cells is essential for mammalian sexual reproduction. However, conventional biophysical and structural approaches have provided only limited functional insights. Using atomic force microscopy-based single-molecule force spectroscopy and all-atom steered molecular dynamic simulations, we explore the role of mechanical forces in regulating the human Izumo1:Juno complex. Our findings reveal a multi-state catch bond capable of withstanding forces up to 600 pN– mechanostability rarely observed among eukaryotic protein complexes. We find that this enhanced mechanostability is impaired in the infertility-associated mutant, JunoH177Q. Detailed steered molecular dynamics simulations show how force-dependent structural reorganization of the Izumo1:Juno complex engages previously undiscovered binding conformations to achieve this state of high mechanostability. Overall, this study significantly enhances our understanding of the mechanical underpinnings that regulate human fertilization.

## Abstract (web summary)

Sperm–egg adhesion is crucial for mammalian reproduction. Here, authors report the human Izumo1:Juno complex, a key regulator of sperm-egg adhesion, forms an unusually strong bond through a secondary binding site, which is impaired in an infertility-associated Juno mutant.

## Introduction

**Fig. 1:**
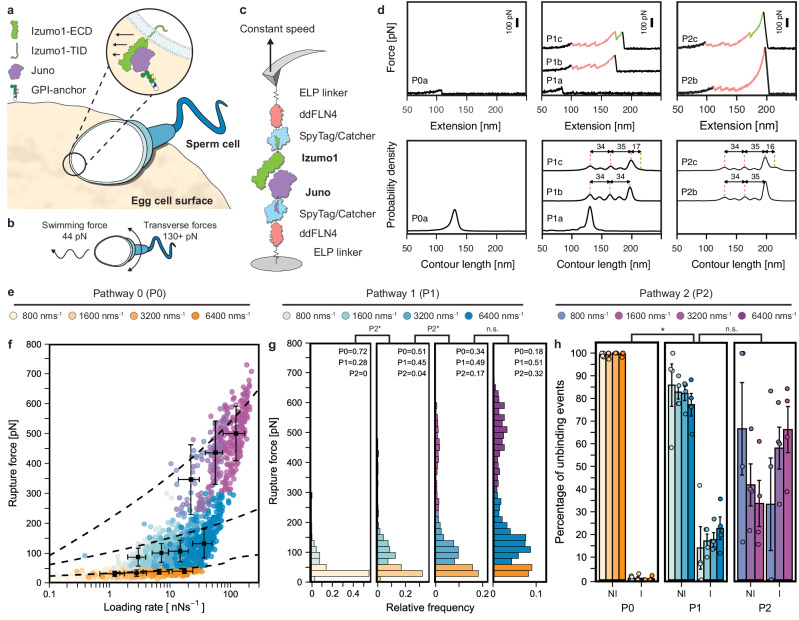
Constant speed AFM-SMFS of Izumo1:Juno reveals catch bonding and multiple unbinding pathways. **a** Sperm:egg adhesion through the Izumo1:Juno complex. **b** Forward swimming and transverse motive forces for healthy sperm. **c** Schematic showing protein constructs and surface immobilization for constant speed AFM-SMFS. **d** Above: representative P0, P1, and P2 force *vs*. extension curves and their subtypes (**a**–**c**). Bottom: combined contour length histograms prepared using cross-correlation analysis to align contour length distributions pooled from four independent experiments for P0a (*n* = 116), P1a (*n* = 22), P1b (*n* = 243), P1c (*n* = 62), P2b (*n* = 66), and P2c (*n* = 142) at 6400 nm·s^−1^. **e** Legend for P0, P1, and P2 unbinding events at each constant pulling speed. **f** Izumo1:Juno rupture force *vs*. loading rates (median, error bars ± MAD). Dashed lines show the loading rate *vs*. rupture force behavior predicted by DHS model fitting (Eqs. (6) and (7), Supplementary Table 1). **g** Izumo1:Juno rupture force histograms (bins = 20 pN). Data in (**f** and **g**) represent four pooled independent experiments that were divided based on unbinding pathway for DHS model fitting (P0: *n* = 415, 202, 120, 212. P1: *n* = 121, 114, 171, 175. P2: *n* = 440, 566, 83, 175). For (**g**), significance (*p < 0.05) was determined using Kurskal–Wallis tests followed by pairwise Wilcoxon rank-sum tests, exact *P*-values are provided in the Source Data file. **h** Direct Izumo1:Juno unbinding (NI, force *vs*. extension curves: P0a, P1a, P1b, and P2b) compared to Izumo1:Juno unbinding through intermediate folded states (I, force *vs*. extension curves: P1c and P2c. Mean, error bars ± SEM). In (**h**), independent replicates were kept separate and further divided based on pathway and constant pulling speed (P0: *n* = 17-151. P1: *n* = 12-171. P2 *n* = 2–100, exact *n*-values provided in the Source Data file). For (**h**), significance (*p < 0.05) was determined using Kurskal–Wallis tests followed by Dunn tests (Šidák-adjusted), *P*-values: 0.031 (P0 vs *P*1), 0.008 (P0 vs P2), 0.684 (P1 vs P2). Full statistical details are provided in the “Methods”.

In mammals, gamete cell fusion is contingent on the remarkably specific cellular adhesion mechanisms that exist between sperm and egg cells^1–3^. Where several fusion-indispensable genes have now been identified^3–6^, sperm-egg adhesion is still primarily defined by the interaction between the sperm transmembrane protein Izumo1^1^ and the egg peripheral membrane protein Juno (also known as Folr4 or Izumo1r)^2^ (Fig. 1a). Despite the involvement of various membrane-associated proteins in the fertilization process, only the Izumo1:Juno complex has been shown to be necessary for gamete cell fusion and successful fertilization^1,2,7,8^. Yet, to date, only limited insights into the molecular mechanisms underpinning Izumo1:Juno-mediated adhesion have been obtained from conventional structural and biophysical approaches^2,9–11^. Further impeding our understanding of human infertility has been the absence of any biophysical characterization of infertility-associated Izumo1 or Juno variants.

From a mechanobiology perspective, it is evident that Izumo1:Juno must be sufficiently mechanostable to resist the powerful undulating forces generated by the sperm flagellum (Fig. 1b). Regularly exceeding 100 pN in primates^12^, these forces are sufficient to rupture the vast majority of eukaryotic protein:protein interactions studied to date^13^. The selective pressure for highly mechanostable protein interactions is manifested in several interesting ways^14–18^. Of particular interest in the field of cellular adhesion is the catch bond^19–21^. Canonically, protein interactions are observed as slip bonds; when force is applied to a slip bond, the bond lifetime decreases exponentially. Conversely, in protein interactions exhibiting catch bond behavior, the application of force increases the bond lifetime^22^. Previous examples of molecular mechanisms that govern catch bond behavior include force-induced allosteric changes^23,24^, alterations to hydrogen bonding networks and contact surface areas^25–27^, changes to force propagation pathways^28^, the presence of multiple binding modes^29^, and the availability of alternate unbinding pathways^23,30,31^. It is through these subtle structural mechanisms that catch bonds facilitate the emergence of unique force-dependent behaviors at the supramolecular and cellular levels^32^. In considering scenarios for gamete cell fusion, it becomes clear that catch bonding might not only stabilize sperm-egg adhesion, but in doing so directly influence the selection of motile sperm^33^.

Here, by combining atomic force microscopy-based single-molecule force spectroscopy (AFM-SMFS) with kinetic Monte Carlo modeling and all-atom steered molecular dynamics (SMD) simulations, we show that the unbinding behavior of Izumo1:Juno is indeed consistent with that of a remarkably stable multi-state catch bond^23,31,34^. In doing so, we provide molecular insights into a previously unexplained infertility-associated mutation (JunoH177Q)^35^, and identify a secondary, non-equilibrium, binding interface for consideration in drug development.

## Results

### Constant speed AFM-SMFS of the Izumo1:Juno complex

To study how the human Izumo1:Juno complex responds to force, we first expressed the glycosylated ectodomains (ECD) of Izumo1 and Juno using Sf9 cells. The presence of C-terminal SpyTags were used to confirm the successful expression and purification of Izumo1 and Juno, which enabled highly sequence-specific isopeptide bond formation with SpyCatcher containing polyproteins, as observed through SDS-PAGE analysis. These C-terminal SpyTags were subsequently used in the covalent attachment of the polyproteins required for AFM-SMFS, termed AFM-handles. For the AFM-SMFS experiment described in Fig. 1, the AFM-handles were expressed in *E. coli* and consisted of SpyCatcher, a ddFLN4 fingerprint domain, an elastin-like polypeptide (ELP) linker, and a ybbR tag for site-specific surface immobilization. A flow cytometry-based bead-binding assay was first used to confirm the equilibrium binding affinity (*K*_D_) and validate recombinant protein quality for all purified Izumo1 and Juno variants in the presence of the AFM-handle and following our surface immobilization procedure (Supplementary Fig. 1). For our recombinantly expressed wild-type Izumo1:Juno complex, we measured a *K*_D_ of 45 ± 5 nM (± SE), which was in excellent agreement with previously published surface plasmon resonance data^9^.

We next measured the loading rate dependent rupture force distributions of Izumo1:Juno using constant speed AFM-SMFS. Here, cantilevers functionalized with Izumo1 were briefly brought into contact with cover glasses functionalized with Juno (Fig. 1c). Following this brief contact (0.2 s), the cantilever was retracted at a constant speed (800, 1600, 3200, or 6400 nm·s^−1^). Both Izumo1 and Juno were site-specifically and covalently linked to their respective surfaces through their C-termini, resulting in a pulling geometry that precisely mimicked their natively anchored orientation at the cell surface (i.e., attachment through the transmembrane and intracellular domain (TID) of Izumo1 and the glycosylphosphatidylinositol (GPI) anchor of Juno). When Izumo1:Juno complexes formed, retraction of the cantilever resulted in stretching and a build-up of tension that was recorded in force *vs*. extension curves. Sawtooth-like peaks in the force *vs*. extension curves indicated the sudden release of tension through unfolding of the ddFLN4 fingerprint domains, conformational changes within Izumo1 or Juno, or the eventual rupture of the Izumo1:Juno complex (Fig. 1d, top panels). We note here that the unfolding of two ddFLN4 fingerprint domains generates four characteristic sawtooth-like peaks, typically within the 50–80 pN range. This distinct mechanical signature enables reliable identification of specific single-molecule Izumo1:Juno interactions and assists in excluding force *vs*. extension traces that may contain multiple Izumo1:Juno interactions or other erroneous signals (e.g., surface functionalization artifacts). By repeating these measurements tens of thousands of times, we could filter explicitly for a rich dataset of single Izumo1:Juno complex formation and rupture events. Further explanation of the force *vs*. extension curve selection procedure and representative examples (Supplementary Fig. 2) are provided in the “Methods” and Supplementary Text.

### Wild type Izumo1:Juno unbinds through three distinct pathways

At constant pulling speeds, Izumo1:Juno predominantly unbinds along three distinct pathways, observed in Fig. 1f, g as a trimodal rupture force distribution. We refer to these characteristic unbinding behaviors as pathway 0 (P0), pathway 1 (P1), or pathway 2 (P2). P1 and P2, in particular, are also associated with intermediate unfolding events within the Izumo1:Juno complex (see below). The discrete pathway switching of Izumo1:Juno is a phenomenon separate from the classical loading rate dependency of rupture forces described by the Bell-Evans (BE) model and its extensions, which includes the Dudko–Hummer–Szabo (DHS) model^36–39^. Nonetheless, within each unbinding pathway, we still observe rupture forces that increase with loading rate. P0, P1, and P2 exhibit median rupture forces of 32.3–39.9 pN for loading rates of 1.2–16.9 nN·s^−1^, 87.7–132.5 pN for loading rates of 3.0–36.5 nN·s^−1^, and 346.2–499.8 pN for loading rates of 22.1–121.5 nN·s^−1^, respectively (Fig. 1f). Across the trimodal rupture force distribution, the proportion of Izumo1:Juno complexes unbinding through either P0, P1, or P2 displayed a non-linear dependence on pulling speed. Separately fitting each of the four independent experiments using a Gaussian mixed model (GMM), we observed that as pulling speed increased from 800 to 6400 nm·s^−1^, the pathway dominance of P0 in Izumo1:Juno decreased from 68.7 ± 8.3 to 23.1 ± 8.8% (± SEM). In parallel, unbinding through P1 increased from 31.3 ± 8.4 to 47.9 ± 5.8% (± SEM), and unbinding though P2 increased from 0 ± 0 to 28.9 ± 3.8% (± SEM). Based on these experimental P0, P1, and P2 distributions, we propose that P0 is representative of an initial near equilibrium unbinding configuration, whilst P1 and P2 result from sequential force-induced structural changes to the Izumo1:Juno complex.

### Higher order unbinding pathways desensitize Izumo1:Juno to mechanical stress

We next used the DHS and BE models to describe the underlying energy landscapes governing P0, P1, and P2^36–39^. The key distinction in the theoretical energy landscapes described by the DHS and the BE models relates to their treatment of Δ *x*, which represents the distance to the transition state and determines how force accelerates bond rupture. In the DHS model, Δ *x* is assumed to be force-dependent, whilst in the BE model Δ *x* is assumed to be constant and therefore independent of force. For both DHS and BE fitting of P0, P1, and P2 rupture events, we observed a several-fold decrease in Δ*x* when comparing P1 to P0 and then again when comparing P2 to P1 (Supplementary Table 1). These changes to Δ *x* indicate an increasing rigidity to the Izumo1:Juno interaction along P1 and P2, implying greater cooperativity during unbinding. In this way, successive pathway-dependent changes in Δ*x* are consistent with loading rate dependent catch bond behavior^34^.

During Izumo1:Juno unbinding, P1 and P2 exist only as force-activated pathways. In this scenario, extrapolation of the DHS and BE off-rate to loading rates of zero (*k*_0_) is not particularly meaningful. Instead, we calculated the DHS and BE-derived force-dependent off-rates (*k(F)*) for P0, P1, and P2. We observed that despite a lower *k*_0_, the P0 *k(F)* overtakes the P1 *k(F)* at forces of ~5–20 pN (Supplementary Fig. 3). Similarly, differences in the DHS and BE-derived *k(F)* for P1 and P2 become apparent above ~20 pN, with P2 showing greater stability at higher forces (Supplementary Fig. 3). Overall, despite operating under different assumptions, the DHS and BE models show strong agreement regarding force-dependent Izumo1:Juno dissociation. Together, they support the interpretation that P0 represents a near-equilibrium conformation and that P1 and P2 are representative of conformational changes that enhance the stability of the Izumo1:Juno complex over biologically relevant force regimes.

### Higher order Izumo1:Juno unbinding pathways are linked to large conformational changes

Across the constant speed AFM-SMFS Izumo1:Juno datasets, we also identified the presence of several intermediate Izumo1:Juno folded states. These intermediate Izumo1:Juno folded states manifest in force *vs*. extension curves as additional sawtooth-like peaks and contour length increments, distinct from the characteristic ddFLN4 fingerprint unfolding patterns (Fig. 1d). To further explore these observations and their relationship to P0, P1, and P2, we subdivided the P0, P1, and P2 force *vs*. contour length curves based on the presence of these intermediates (Supplementary Fig. 2). For each constant pulling speed, the resulting proportions of Izumo1:Juno complexes unbinding directly (no intermediate (NI); curve subtypes: P0a, P1a, P1b, and P2b) and those unbinding through an intermediate state (intermediate (I); curve subtypes: P1c and P2c) are shown in Fig. 1h. In P1, we observed transition through intermediate folded states increasing from 14.1 ± 9.4 to 22 ± 5% (± SEM) as the pulling speed increased from 800 to 6400 nm·s^−1^. Whilst in P2, we observe transition through intermediate folded states increasing from 33.3 ± 20.4 to 66.3 ± 10.2% (± SEM) over the same range of pulling speeds. In P1 and P2, these intermediate folded states appeared at median loading rates between 5.1 and 33.9 nN·s^−1^ and 6.4 and 45.6 nN·s^−1^, respectively. This loading rate dependence in P1 and P2 strongly supports their interpretation as distinct, pathway-specific mechanistic states rather than measurement artifacts. Whereas for P0, despite reaching median loading rates of 16.9 nN·s^−1^, we typically observed direct unbinding without the appearance of any intermediate folded states (see Supplementary Text). At pulling speeds of 3200 nm·s^−1^, the pathway-induced intermediate unfolding events have median contour length intervals of 14.8 ± 6.7 and 15.1 ± 8.1 nm (± MAD) for P1 and P2, respectively. At 6400 nm·s^−1^, the median contour length intervals increased to 15.1 ± 6.9 and 19.2 ± 9.1 (± MAD) for P1 and P2, respectively. Due to the presence of multiple interconnected disulfide bonds, it was not possible to assign these intermediates to discrete structures within the domains of Izumo1 or Juno. For reference, the distribution of the contour length increments associated with the Izumo1:Juno intermediates at all pulling speeds and a topology diagram of the disulfide networks of Izumo1 and Juno are provided in Supplementary Fig. 4.

### Force-stabilization of intermediate structure and dissipation mechanism(s) observed by all-atom SMD

**Fig. 2:**
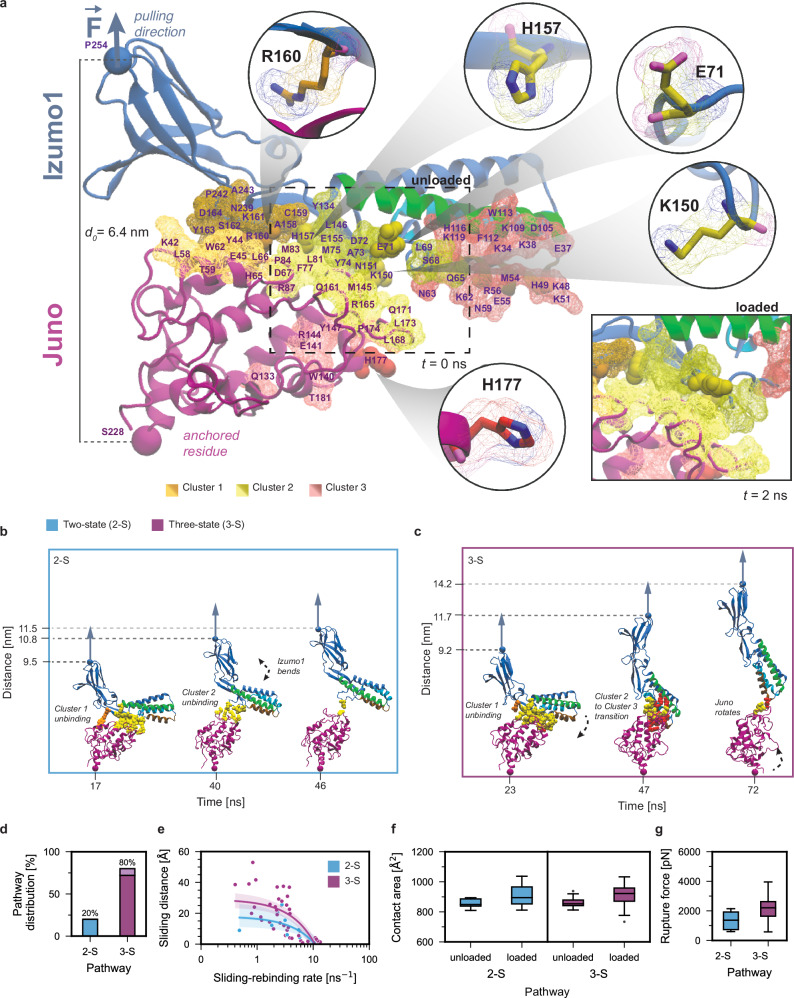
SMD simulations of Izumo1:Juno unbinding. **a** Known Izumo1:Juno binding interface shown in orange (Cluster 1) and yellow (Cluster 2), with the secondary binding interface shown in red (Cluster 3) (Supplementary Table 3). Circles highlight specific Izumo1 and Juno residues used in mutational studies. **b** Example of Izumo1:Juno unbinding along the known binding interface as part of the two-state (2-S) pathway. **c** Example of Izumo1:Juno forming the Cluster 3 interactions during unbinding as part of the three-state (3-S) pathway. In (**b** and **c**), the starting distance between C-terminal residues of the equilibrated Izumo1:Juno complex is 6.8 nm. **d** The two-state and three-state unbinding pathway distribution across 50 SMD trajectories. **e** Sliding-rebinding distance *vs*. sliding-rebinding rate for the force-dependent rearrangement of Izumo1:Juno complexes in the two-state and three-state unbinding pathways (shaded areas, 95% CI). **f** Observed increases in contact area between Izumo1 and Juno in unloaded (*t* = 0 ns) and loaded (*t* = 2 ns, inlet **a**) complexes for the two-state and three-state unbinding pathways. **g** Peak forces extracted from two-state and three-state SMD simulations of Izumo1:Juno complexes. For (**d**–**g**), *n* = 10 and *n* = 10 for 2-S and 3-S, respectively. Boxplots show the median, interquartile range (IQR), and whiskers extending to 1.5 times the IQR. Points beyond the whiskers are outliers.

To gain high-resolution structural insights, all-atom explicit solution SMD simulations^40,41^ of the Izumo1:Juno complex were performed at constant pulling velocities of 0.001 Å·ps^−1^. To best mimic the experiments, the C-terminal residue of Juno (S228) was fixed, and the C-terminal residue of Izumo1 acted as the pulling point (P254) (Fig. 2a). Simulations were then run in 50 independent replicas for 90 ns. For a summary of all simulations performed see Supplementary Table 2. The resulting SMD simulations describing force-dependent Izumo1:Juno unbinding were then analyzed with several computational tools (see below). Approximately 20% of the trajectories were classified as two-state unbinding (Fig. 2b, Supplementary Figs. 5 and 6, and Supplementary Movie 1), while ~80% were classified as three-state unbinding (Fig. 2c, d, Supplementary Figs. 7 and 8, and Supplementary Movie 2). Each state is defined by the interaction of three different clusters of amino acids on Izumo1:Juno (Supplementary Table 3). In State 1 (i.e., the equilibrium structure), Clusters 1 and 2 are bound; in State 2, only Cluster 2 is bound; in State 3, only Cluster 3 is bound. The time-dependent change for contacts between Izumo1 and Juno is shown in Supplementary Fig. 9.

Despite only minor secondary structural alterations to Izumo1 and Juno in the two-and three-state unbinding trajectories, the distance between the C-termini of Izumo1 and Juno show marked increases at several time points. Firstly, as Cluster 1 unbound, the Immunoglobulin-like domain of Izumo1 was pulled away from the binding interface (Fig. 2b, c). Secondly, as Cluster 2 unbound, the hinge-domain of Izumo1 was also pulled away from the binding interface (Fig. 2b, c). Thirdly, as Izumo1 straightened, it underwent progressive sliding and rebinding across the surface of Juno (Fig. 2f). This sliding-rebinding process was greatly prolonged by the gradual binding of Cluster 3 in the three-state unbinding trajectories, further increasing the distance between the C-termini of Izumo1 and Juno.

Across the 50 SMD replicas, the median distance change between the C-termini of Izumo1 and Juno was 5.7 ± 1.6 and 6.4 ± 0.9 nm (± MAD), for the two-and three-state unbinding trajectories, respectively. Additionally, in 4% of Izumo1:Juno SMD simulations, we observed partial unfolding of the Juno C-terminal α-helix (Supplementary Figs. 4f and 10). Unfolding of this Juno α-helix until the proximal disulfide bond would contribute an additional contour length increment of 7.7 nm (residues 207–228, 21 aa × 0.365 nm). Due to the extremely high pulling speeds necessary for all-atom SMD simulations, we propose that Juno’s C-terminal α-helix unfolding occurs less frequently than it might in the experimental setup. This discrepancy likely arises due to the shorter timescales in all-atom SMD simulations, which limit the ability of the system to explore slower, otherwise experimentally accessible unfolding pathways. Indeed, when considering the inherent differences between constant speed AFM-SMFS and all-atom SMD, the median contour lengths for the experimentally observed Izumo1:Juno folded states (14.8–19.2 nm, Supplementary Fig. 4) are consistent with the combined median distance intervals for all-atom SMD observed Izumo1:Juno rearrangements and Juno unfolding (13.4–14.1 nm).

Prior to Cluster 1 unbinding, the contact area between Izumo1 and Juno increased as the Izumo1:Juno complex transitioned from the unloaded state (*t* = 0 ns) to the loaded state (*t* = 2 ns). For the two-state unbinding trajectories, the median contact surface increased from 848.7 ± 25 to 894.9 ± 62.9 Å^2^ (± MAD) (Fig. 2f), while for the three-state unbinding trajectories, the median contact surface area increased from 883.4 ± 24.7 to 921.9 ± 58.5 Å^2^ ( ± MAD) (Fig. 2f). Subsequent unbinding of Izumo1:Juno from State 2 resulted in median peak forces of 1372 ± 910.1 pN (± MAD) (Fig. 2g). With the gradual binding of Cluster 3 and transition to State 3 (Fig. 2c), the protracted period of sliding and rebinding resulted in a 61% increase in the median peak forces for the three-state unbinding trajectories, to 2210 ± 739.5 pN (± MAD) (Fig. 2g). In considering all above analyses, we propose that the two-state pathway is representative of P1 and that the three-state pathway is representative of P2.

Finally, we note that the three-state unbinding trajectories can involve divergent residues on Izumo1 and Juno. We identified a subtype comprising ~10% of the three-state unbinding trajectories (Fig. 2d, Supplementary Figs. 11 and 12, and Supplementary Movie 3). In this sub-pathway, the partially buried Izumo1 residues implicated in its membrane fusion function (F28, W88, and W113)^11^ underwent a corkscrew-like motion, aligning towards the same interaction plane with increased solvent-exposed surface area (Supplementary Fig. 12 and Supplementary Movie 4). This finding, alongside other force-dependent structural rearrangements (see Supplementary Text and Supplementary Fig. 13), could help explain the unexpectedly low fusion rates for Izumo1 expressing cells under equilibrium conditions^11^. Thus, mechanical force should be considered as a relevant parameter for future Izumo1 membrane fusion studies.

### Functional redundancy of higher order Izumo1:Juno unbinding pathways

To pinpoint crucial residues, we asked how Izumo1 point mutations might influence P0, P1, and P2. We introduced single alanine substitutions into well conserved residues within Clusters 1 (Izumo1 R160A) or 2 (Izumo1 H157A)^9^, as well as two divergent residues within Cluster 2 (Izumo1 E71A and Izumo1 K150A)^9^ (Fig. 2a). Using our bead-based flow cytometry method, we observed increases in *K*_D_ for Izumo1 E71A:Juno, Izumo1 H157A:Juno, and Izumo1 R160A:Juno, relative to the wildtype, that were consistent with prior work (Supplementary Fig. 1)^9^. For the previously uncharacterized interaction, Izumo1 K150A:Juno, we measured a *K*_D_ of 110 ± 27 nM (SE).

Overall, using the same constant speed AFM-SMFS setup described in Fig. 1c, the unbinding pathways of the selected Izumo1 mutants did not deviate in terms of the median rupture forces or Δ *x* trends described for wild-type Izumo1:Juno (Supplementary Figs. S14–17; Supplementary Table 1). Thus, it becomes clear that while alanine substitutions of single Izumo1 residues are sufficient to disrupt the equilibrium binding behavior of Izumo1:Juno (Supplementary Fig. 1)^9^, the mechanical behavior and force-dependent unbinding pathways offer a greater level of functional redundancy. Where disruption of the mechanical behavior is observed, it is largely confined to the unbinding pathway distributions and the Izumo1:Juno intermediate folded states (Supplementary Figs. S14–17), thereby implicating the invisible transition barriers separating P0, P1, and P2, and changes in force propagation (see Supplementary Text).

### Monte Carlo force spectroscopy simulations support the multi-state catch bond mechanism

**Fig. 3:**
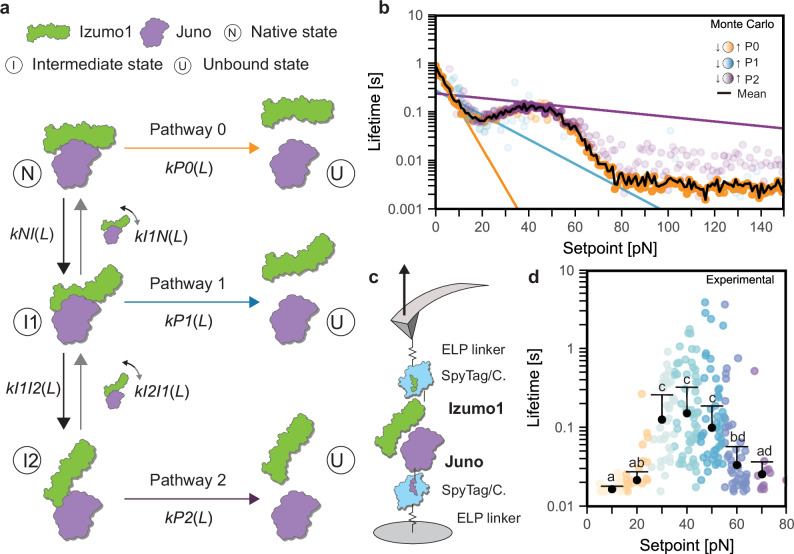
Modeling and validation of Izumo1:Juno forming a catch bond. **a** Proposed three-state kinetic model for unbinding of Izumo1:Juno with reversible transitions between multiple structural states. **b** Monte Carlo simulated force clamp AFM-SMFS for the Izumo1:Juno complex. Colored dots represent mean bond lifetime for each unbinding pathway at each setpoint with intensity representing the relative frequency of an unbinding pathway. Colored lines represent lifetimes calculated directly from BE-derived kinetic parameters (Supplementary Table 1, Eq. (11)). Black line represents the mean bond lifetime across all three pathways. **c** Schematic showing protein constructs and surface immobilization for force clamp AFM-SMFS. **d** Experimental bond lifetimes for Izumo1:Juno from force clamp AFM-SMFS (representative of the consensus across the three biological replicates performed). Colored dots represent individual bond lifetime measurements performed. Black dots represent median bond lifetime (error bars ± MAD) across 10 pN bins (*n* = 18, 18, 33, 53, 44, 38, 9). Shared letters indicate the absence of statistically significant differences (*p* > 0.05) from Kruskal–Wallis tests followed by Dunn’s multiple-comparison tests (Šidák-adjusted). Exact *P*-values are provided in the Source Data file. Full statistical details are provided in the “Methods”.

Multi-state kinetic Monte Carlo force spectroscopy simulations^14,29^ were next used to describe the mechanically induced dissociation of Izumo1:Juno and evaluate competing catch bond models. Based on the experimental constant speed AFM-SMFS datasets showing three unbinding pathways and the SMD simulations showing up to three distinct Izumo1:Juno states, we compared two models each with three rupture pathways and three states (Fig. 3a, native (N), intermediate 1 (I_1_), and intermediate 2 (I_2_)). One model simulated irreversible transitions and the other simulated reversible transitions from N to I_1_ and from I_1_ to I_2_. To ensure computational feasibility, whilst still capturing the force-dependent features of Izumo1:Juno unbinding, we used the experimentally derived BE parameter sets pertaining to P0, P1, and P2 (Supplementary Table 1). The barriers separating each state (i.e., N ↔ I_1_ ↔ I_2_ or N → I_1_ → I_2_) are experimentally invisible; meaning they do not manifest as distinct, observable pauses or dwell times or unfolding events in the force *vs*. extension curves; they were evaluated for each catch bond model by algorithmically testing millions of possible BE parameter sets within constant speed Monte Carlo force spectroscopy simulations. The simulated rupture force distributions were then compared to the experimental rupture force distributions to determine the best fit (Supplementary Tables 4 and 5). From these simulations, we conclude that the model allowing for reversible transitions (as opposed a non-reversible transition model) was able to more closely recapitulate the experimental rupture force distributions, and was therefore the most representative kinetic model (Supplementary Fig. 18).

The reversible transition model was then used in Monte Carlo force spectroscopy simulations of Izumo1:Juno unbinding under constant force conditions (i.e., force clamp) (Fig. 3b). Our reversible three-state model predicted that the Izumo1:Juno slip-to-catch transition sets in at ~20 pN, and is overtaken by a subsequent catch-to-slip transition at ~40 pN. This predicted transition occurs near to the previously reported free swimming force of spermatocytes of 44 pN, which does not necessarily act normal to the membrane surface^42^. The simulations show that from 20 to 40 pN, P2 was the most frequent unbinding pathway at rupture. While, at very low (<20 pN) and very high forces (>80 pN), P0 was found to dominate. We also simulated clamping forces up to 150 pN to encompass the highest transverse forces recorded for hyperactivated sperm cells^12^, such as would be present for sperm cells bound to egg cells. Interestingly, our Monte Carlo force clamp simulations predict a broad period of stability influenced by sequestering in I_2_ before eventual rupture through P0. These observations highlight the potential interplay of all three unbinding pathways in stabilizing Izumo1:Juno across different biologically relevant force regimes.

### Force clamp AFM-SMFS observation of the Izumo1:Juno catch bond behavior

Force clamp AFM-SMFS was then used to experimentally capture Izumo1:Juno catch bond behavior and validate our Monte Carlo force clamp force spectroscopy simulations. For each individual force clamp measurement, the cantilever first approached the surface, then following a brief contact with the surface to facilitate Izumo1:Juno binding (0.2 s), the cantilever was retracted at 4000 nm·s^−1^ until the target force setpoint was reached. The force setpoint was then maintained using a feedback loop that adjusted the height of the cantilever until bond rupture. The ddFLN4 fingerprint domains were omitted from the force clamp AFM-SMFS protein constructs to avoid biasing bond lifetime measurements at low forces (Fig. 3c). A detailed description of the curve selection procedure is provided (see “Methods”), which included replicates of Izumo1:blank controls to evaluate non-specific bond clamping rates (Supplementary Fig. 19). Representative force clamp curves are shown in Supplementary Fig. 20. All force clamp AFM-SMFS experiments were performed in biological triplicate. Furthermore, to ensure sufficient sampling of each force setpoint for statistical analysis, the low-force (10–80 pN) and the high-force (80–160 pN) regimes were measured separately.

In agreement with the force clamp Monte Carlo force spectroscopy simulations, the force clamp experiments show statistically significant increases in bond lifetime beginning at ~20 pN and peaking at 40–50 pN (Fig. 3d and Supplementary Fig. 19). Over the slip-to-catch transition, we observed a 7.5-fold increase in median bond lifetime from 0.02 ± 0.003 to 0.15 ± 0.02 s (mean ± SEM) (Fig. 3d and Supplementary Fig. 19). In the high-force regime, at clamping forces from 80 to 160 pN, we observed approximately the same bond-clamping yield with no statistically significant difference between the bond lifetimes (representative examples: Supplementary Fig. 19e, f). These results provide direct agreement with the force clamp Monte Carlo force spectroscopy predictions regarding the emergence of the catch bond state at forces paralleling the free swimming force of spermatocytes^42^. Further, they provide support for the predicted broad period of stability at the transverse forces typical of hyperactivated sperm cells on the egg cell membrane^12^. Overall, the self-consistency and broad agreement across different experimental AFM-SMFS and Monte Carlo force spectroscopy simulation approaches used in this study serves as further validation of the initial experimental AFM-SMFS constant speed interpretations. In particular, that P1 and P2 arise through force-dependent processes as part of Izumo1:Juno catch bond behavior.

### Loss of mechanostability in the infertility-associated Juno mutant, JunoH177Q

To explore the role of mechanostability in human fertility, we characterized the infertility-associated mutant JunoH177Q. This particular variant was found in 9% of patients across a female idiopathic infertility cohort^35^. Since the initial publication, the prevalence of the JunoH177Q variant has been measured at 0.15–0.18% in populations where greater than 150 Juno alleles have been sequenced (dbSNP database: rs76779571). Notably, Juno H177 is located distal to the equilibrium Izumo1:Juno binding interface and our flow cytometry-bead based binding assay demonstrates a high binding affinity (*K*_D_ = 64 ± 14 nM (± SE)) for Izumo1:JunoH177Q. Thus, equilibrium-based analysis and structural equilibrium data for the Izumo1:Juno complex^9,43–45^ do not explain any deleterious effects of this infertility-associated mutation.

**Fig. 4:**
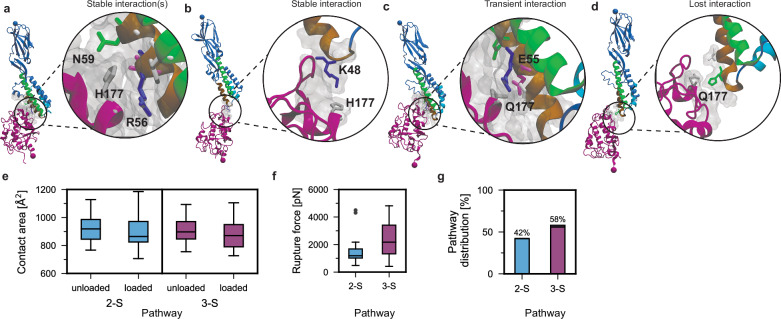
Juno H177 mediates the force-initiated transition to higher order unbinding pathways in Izumo1:Juno. **a** Exemplary snapshot of stable long-lasting Cluster 3 interactions mediated by Juno H177. **b** Exemplary snapshot of terminal complex-prolonging interactions mediated by Izumo1 K48 and Juno H177. **c** Exemplary snapshot of transient Cluster 3 interactions formed in the Izumo1:JunoH177Q infertility mutant. **d** Exemplary snapshot of terminal interactions, the last ones to break prior rupture, that are absent in Izumo1:JunoH177Q. **e** Observed decreases in contact area as derived from SMD between Izumo1 and JunoH177Q in unloaded (*t* = 0 ns) and loaded (*t* = 2 ns) complexes for the two-state (2-S) and three-state (3-S) unbinding pathways. **f** Peak forces extracted from two-state and three-state SMD simulations of Izumo1:JunoH177Q complexes (median ± MAD). **g** Two-state and three-state unbinding pathway distribution for Izumo1:H177Q across 50 SMD trajectories. For (**e**–**g**), *n* = 21 and *n* = 29 for 2-S and 3-S, respectively. Boxplots show the median, interquartile range (IQR), and whiskers extending to 1.5 times the IQR. Points beyond the whiskers are outliers.

Within our SMD classification framework, Juno H177 is a Cluster 3 amino acid that interacts with Izumo1 as part of the three-state unbinding pathway (Fig. 2a, c). In the protracted sliding-rebinding mechanism that defines State 3, Juno H177 forms both early and terminal interactions, first with Izumo1 N59&R56 (Fig. 4a) and later Izumo1 K48 (Fig. 4b). Across 50 SMD simulations of Izumo1:JunoH177Q unbinding, the stable interactions between Izumo1 N59&R56-JunoH177 and Izumo1K48-JunoH177 are either replaced with transient interactions or lost completely (Fig. 4c, d and Supplementary Fig. 21). While the peak forces during the three-state unbinding of Izumo1:JunoH177Q are similar to wild-type Izumo1:Juno, we observed a 5.9% decrease in the median contact surface area when comparing the unloaded (*t* = 0 ns) to the loaded states (*t* = 2 ns), from 918.6 ± 81.8 to 864.1 ± 95.7 Å^2^ (± MAD) (Fig. 4e). This coincided with a 13% reduction in median peak force for the two-state unbinding trajectories (Fig. 4f), from 1372.4 ± 630.1 pN to 1190.1 ± 687.6 pN (± MAD) and a decrease in the prevalence of three-state unbinding trajectories, from 80% to 58% (Fig. 4g). Thus, we conclude that the stabilization of State 2 through force-dependent increases in contact surface area around Juno H177 facilitates Cluster 3 formation and catch bond activation in wild-type Izumo1:Juno.

Noteworthy, in SMD simulations where Juno H177 was mutated to a negatively charged glutamic acid residue (JunoH177E), the median peak force of the two-state unbinding trajectories increased by 55%, from 1372.4 ± 630.1 pN to 2129.9 ± 654.5 pN (± MAD) (Supplementary Fig. 22a). Additionally, for Izumo1:JunoH177E, we note the absence of the three-state unbinding subpathway (Supplementary Fig. S22b) linked to the solvent-exposure of membrane fusion-associated Izumo1 residues (Supplementary Fig. 12)^11^. With contact surface area increases between the unloaded (*t* = 0 ns) to the loaded states (*t* = 2 ns) of Izumo1:JunoH177E (Supplementary Fig. 22c), similar to wild-type Izumo1:Juno, the increased mechanostability of JunoH177E in State 2 and the absence of the three-state unbinding subpathway appears to be charge related. Finally, similar to JunoH177Q, for JunoH177E we observed an increase in unfolding rate for the C-terminal Juno α-helix (residues 207–228, Supplementary Fig. 4f), from 4% to 14% (Supplementary Fig. 10c). This increase in Juno unfolding corresponded to a slightly increased median distance between the C-termini of Izumo1:JunoH177Q and Izumo1:JunoH177E at rupture, from 12.9 ± 7.2 to 13.4 ± 7.5 and 12.4 ± 7.3 to 13.9 ± 7.4 nm (± MAD), respectively.

**Fig. 5:**
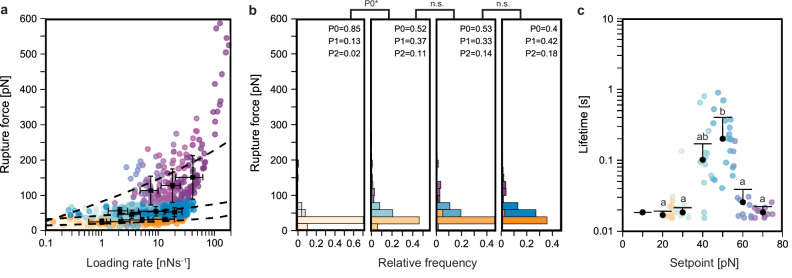
Constant speed and force clamp AFM-SMFS reveals the loss of mechanostability in Izumo1:JunoH177Q. **a** Rupture force *vs*. loading rate plot for Izumo1:JunoH177Q (median, error bars ± MAD). Dashed lines show the loading rate *vs*. rupture force behavior predicted by DHS model fitting (Eqs. (6) and (7), Supplementary Table 1). **b** Izumo1:JunoH177Q rupture force histograms (bins = 20 pN). Data in (**a** and **b**) represent four pooled independent experiments that were divided based on unbinding pathway for DHS model fitting (P0: *n* = 325, 145, 113, 266. P1: *n* = 113, 62, 116, 188. P2: *n* = 63, 73, 4, 39). For (**c**), significance (*p < 0.05) was determined using Kurskal–Wallis tests followed by pairwise Wilcoxon rank-sum tests. **c** Bond lifetimes for Izumo1:JunoH177Q from force clamp AFM-SMFS (representative of the consensus across the three biological replicates performed). Colored dots represent individual bond lifetime measurements. Black dots represent median bond lifetime (error bars ± MAD) across 10 pN bins (*n* = 1, 7, 10, 11, 17, 11, 9). Shared letters indicate the absence of statistically significant differences (*p* > 0.05) using Kruskal–Wallis tests followed by Dunn’s multiple-comparison tests (Šidák-adjusted). Exact *P*-values are provided in the Source Data file. Full statistical details are provided in the “Methods”.

Experimentally, constant speed AFM-SMFS shows that whilst three unbinding pathways can be classified for Izumo1:JunoH177Q using the GMM, the median rupture forces for P0, P1, and P2 have all decreased. Here, P0, P1, and P2 exhibit median rupture forces of 26.1–33.1 pN for loading rates of 1–12.3 nN·s^−1^, 48.5–59 pN for loading rates of 2.1–18.9 nN·s^−1^, and 114.2–115.9 pN for loading rates of 7.4–42.2 nN·s^−1^, respectively (Fig. 5a, b). The DHS and BE fitted Δ*x* values for P1 and P2 are several-fold higher than in wild-type Izumo1:Juno (Supplementary Table 1), implicating increased conformational flexibility in the loss of mechanostability of these pathways. The disruption of temporary sequestration of complexes within mechanostable intermediate states (Fig. 3a) could explain secondary effects on the mechanostability of P0 in the absence of large changes to Δ*x*^34^. In support of this hypothesis, we observed a lower prevalence of transition from P0 to P1 and P1 to P2 at all pulling speeds. We also observed an increased contour length change for P1 and P2 intermediate states that could be linked to an increased frequency of unfolding for the C-terminal Juno helix (Supplementary Figs. 10c and 23c). The resulting median contour length changes amount to 19.9 ± 10 and 20 ± 9.8 nm (± MAD) for P1 and P2 at pulling speeds of 3200 nm·s^−1^, respectively (Supplementary Fig. 23c, d). Overall, constant speed AFM-SMFS results showing lower mechanostability, greater conformational flexibility, decreased transition through higher order unbinding pathways, and an increased contour length change for the intermediate states, all support the SMD results implicating Juno H177 in unbinding pathway transition and the overall mechanostability of Izumo1:Juno.

Monte Carlo force spectroscopy simulations at constant forces predict the preservation of catch bond behavior in Izumo1:JunoH177Q (Supplementary Fig. 23e). However, I_2_-dependent stabilization of mean bond lifetimes at 70–150 pN, as observed for wildtype Izumo1:Juno, is lost (Fig. 3b and Supplementary Fig. 23e). With the majority of the predicted bond lifetimes for Izumo1:JunoH177Q are below the detection threshold of our force clamp AFM-SMFS setup (0.02 s), we expected a lower bond-clamping yield and an over sampling of longer lifetime events. In force clamp AFM-SMFS of Izumo1:JunoH177Q, we observed catch bond behavior with an average median peak bond lifetime of 0.17 ± 0.04 s (± SEM) at 40–50 pN (Fig. 5c and Supplementary Fig. 23f, g). We were also unable to directly measure the loss of I_2_-dependent stabilization at 80–160 pN. However, in line with our force clamp Monte Carlo force spectroscopy predictions, cantilevers confirmed to be active showed fewer bond clamping events in 80–160 pN force range for Izumo1:JunoH177Q (representative example: Supplementary Fig. 23h) compared to wild-type Izumo1:Juno (representative examples: Supplementary Fig. 19e, f). Together, the SMFS experiments and simulations for Izumo1:JunoH177Q strongly support the hypothesis that this infertility mutation inhibits the effect of sequestering complexes in I_2_, thereby degrading the overall mechanostability.

## Discussion

In summary, we used constant speed and force clamp AFM-SMFS, combined with kinetic Monte Carlo modeling and all-atom SMD to discover and quantify three unbinding pathways of the Izumo1:Juno complex involved in human fertilization. We showed how loading rate-dependent transitions between these three conformational states gave rise to a multi-state catch bond that generates mechanostability across a broad force range. Unbinding at forces up to 600 pN in constant speed AFM-SMFS experiments, Izumo1:Juno represents one of the most mechanostable eukaryotic protein:protein interactions studied to date. Comparable examples of mechanostability having previously only been observed in muscle protein complexes involved in force transduction^13^ and bacterial receptors involved in matrix adhesion^25,26^ and cellulose degradation^29^.

The mechanical behavior of Izumo1:Juno is consistent with natural selection under the influence of two different sperm-egg binding scenarios. In the first-binding scenario, marked by the initial adhesion of freely swimming sperm to egg cells, healthy human sperm cells have a swimming force of approximately 44 pN^42^. This previously reported swimming force is remarkably consistent with the force at which the Izumo1:Juno catch bond state is most stable, experimentally and in our kinetic model. The second-binding scenario is characterized by the stable long-term adhesion between sperm and egg, directly preceding membrane fusion. Here, transverse forces generated by the flagellum of hyperactivated sperm cells can be on the order of 130 pN in primates^12^. In this force range, our kinetic model predicts that despite a return to slip bond behavior, the bond lifetime of intact Izumo1:Juno complexes is stabilized by temporary sequestering in highly mechanostable states (i.e., I_1_ and I_2_). The overall implication of our kinetic model is that any loss of sperm motility impairing the transition of Izumo1:Juno to I_1_ or I_2_ would inhibit both sperm-egg binding scenarios. Importantly, we also find that the infertility-associated mutant, JunoH177Q, is implicated in the proposed high-force stabilization mechanism. This mechanism involves a previously undiscovered Izumo1:Juno intermediate binding interface, not observed in the equilibrium crystal structure, that could represent a valid drug target for the development of both non-hormonal birth control and fertility treatments, which so far have had only limited success^46^.

Our work therefore demonstrates a highly mechanostable multi-state catch bond mechanism in Izumo1:Juno, identifies an additional non-equilibrium binding site for potential drug development, and provides biophysical and computational avenues for understanding human fertility.

## Methods

### Construct design and site-directed mutagenesis

#### Constructs for use in insect cell expression systems

The ectodomains of Izumo1 and Juno were cloned with a C-terminal His_10_ and SpyTag into pFastBac1 vectors for use in the Bac-to-Bac^TM^ Baculovirus expression system (Thermo Fisher Scientific, Massachusetts, United States). To this end, wild-type Izumo1-His_10_-SpyTag-LPETGG and Juno-His_10_-SpyTag-LPETGG were synthesized as codon-optimized linear DNA fragments (GeneArt—ThermoFisher Scientific, Regensburg, Germany) for Gibson Assembly using primers designed in SnapGene software. Izumo1 and Juno point mutations were generated as required using the Q5® Site-Directed Mutagenesis Kit and NEBuilder v1 software for primer design (New England Biolabs, Massachusetts, United States).

#### Constructs for use in bacterial expression systems

For biophysical characterization, the SpyTag/SpyCatcher system facilitated the covalent attachment of the polyproteins required for AFM-SMFS and flow cytometry binding studies. All constructs containing polyproteins required for the AFM-SMFS and flow cytometry binding studies were cloned in pET28a vectors for expression in *E. coli* NiCo21(DE3) (New England Biolabs, Massachusetts, United States) using Gibson Assembly and primers designed in SnapGene software. The construct (N-to C-terminus) used for surface functionalization in the constant speed AFM-SMFS and flow cytometry binding studies was ybbR-His_6_-ELP-(MV7E2)_3_-ddFLN4-SpyCatcher^47^. The construct (N-to C-terminus) used for surface functionalization in force clamp AFM-SMFS was ybbR-His_6_-ELP-(MV7E2)_3_-SpyCatcher. The above orientations of SpyCatcher in the constructs used in constant speed and force clamp AFM-SMFS is necessary to prevent undesired SpyCatcher unfolding. The construct (N-to-C-terminus) used for the fluorescent tagging in the flow cytometry binding studies was ybbR-HRV3C-His_6_-GFP-SpyCatcher. In each instance, the terminal ybbR-tag allowed for the site-specific immobilization of target proteins to surfaces coated in coenzyme A using surfactin phosphopantetheinyl transferase (SFP)^48^.

All constructs and point mutations were subsequently validated using Sanger sequencing (MicroSynth, Wein, Austria).

#### Full protein construct sequences

Where human rhinovirus (HRV) 3C Protease cleavage sites (LEVLFQGP) or sortase motifs (LPETGG) are present in the constructs, they were not used in this study. Further, the cysteine residue at position 18 in wild-type *Dictyostelium discoideum* 4th filamin domain (ddFLN4, UniProt: P13466, residues 549–649) has previously been mutated to C18S to avoid a potential cross-reaction to maleimide groups present from surface functionalization^47^. For human Izumo1 constructs, an N-terminal Hemolin (HEM) secretion signal peptide was added. For human Juno constructs, an N-terminal Honeybee Melittin (HBM) secretion signal peptide was added. For mouse Izumo1 and Juno constructs, an N-terminal Binding Immunoglobulin Protein (BiP) secretion signal peptide was added. Secretion signal peptides are required to direct recombinant proteins in *Spodoptera frugiperda* (Sf9) based cell lines to the secretory pathways. HEM and HBM include sites for proteolytic cleavage such that they are not present in the mature proteins. For both Izumo1 and Juno point mutants, the numbering is based on the consensus sequence provided by UniProt (Izumo1: Q8IYV9, Juno: A6ND01).

### HEM-Izumo1-His_10_-SpyTag-LPETGG

MAFKSIAVLSACIIVGSACVICDPSVVLALKSLEKDYLPGHLDAKHHKAMMERVENAVKDFQELSLNEDAYMGVVDEATLQKGSWSLLKDLKRITDSDVKGDLFVKELFWMLHLQKETFATYVARFQKEAYCPNKCGVMLQTLIWCKNCKKEVHACRKSYDCGERNVEVPQMEDMILDCELNWHQASEGLTDYSFYRVWGNNTETLVSKGKEATLTKPMVGPEDAGSYRCELGSVNSSPATIINFHVTVLPHHHHHHHHHHAHIVMVDAYKPTKLPETGG

### HEM-Izumo1E71A-His_10_-SpyTag-LPETGG

MAFKSIAVLSACIIVGSACVICDPSVVLALKSLEKDYLPGHLDAKHHKAMMERVENAVKDFQELSLNADAYMGVVDEATLQKGSWSLLKDLKRITDSDVKGDLFVKELFWMLHLQKETFATYVARFQKEAYCPNKCGVMLQTLIWCKNCKKEVHACRKSYDCGERNVEVPQMEDMILDCELNWHQASEGLTDYSFYRVWGNNTETLVSKGKEATLTKPMVGPEDAGSYRCELGSVNSSPATIINFHVTVLPHHHHHHHHHHAHIVMVDAYKPTKLPETGG

### HEM-Izumo1K150A-His_10_-SpyTag-LPETGG

MAFKSIAVLSACIIVGSACVICDPSVVLALKSLEKDYLPGHLDAKHHKAMMERVENAVKDFQELSLNEDAYMGVVDEATLQKGSWSLLKDLKRITDSDVKGDLFVKELFWMLHLQKETFATYVARFQKEAYCPNKCGVMLQTLIWCANCKKEVHACRKSYDCGERNVEVPQMEDMILDCELNWHQASEGLTDYSFYRVWGNNTETLVSKGKEATLTKPMVGPEDAGSYRCELGSVNSSPATIINFHVTVLPHHHHHHHHHHAHIVMVDAYKPTKLPETGG

### HEM-Izumo1H157A-His_10_-SpyTag-LPETGG

MAFKSIAVLSACIIVGSACVICDPSVVLALKSLEKDYLPGHLDAKHHKAMMERVENAVKDFQELSLNEDAYMGVVDEATLQKGSWSLLKDLKRITDSDVKGDLFVKELFWMLHLQKETFATYVARFQKEAYCPNKCGVMLQTLIWCKNCKKEVAACRKSYDCGERNVEVPQMEDMILDCELNWHQASEGLTDYSFYRVWGNNTETLVSKGKEATLTKPMVGPEDAGSYRCELGSVNSSPATIINFHVTVLPHHHHHHHHHHAHIVMVDAYKPTKLPETGG

### HEM-Izumo1R160A-His_10_-SpyTag-LPETGG

MAFKSIAVLSACIIVGSACVICDPSVVLALKSLEKDYLPGHLDAKHHKAMMERVENAVKDFQELSLNEDAYMGVVDEATLQKGSWSLLKDLKRITDSDVKGDLFVKELFWMLHLQKETFATYVARFQKEAYCPNKCGVMLQTLIWCKNCKKEVHACAKSYDCGERNVEVPQMEDMILDCELNWHQASEGLTDYSFYRVWGNNTETLVSKGKEATLTKPMVGPEDAGSYRCELGSVNSSPATIINFHVTVLPHHHHHHHHHHAHIVMVDAYKPTKLPETGG

### HBM-Juno-His_10_-SpyTag-LPETGG

MKFLVNVALVFMVVYISYIYADGDELLNICMNAKHHKRVPSPEDKLYEECIPWKDNACCTLTTSWEAHLDVSPLYNFSLFHCGLLMPGCRKHFIQAICFYECSPNLGPWIQPVGSLGWEVAPSGQGERVVNVPLCQEDCEEWWEDCRMSYTCKSNWRGGWDWSQGKNRCPKGAQCLPFSHYFPTPADLCEKTWSNSFKASPERRNSGRCLQKWFEPAQGNPNVAVARLFASHHHHHHHHHHAHIVMVDAYKPTKLPETGG

### HBM-JunoH177Q-His_10_-SpyTag-LPETGG

MKFLVNVALVFMVVYISYIYADGDELLNICMNAKHHKRVPSPEDKLYEECIPWKDNACCTLTTSWEAHLDVSPLYNFSLFHCGLLMPGCRKHFIQAICFYECSPNLGPWIQPVGSLGWEVAPSGQGERVVNVPLCQEDCEEWWEDCRMSYTCKSNWRGGWDWSQGKNRCPKGAQCLPFSQYFPTPADLCEKTWSNSFKASPERRNSGRCLQKWFEPAQGNPNVAVARLFASHHHHHHHHHHAHIVMVDAYKPTKLPETGG

### BiP-mIzumo1-His_10_-SpyTag-LPETGG

MKLCILLAVVAFVGLSLGCVICDPSVVLALKSLEKDYLPGHLDAKHHKAMMERVENAVKDFQELSLNEDAYMGVVDEATLQKGSWSLLKDLKRITDSDVKGDLFVKELFWMLHLQKETFATYVARFQKEAYCPNKCGVMLQTLIWCKNCKKEVHACRKSYDCGERNVEVPQMEDMILDCELNWHQASEGLTDYSFYRVWGNNTETLVSKGKEATLTKPMVGPEDAGSYRCELGSVNSSPATIINFHVTVLPGSGSGSGSHHHHHHHHHHGSGGLLDAHIVMVDAYKPTKLPETGG

### BiP-mJuno-His_10_-SpyTag-LPETGG

MKLCILLAVVAFVGLSLGGDELLNICMNAKHHKRVPSPEDKLYEECIPWKDNACCTLTTSWEAHLDVSPLYNFSLFHCGLLMPGCRKHFIQAICFYECSPNLGPWIQPVGSLGWEVAPSGQGERVVNVPLCQEDCEEWWEDCRMSYTCKSNWRGGWDWSQGKNRCPKGAQCLPFSHYFPTPADLCEKTWSNSFKASPERRNSGRCLQKWFEPAQGNPNVAVARLFASGSGSGSGSHHHHHHHHHHGSGGLLDAHIVMVDAYKPTKLPETGG

### ybbR-His_6_-ELP-(MV7E2)_3_-ddFLN4-SpyCatcher

MGTDSLEFIASKLAHHHHHHWGSGHGVGVPGMGVPGVGVPGVGVPGVGVPGVGVPGVGVPGVGVPGVGVPGEGVPGEGVPGVGVPGMGVPGVGVPGVGVPGVGVPGVGVPGVGVPGVGVPGVGVPGEGVPGEGVPGVGVPGMGVPGVGVPGVGVPGVGVPGVGVPGVGVPGVGVPGVGVPGEGVPGEGVPGWPSGSADPEKSYAEGPGLDGGECFQPSKFKIHAVDPDGVHRTDGGDGFVVTIEGPAPVDPVMVDNGDGTYDVEFEPKEAGDYVINLTLDGDNVNGFPKTVTVKPAPGSGSGSGSVDTLSGLSSEQGQSGDMTIEEDSATHIKFSKRDEDGKELAGATMELRDSSGKTISTWISDGQVKDFYLYPGKYTFVETAAPDGYEVATAITFTVNEQGQVTVNGKATKGDAHI

### ybbR-His_6_-ELP-(MV7E2)_3_-SpyCatcher

MGTDSLEFIASKLAHHHHHHWGSGHGVGVPGMGVPGVGVPGVGVPGVGVPGVGVPGVGVPGVGVPGVGVPGEGVPGEGVPGVGVPGMGVPGVGVPGVGVPGVGVPGVGVPGVGVPGVGVPGVGVPGEGVPGEGVPGVGVPGMGVPGVGVPGVGVPGVGVPGVGVPGVGVPGVGVPGVGVPGEGVPGEGVPGWPSGSVDTLSGLSSEQGQSGDMTIEEDSATHIKFSKRDEDGKELAGATMELRDSSGKTISTWISDGQVKDFYLYPGKYTFVETAAPDGYEVATAITFTVNEQGQVTVNGKATKGDAHI

### ybbR-HRV3C-His_6_-GFP-SpyCatcher

MGTDSLEFIASKLALEVLFQGPLQHHHHHHPWTSASSGGEELFAGIVPVLIELDGDVHGHKFSVRGEGEGDADYGKLEIKFICTTGKLPVPWPTLVTTLCYGIQCFARYPEHMKMNDFFKSAMPEGYIQERTIQFQDDGKYKTRGEVKFEGDTLVNRIELKGKDFKEDGNILGHKLEYSFNSHNVYIRPDKANNGLEANFKTRHNIEGGGVQLADHYQTNVPLGDGPVLIPINHYLSTQTKISKDRNEARDHMVLLESFSACCHTHGMDELYRGSGSGSGSVDTLSGLSSEQGQSGDMTIEEDSATHIKFSKRDEDGKELAGATMELRDSSGKTISTWISDGQVKDFYLYPGKYTFVETAAPDGYEVATAITFTVNEQGQVTVNGKATKGDAHI

### Protein expression and purification

#### Bacterial protein expression and purification

All pET28a constructs were transformed into *E. coli* NiCo21(DE3) for subsequent expression and purification. Cultures were grown in terrific broth media (TB, 12 g/L tryptone, 24 g/L yeast extract, 4 mL/L glycerol, 170 mM M KH2PO4, 720 mM K2HPO4, pH 7.0) at 37 °C with shaking until the O.D.600 of culture reached 0.6–0.7 where they were induced using 1 mM isopropyl β-d-1-thiogalactopyranoside (IPTG) at 16 °C, with shaking. After an induction period of 16 h, cultures were harvested using centrifugation and stored at −80 °C until needed. Cell pellets were resuspended at 4 °C in lysis buffer (50 mM Tris-HCl, 50 mM NaCl, 5 mM MgCl_2_, 0.1% (v/v) TritonX-100, Glycerol, pH 8.0) for subsequent sonication (Digital Sonifier 450, Branson Ultrasonics Corp, Connecticut, United States). The cell lysate then underwent centrifugation at 30,000 × *g* for 30 min and was passed through a 0.45 µM filter. The clarified lysate was then incubated with Ni-NTA Resin (HisPur™, Thermo Fischer Scientific, Massachusetts, United States) for 1 h at 4 °C with shaking. Ni-NTA Resin was reconstituted by centrifugation 800 × *g* for 5 min and transferred to gravity flow columns (Pierce™, Thermo Fischer Scientific, Massachusetts, United States). The resin was then washed extensively in 10 mM imidazole-supplemented phosphate-buffered saline (PBS, 137 mM NaCl, 2.7 mM KCl, 10 mM Na_2_HPO_4_, and 1.8 mM KH_2_PO_4_, pH 7.4) and eluted in 250 mM imidazole-supplemented PBS. Protein-containing elution fractions were concentrated in centrifugal filters (Amicon^®^, Merck, Darmstadt, Germany) and further processed using size-exclusion chromatography (SEC, Superdex 200 Increase 10/300 GL column, Cytiva, Marlborough, United States) into PBS. SEC elution fractions containing the protein of interest were again concentrated using centrifugal filters and stored in glycerol 33% (v/v) at −20 °C until used in experiments. Protein concentrations were measured by spectrophotometry at 280 nm (NanoDrop 1000, Thermo Scientific, DE, USA). For each construct, final concentrations typically ranged from 100 to 500 µM.

#### Baculovirus generation

The Izumo1 and Juno genes were transposed into bacmids by the transformation of pFastBac1 parent vectors into *E. coli* DH10Bac cells and plating onto 5-Bromo-4-chloro-3-indolyl β-D-galactopyranoside (X-gal) selection plates (Luria-Bertani agar with 40 µg/mL X-gal, 0.5 mM IPTG, 30 µg/mL kanamycin, 30 µg/mL tetracycline, 30 µg/mL gentamicin, pH 7.4). Bacmids were isolated from white colonies using a generic isopropanol extraction protocol for DNA. To confirm the identity of each bacmid, the transposed regions underwent PCR amplification using standard M13 sequencing primers followed by sanger sequencing. GenJet™ (Thermo Fisher Scientific, Massachusetts, United States) DNA In Vitro Transfection Reagent were then used to transfect ExpiSF™ Sf9 cells (Thermo Fischer Scientific, Massachusetts, United States, catalogue #A35243) with the purified bacmids as per the manufacturer’s instructions. P0 virus stocks were harvested by centrifugation and passing of the supernatant through a 0.45 µM filter. P1 and P2 virus stocks were generated by infecting ExpiSF™ cells 1 × 10^6^ cells/mL (Countess^TM^ 3 automated cell counter, Thermo Fischer Scientific, Massachusetts, United States) with the previous generation of viral stock for 48 h at 27 °C after which the viral stocks were harvested as described above. Finally, the identity of recombinantly expressed Izumo1 and Juno in P2 viral stocks was verified using the C-terminal SpyTags, as described previously^49^. Specific, covalent SpyTag/Catcher conjugation results in a large molecular weight shift that can be readily identified via SDS-PAGE. These sequence-specific conjugation events readily occurred at room temperate in PBS over a 1 h incubation.

#### Insect cell protein expression and purification

ExpiSF™ cells were cultured in ExpiSF™ chemically defined medium (Thermo Fischer Scientific, Massachusetts, United States) at 27 °C with shaking. As directed by the manufacturer, cultures were seeded in fresh medium at a concentration of 5 × 10^6^ cells/mL and mixed with ExpiSf^TM^ Enhancer (Thermo Fischer Scientific, Massachusetts, United States) 18 h before transfection. Transfection with P2-derived viral stocks was monitored via GFP fluorescence and increased cell size 24 h post-infection. Successfully transfected cultures were then incubated at 27 °C, with shaking, for a further 48–72 h or until cell viability dropped below 70%. ExpiSF^TM^ cells were removed via centrifugation and the culture medium passed through a 0.45 μM filter. Fastback Ni Advance Resin (Protein Ark, Cambridge, United Kingdom) was added to the clarified media and recombinant proteins were eluted into tris-buffered saline with glycerol (TBS-G, 20 mM Tris-HCl, 300 mM NaCl, and 10% glycerol (w/v), pH 8.0), supplemented with 0.1% (w/v) tween, using imidazole gradients across gravity-flow columns. Fractions containing the recombinant proteins were further purified using SEC into TBS-G (20 mM Tris-HCl, 300 mM NaCl, and 10% glycerol (w/v), pH 8.0) storage buffer and concentrated using centrifugal concentrators. Concentrated proteins were processed with detergent removal spin columns (Pierce®, Thermo Fischer Scientific, Massachusetts, United States) and stored at −20 °C. Directly prior to use in experiments, Izumo1-His_10_-SpyTag-LPETGG and Juno-His_10_-SpyTag-LPETGG aliquots were incubated over night at 4 °C with equimolar amounts of ybbR-His_6_-ELP-(MV7E2)_3_-ddFLN4-SpyCatcher or ybbR-His_6_-ELP-(MV7E2)_3_-SpyCatcher as required. Protein concentrations were measured by spectrophotometry at 280 nm. For each construct, final concentrations typically ranged from 30 to 150 µM.

### Atomic force microscopy single-molecule force spectroscopy

#### AFM sample preparation

Site-specific immobilization of samples for atomic force microscopy-based single-molecule force spectroscopy (AFM-SMFS) was performed as described previously^50,51^. In brief, AFM cantilevers (Biolever Mini AC40TS, Olympus, Tokyo, Japan) were treated with an ultraviolet ozone cleaner (Novascan, Iowa, United States) for 40 min and 25 mm diameter round cover glass surfaces (Menzel Gläser, Braunschweig, Germany) were incubated in piranha solution (1:1 (v/v) 30% H_2_O_2_: concentrated H_2_SO_4_) for 30 min. Irradiated cantilevers and piranha etched cover glasses were then incubated with 50% (v/v) (3-Aminopropyl)dimethylethoxysilane (ABCR, Karlsruhe, Germany), diluted in isopropanol, with water then added to 0.5% (v/v), for 5 min and 30 min, respectively. Silanated cantilevers were then washed successively in toluene, isopropanol, and water. Silanated cover glasses were thoroughly washed in water three times. The silanized cantilevers and cover glasses were cured at 60 °C for 40 min. Next, cantilevers and cover glasses were incubated in a solution containing 20 mg/mL of sulfosuccinimidyl 4-(N-maleimidomethyl)cyclohexane-1-carboxylate (Thermo Fisher Scientific, Massachusetts, United States) in 4-(2-Hydroxyethyl)piperazine-1-ethanesulfonic acid buffer (HEPES, 50 mM HEPES, 150 mM NaCl, pH 7.5) for 1 h. The exposed maleimide groups on the cantilevers and cover glasses were reacted with 200 μM coenzyme A in Mal-Coupling buffer (50 mM Na₂HPO₄, 50 mM NaCl, 10 mM C₁0H₁6 N₂O₈, pH 7.2) for 2 h at room temperature. In the final modification step, the proteins of interest (i.e., Izumo1/Juno with covalently attached ybbR-tags present on AFM-handles) were incubated with cantilevers and cover glasses with 10 mM MgCl_2_ and 1.5 μM SFP in PBS buffer for 4 h at room temperature. Between each modification step, following silanization and prior to the functionalization of proteins, cantilevers and cover glasses were washed three times with water. Following the functionalization of proteins, the cantilevers and cover glasses were thoroughly washed with PBS (pH 7.4).

#### AFM-SMFS measurements, constant speed

Constant speed SMFS measurements were performed on a ForceRobot® AFM (JPK instruments, Berlin, Germany). The contact-free method was used to calibrate cantilever spring constants, ranging from 0.06 to 0.14 N·m^−1^. The cantilever was brought into contact with the surface at a force setpoint of 0.18 nN for a period of 0.2 s and subsequently retracted at constant speeds of 800, 1600, 3200, and 6400 nm·s^−1^. Between measurements, the glass surface was moved horizontally by 100 nm. In the absence of surface-cantilever interactions over an interval of 24 approach attempts, the glass surface was moved 1000 nm vertically. Typically, a 10–15 h experimental run yielded 15,000–20,000 measurements. The stringent surface functionalization procedure described above was optimized to ensure only 1–5% of force *vs*. extension traces show evidence of Izumo1:Juno interactions. Consequently, the vast majority of Izumo1:Juno interactions are single-molecule events and multiple-binding events are exceedingly rare. PBS, adjusted to a pH of 7.4, was utilized as the buffer solution for all AFM-SMFS measurements.

#### Constant speed SMFS data analysis and curve selection

AFM data was analyzed using a combination of Python, MATLAB (MathWorks), and R scripts.

Analysis and selection of single-molecule events followed transformation of baseline subtracted force *vs*. extension curves into contour length space. This transformation was performed by applying the assumptions of the freely rotating chain (FRC) model to each force *vs*. extension curve. The FRC model was selected over alternative models of force-extension behavior (e.g., the worm-like chain model) due to the broad range of Izumo1:Juno rupture events (20–700 pN). Further, the impact of bond-angle bending is expected to be minimal owing to the long ELP-linker lengths used in the design of the AFM-handles (~65 nm). In the FRC model, rigid freely rotating segments are assumed to be connected by a fixed angle. Here, the implementation given by Eq. (1) (corrected from ref. ^52^) was used to describe the stretching behavior for the polypeptide chains in this study, providing force *vs*. contour length curves.

1 $$\frac{x}{L}=\left\{\begin{array}{cc}\frac{{Fa}}{3{k}_{B}T}\hfill & {{\mbox{for}}} \; \frac{{Fb}}{{k}_{B}T} < \frac{b}{p}\hfill \\ 1-{\left(\frac{4{Fp}}{{k}_{B}T}\right)}^{-\frac{1}{2}} & {{\mbox{for}}} \; \frac{b}{p} < \frac{{Fb}}{{k}_{B}T} < \frac{p}{b}\\ 1-{\left(\frac{{cFb}}{{k}_{B}T}\right)}^{-1} & {{\mbox{for}}} \; \frac{p}{b} < \frac{{Fb}}{{k}_{B}T}\hfill \end{array}\right.$$

Where *x* is the extension, *L* is the contour length, *F* is force, *a* is the Kuhn length and is given by Eq. (2), *k*_*B*_ is the Boltzmann constant, *T* is the temperature, *b* is the bond length, *p* is persistence length and is given by Eq. (3), and *c* is a constant of 2.

2 $$a=b\frac{1+{\mathrm{cos\; \gamma }}}{(1-\cos \gamma )\cos (\gamma /2)}$$

Where *ϒ* is the fixed angle connecting bonds in the FRC model.

3 $$p=b\frac{\cos (\gamma /2)}{\left|{\mathrm{ln}}({\mathrm{cos\; \gamma }})\right|}$$

Following the transformation of force *vs*. extension curves into force *vs*. contour length curves, automated filtering and manual processing yielded ~70–150 force *vs*. contour length curves per pulling speed, per independent experiment. The automated filter was set to exclude curves showing forces and contour length increments indicative of non-specific cantilever-surface interactions, the absence of any cantilever-surface interaction, or the formation of multiple Izumo1:Juno complexes. Specifically, the automated filter excluded any force-extension curves that had sawtooth peaks within the first ~100 nm of extension, where no sawtooth peaks were observed, or more than 9 sawtooth peaks were observed. From the filtered dataset, single-molecule interactions were then manually identified using two different selection criteria. Under the first selection criteria, single-molecule interactions between Izumo1 and Juno were identified as individual rupture events <100 pN at contour lengths of ~135 nm. This criterion was based on FRC-fitting of the expected contour length the polypeptide chains for this force range and the expectation that above these forces the ddFLN4 fingerprint domains would typically unfold. The remaining curves were then sorted for individual rupture events that followed the unfolding of two ddFLN4 fingerprint domains. The unfolding of the two ddFLN4 domains contributed an additional 32 nm in contour length across four characteristic sawtooth-like peaks in the 50–80 pN range. This predictable unfolding pattern of ddFLN4, in addition to the ELP extension behavior, yields a final contour length of ~190 nm at rupture. The data sets were then further sorted based on the presence or absence of unfolding intermediates that could contribute on average an additional ~15–20 nm of contour length.

Next, rupture force distributions were fitted as GMM using the mclust package in R^53^. This approach allowed for the systematic statistical classification of the majority of unbinding events as either P0, P1, or P2. Here, the fitting parameters were set to allow for the best possible fit of the pooled rupture force distributions from each independent experiment, using one, two, three, or four Gaussians. To improve the robustness of the fitting approach across all of the tested variants, a square-root transformation was applied to the pooled rupture force distributions, which were then normalized prior to GMM fitting. In instances where a fourth Gaussian was detected in the GMM, they were limited to a single constant pulling speed for a non-wild type mutant and were largely overlapping with another Gaussian. Therefore, we could not attribute them to a distinct unbinding pathway. Instead they were attributed to minor experimental variance. Accordingly, where a fourth Gaussian was fitted in as part of the GMM, these rupture events were reclassified into the overlapping parent Gaussians. Nonetheless, we would like to highlight the possibility of future improvements to the resolution of AFM-SMFS, leading to the isolation of additional unbinding pathways in wild-type Izumo1:Juno. Such pathways would, of course, need to be accounted for in the fitting of future distributions.

To explore the theoretical energy landscape of Izumo1:Juno, the force-independent off-rate (*k*_0_), the distance to the transition state along the reaction coordinate (Eqs. (6–8) given as Δ*x*), and the force-independent free energy of activation (Δ*G*^‡^) for P0, P1, and P2 were first determined using the Dudko–Hummer-Szabo (DHS) model^38,39^. For this purpose, the rupture force distributions for each pathway across all constant pulling speed were combined and plotted as histograms with equal bin widths (Δ*F* = 10 pN). We would like to emphasize here that unlike the Bell-Evans (BE) model, the DHS model fits the full probability distribution of rupture forces rather than simply the median rupture forces and median loading rates for each pathway-pulling speed combination. Consequently, DHS model fits are influenced by the overall spread of rupture forces and low-probability rupture events at both higher and lower forces all contribute to the DHS fit, therefore, some deviation from the median loading rate and median rupture force coordinates can be expected.

After converting the rupture force histograms into binned rupture force probability distributions, the force-dependent off-rates were calculated using the histogram transformation method outlined in Eq. (4).

4 $${k}_{{\mbox{off}}}({F}_{k})=\frac{{h}_{k}r({F}_{k})}{(\frac{{h}_{k}}{2}+{\sum }_{i=k+1}^{N}{h}_{i})\Delta F}$$

Where *k*_off_(*F*_*k*_) is the force-dependent off-rate at the median rupture force of the *k*^th^ bin, *r*(*F*_*k*_) is the median loading rate of the *k*^th^ bin, and *h*_k_ is the height of the *k*th bin as calculated using Eq. (5).

5 $${h}_{k}=\frac{{C}_{k}}{{C}_{{\mbox{tot}}}\Delta F}$$

Where *C*_*k*_ is the number of counts in the *k*th bin and *C*_tot_ is the total number of counts in the histogram.

The resulting ln(*k*_off_(*F*_*k*_)) values were then plotted against the corresponding *r*(*F*_*k*_) and fitted using the DHS-model, outlined in Eq. (6). First, plausible boundaries for each parameter, based on prior empirical evidence and theoretical considerations, were specified. For all Izumo1:Juno variants, the ranges were as follows: *k*_0_ within [0.001, 20], Δ*x* within [0.001, 2], and Δ*G*^‡^ within [0.01, 20]. For each iteration of the model fitting, initial parameters for *k*_0_, Δ*x*, and Δ*G*^‡^ were randomly sampled from uniform distributions defined by the above-mentioned parameter boundaries (*n* = 10,000). Each set of initial parameter values then underwent optimization to minimize the residual sum of squares using the Levenberg-Marquardt nonlinear least squares algorithm available in base R. This fitting process was repeated for both the cusp-like (*υ* = 1/2) and cubic linear (*υ* = 1/3) DHS-parameters that describe the shape of the energy barrier. To ensure robustness of the DHS fitting procedure, the optimization process was subjected to multiple runs (*n* = 3) with different random seeds, and the consistency of the results was verified.

6 $${k}_{{\mbox{off}}}(F)={k}_{0}{\left(1-\frac{{vF}\Delta x}{\Delta {G}^{{\ddagger} }}\right)}^{\frac{1}{v}-1}{e}^{\beta \Delta {G}^{{\ddagger} }}\left[1-{\left(1-\frac{{vF}\Delta x}{\Delta {G}^{{\ddagger} }}\right)}^{\frac{1}{v}}\right]$$

Where *β*^-1^ = *k*_*B*_*T*, *k*_*B*_ is the Boltzmann constant, and *T* is the temperature. In addition, the shape of the energy barrier can be parameterized by choosing between either a cusp-like or a cubic linear shape during DHS-fitting.

To then apply the DHS-fitted parameters to the fitting of the force *vs*. ln(loading rate) graphs, Eq. (7) was used.

7 $$F=\frac{\Delta {G}^{{\ddagger} }}{v\Delta x}\left[1-{\left(\frac{1}{\Delta {G}^{{\ddagger} }}{{\mathrm{ln}}}\frac{{k}_{0}{e}^{-\beta \Delta {G}^{{\ddagger} }}}{\Delta {xl}}\right)}^{v}\right]$$

Where *F* is the rupture force and where *l* is loading rate.

The DHS fitting procedure can be summarized as follows:

Raw AFM rupture force + loading rate traces→Force histogram binning→Estimate *k*_off_ (*F*) via Eq. (4)→Plot ln(*k*_off_) *vs* Force→Fit DHS model using Eq. (6) to extract *k*_0_, Δ*x*, Δ*G*^‡^→Predict force–loading rate behavior using Eq. (7)→Predicted force–loading rate behavior plotted as dashed lines against experimental force–loading rate behavior.

For comparison to DHS-fitting, the Bell-Evans (BE) model^36,37^ was used to obtain *k*_0_ and Δ*x*, from fitting of the median rupture force against the logarithm of the median loading rate. In contrast to the DHS-model, in the BE model, Δ*x* is assumed to be independent of force.

8 $$F=\frac{{k}_{B}T}{\Delta x}{{\mathrm{ln}}}\left[\frac{{{\mathrm{r}}}_{f}\Delta x}{{k}_{0}{k}_{B}{{\mathrm{T}}}}\right]$$

Where *F* is the rupture force and *r*_*f*_ is the loading rate at rupture.

### AFM-SMFS measurements, force clamp

Force clamp SMFS measurements were performed on a ForceRobot® AFM. To measure the bond lifetime of protein complexes at different retraction force setpoints (20, 30, 40, 50, 60 70, 80, and 90 pN or 80, 90, 100, 110, 120, 130, 140, 150, 160, 170, and 180 pN), cantilevers were briefly brought into contact with cover glasses at a force setpoint <40 pN for a period of 0.2 s and then retracted at 4000 nm·s^−1^. Where the surface-cantilever interactions formed, the measured force increased as the cantilevers were retracted until the target force setpoint was reached. At the target force setpoint, the cantilever tip-sample distance was continuously adjusted to maintain the force setpoint until the complex ruptured. Measurements were limited to a maximum clamp time of 10 s. To assist in the correct selection of force *vs*. time curves, additional controls were also performed without Juno functionalized to the cover glass. PBS (pH 7.4) was utilized as the buffer solution for all measurements.

### Force clamp SMFS curve selection and data analysis

Over a typical 15 h measurement period, ~500–1000 force *vs*. time curves were collected for subsequent analysis (as previously, these represent ~1–5% of the total measurements). Following a combination of automated and manual processing of force clamp data, each experiment yielded ~10–60 force *vs*. time curves for each force setpoint. In the absence of fingerprint domains on the AFM constructs, an automated filter was written to exclude the signature of non-specific adhesion events identified from the non-Juno functionalized cover glass controls. These controls directly followed the Izumo1:Juno force clamp measurements, using the same cantilevers. In brief, the automated filtering excluded force *vs*. time curves with the following features as non-specific force clamping events: more than three discrete AFM head height steps, retraction distances less than 40 nm, retraction distances greater than 200 nm. Further, bond lifetimes less than 15 ms were excluded to avoid potential artifacts due to the regularization times of the feedback loop. The resulting plotted force *vs*. times curves were then inspected manually to ensure the absence of optical or mechanical artifacts and that the target force setpoint was properly maintained.

### Force clamp data analysis

In practice, the measured force setpoint on the ForceRobot® 300 AFM using BioLevever mini cantilevers was only accurate to within approximately 5 pN of the target setpoint. For the plotting and binning of force *vs*. time curves for subsequent statistical analysis, the force setpoint was identified as the highest mean force across any given ~5 ms window during bond clamping (as determined by the minimum bond lifetime cut-off limit). Lifetimes were calculated as the time at which the bond was clamped until the AFM resumed its retraction phase following bond rupture. Where the catch bond states of Izumo1:Juno represent the interplay of multiple unbinding pathways, we did not expect the assumption of normality to hold for all of the binned bond lifetimes within a force clamp experiment. To validate this assumption, the Shapiro–Wilk Test was used. Subsequently, the non-parametric Kruskal–Wallis test followed by Dunn’s multiple-comparison test was used for hypothesis testing of changes to bond lifetime within the binned lifetime distributions (10 pN). In all instances, *p-*values < 0.05 were considered statistically significant.

### Flow cytometry-based bead-binding assay

#### Polystyrene bead preparation

With the exception of the silanization step, site-specific immobilization of samples for bead-based flow cytometry binding assays was performed as described above for the AFM-SMFS cover glass preparation. For each binding assay, 30 million 3.75 µm amino-coated polystyrene breads (SPHERO™ Amino Polystyrene Particles, Sterotech, Illinois, United States). For each washing step outlined in the site-specific immobilization protocol, the beads were centrifuged at 14,000 × *g* for 3 min, removal of the supernatant, and then resuspended in required solution.

#### Flow cytometry binding assay

Flow cytometry binding assays were performed on an Attune NxT Flow Cytometer (Invitrogen, Massachusetts, United States). Izumo1-SpyTag fusions were labeled with SpyCatcher-GFP in 0.1% tween 20 (w/v) TBS-G, as described previously^54^, and detailed above. Starting at 100× the expected *K*_D_, 5-fold dilution series of Izumo1-GFP were prepared in 0.1% tween 20 (w/v) TBS-G, for a total of 7 dilutions. Approximately 1 μM Juno-SpyCatcher-ddFLN4-ELP-ybbR fusion was incubated with 30 million SpyCatcher-ddFLN4 functionalized polystyrene beads in TBS-G buffer. Each Izumo1-GFP dilution was then incubated with approximately 3.5 million Juno-conjugated polystyrene beads for 3 h before measurement. Directly before measurement, the polystyrene beads were washed in 0.1% tween 20 (w/v) TBS-G and resuspended to a final volume of 500 µL. For each concentration and biological replicate, 10,000 events were recorded and the measurements repeated in triplicate. The datasets were analyzed using R scripts and the Hill equation was fitted to extract the *K*_D_. Due to protein limitations, for the measurement of mouse Izumo1:Juno, the GFP-labeling and bead functionalization of Izumo1 and Juno, respectively, were reversed.

### Monte Carlo force spectroscopy simulations

Monte Carlo force spectroscopy simulations based on Kramer’s theory used to validate and explore the proposed multi-state catch bond model for Izumo1:Juno. Specifically, we sought to generate estimates for the kinetic parameters that govern the transition between each unbinding pathway, to test if reversible or non-reversible transitions were more likely, and to evaluate forces at which catch bond behavior might emerge. For these purposes, two different Monte Carlo force spectroscopy simulation approaches were conceived: one simulating constant speed AFM-SMFS and one simulating force clamp AFM-SMFS. These Monte Carlo force spectroscopy simulations and their applications described below generate synthetic AFM-SMFS datasets based on experimentally derived BE model parameters.

To briefly clarify the goal of generating estimates for the kinetic parameters that govern the transition between each unbinding pathway: unlike the kinetic parameters for P0, P1, and P2 unbinding, the energy barriers that govern Izumo1:Juno pathway switching cannot be directly observed or measured using constant speed AFM-SMFS. Instead, as described below, we performed a non-linear least square fitting of constant speed Monte Carlo force spectroscopy simulated rupture force histograms against the experimental rupture force histograms to evaluate and refine estimates of the pathway switching kinetic parameters (see below). Thus, the constant speed Monte Carlo force spectroscopy simulated rupture force histograms were generated using a combination of the experimental BE *k*_0_ and Δ*x* values for P0, P1, and P2 unbinding and randomly generated *k*_0_ and Δ*x* values for the unbinding pathway transitions. Between random generation and non-linear least squares optimization, millions of possible *k*_0_ and Δ*x* combinations were considered for each pathway transition.

#### Constant speed Monte Carlo force spectroscopy simulation

In the constant speed Monte Carlo force spectroscopy simulations, a worm-like chain model (WLC)^55^ was used to generate force values *F*(*t*_*i*_) across an evenly distributed molecular extension axis *X*(*t*_*i*_). The values on the molecular extension axis *X*(*t*_*i*_) were then converted to the equivalent AFM head height *H*(*t*_*i*_) using a bending correction (Eq. (9)).

9 $$H({t}_{i})=X({t}_{i})+\frac{F({t}_{i})}{k}$$

Together, the above steps allow for the correlation between the time of the simulation that has passed and the predicted forces acting on the simulated receptor:ligand complex. More specifically, it gives the WLC-predicted force at the current AFM head height *H*(*t*_i_), which is itself calculated based on the retraction speed of the AFM head and time of the simulation that has elapsed.

10 $${t}_{i+1}={t}_{i}+\frac{H({t}_{i+1})-H({t}_{i})}{V}$$

Next, using the relevant kinetic parameters (*k*_0_ and Δ*x*), the force-dependent off-rates were calculated for each of the unbinding pathways and the transition barriers that separate them using Eq. (11).

11 $${k}_{{\mbox{off}}}(F)={k}_{0}{e}^{\beta F\Delta x}$$

For the unbinding pathways P0, P1, and P2, the experimentally derived BE kinetic parameters (*k*_0_ and Δ*x*, see Supplementary Table 1) were used. We note here that the BE model kinetic parameters were chosen for the Monte Carlo constant speed and force clamp force spectroscopy simulations due to its numerical stability and computational efficiency when simulating force-dependent unbinding rates. In contrast, applying kinetic parameters derived from the DHS model in simulations can introduce numerical instabilities, sometimes resulting in unrealistically stable complexes at certain force regimes, making it unsuitable for large-scale iterative simulations. As referenced above, the energy barriers that govern transition between the unbinding pathways were assumed to behave in a manner consistent with the BE model. Accordingly, for each iteration of the Monte Carlo, the kinetic parameters for the transition between unbinding pathways were randomly generated within a defined range as follows: *k*_0_ within [0.00001, 100] and Δ*x* within [0.01, 10].

During constant speed Monte Carlo simulations, an array of time points (*t*) were generated and the force-dependent off-rate for each energy barrier at that time point was calculated as described above. These off-rates were then converted into the dissociation probabilities for all possible events, using Eq. (12).

12 $$P(F)=1-{e}^{-{k}_{{\mbox{off}}}(F)\Delta t}$$

At each time point in the simulation, the dissociation probability *P*(*F*) is compared to a random number between zero and unity. If the random number is smaller than *P*(*F*) the receptor:ligand unbinding or the unbinding pathway transition event occurs.

In the Monte Carlo simulations describing our kinetic models, the receptor:ligand complex always begins in the Native state (N). From N, there are two possibilities, unbinding through P0, or transition to intermediate state 1 (I_1_). From, I_1_ the simulated receptor:ligand complex can unbind through P1, transition to intermediate state 2 (I_2_), or depending on if reversible transition is allowed, return to N. from, I_2_ the simulated receptor:ligand complex can unbind through P2, or depending on if reversible transition is allowed, return to I_1_. At each time interval, all the possible events at the current state were randomly shuffled and the first event executed. If unbinding occurs, the corresponding force is recorded as the rupture force. If a transition state occurs, the simulation moves to the next time interval. To reflect the experimental results, constant speed Monte Carlo simulations repeated for 500 replicates for each constant pulling speed.

Using bin widths of 20 pN, the Monte Carlo rupture force distributions for each constant pulling speed were evaluated against the corresponding experimental rupture force distributions using the sum of squared residuals. Each set of initial parameter values then underwent optimization to minimize the residual sum of squares. This random numerical approach to fitting was then repeated 10,000 times. To ensure robustness and consistency, the optimization process was subjected to multiple runs with different random seeds. Across 100,000+ iterations for each batch of simulations, we retained the parameter set that resulted in the lowest sum of squared residuals, thus identifying the set of parameters that best represented the force-dependent kinetics for the switching between P0, P1, and P2.

#### Force clamp Monte Carlo simulation

In the force clamp Monte Carlo, an array of force setpoints were first generated. The parameter set from the constant speed Monte Carlo simulations that resulted in the lowest sum of squared residuals when compared to the experimental constant speed data histogram distributions was retained for the force clamp Monte Carlo simulations. For each force setpoint, the force-dependent off-rates were calculated for each unbinding pathway and transition barrier separating the pathways using Eq. (11). For each time interval, the force-dependent off-rate was multiplied by the total time elapsed to give the probability of bond rupture. From there, Monte Carlo simulations followed the same rules as the constant speed Monte Carlo simulations regarding starting state, transition between unbinding pathways, and complex rupture. If rupture occurs, the corresponding elapsed time was recorded as the bond lifetime. Simulations were repeated 100 times for each force setpoint. The relative frequency of simulated Izumo1:Juno complexes unbinding through each unbinding pathway at each force setpoint was calculated from the number of unbinding events that occurred through that pathway, divided by the total number of simulations performed for that set point.

#### Statistical analyses

The catch bond behavior of Izumo1:Juno is consistent with the presence of different unbinding pathways and intermediate states. As such, normality was not assumed, and nonparametric statistical tests were employed for all analyses of AFM-SMFS data. Furthermore, given the inherent low statistical power of SMFS measurements, *p*-values should be interpreted cautiously to avoid overinterpretation of the hypothesis testing. All below statistical tests were two-sided.

To assess whether rupture force distributions of different unbinding pathways vary significantly across different pulling speeds in constant speed AFM-SMFS, Kruskal–Wallis tests followed by pairwise Wilcoxon rank-sum tests were used. The null hypothesis (*H*₀) stated that the rupture force distributions for each pathway are identical across pulling speeds, while the alternative hypothesis (*H*₁) posited that they differ significantly (Kruskal–Wallis test, *p* < 0.05). Pairwise Wilcoxon rank-sum tests were subsequently used to identify specific unbinding pathway groups showing significant differences between pulling speeds (*p* < 0.05).

Similarly, to determine whether the emergence of Izumo1:Juno folded intermediate states in higher-order unbinding pathways (i.e., P1 or P2) was significant, Kruskal–Wallis tests followed by Dunn’s test (using Šidák correction). Here, *H*₀ assumed no difference in the frequency of intermediate state transitions between unbinding pathways, whereas *H*₁ suggested that the transition frequency is unbinding pathway-dependent (Kruskal–Wallis test, *p* < 0.05). Dunn tests then identified which unbinding pathway comparisons showed statistically significant differences in intermediate state occurrence (*p* < 0.05).

To analyze bond lifetimes across different force distribution bins, Kruskal–Wallis tests followed by Dunn’s multiple-comparison tests were used. Here, *H*₀ assumed that bond lifetime distributions are independent of the force setpoint bin, while *H*₁ predicted statistically significant differences in bond lifetime distributions existed between force setpoint bins (Kruskal–Wallis test, *p* < 0.05). Dunn’s multiple-comparison tests then identified the specific bin pairs exhibiting statistically significant differences (*p* < 0.05).

### Molecular dynamics simulations and analysis

#### Molecular dynamics simulations setup

The human Izumo1:Juno complex was modeled based on existing crystal structures (PDB codes: 5F4E, 5JKC, 5JKD, 5JKE;^9,43^) and validated as described previously^44^. Standardized protocols outlined below were used for Steered Molecular Dynamics (SMD) simulations, as they have proven successful in previous projects characterizing the detachment of bacterial adhesins that bind to Fibronectin^56^ and Vinculin^57^. Briefly, using the NAMD version 2.14^58^ software and CHARMM36 fixed-charge force field^59,60^, our systems were simulated under constant velocity pulling in an all-atom approach in explicit TIP3 water solution with periodic boundary conditions and a time step of 2 fs. Systems were neutralized and simulated in physiological salt concentration (150 mM NaCl) and NPT (number of particles, pressure, and temperature) ensemble with constant pressure (1 bar) and constant temperature (310 K) using Langevin dynamics. Protonation states were assigned at pH 7.4 using standard CHARMM36 topology definitions for titratable residues. A nonbonded cutoff of 12 Å was applied with a switching function starting at 10 Å, and long-range electrostatics were treated using the Particle-Mesh Ewald method. System minimization took place over 2000 steps, heating from 0 to 310 K over 145,000 steps, and equilibration (with backbone restraints of 2 kcal⋅mol^−1^⋅Å^−2^) over 1 ns.

To define the starting geometry of the Izumo1:Juno complex, we considered the orientation imposed by two factors: their respective membrane contact sites and the behavior of their gamete carriers in fertilization. Izumo1, a transmembrane protein on motile sperm, is represented in this study only by its extracellular region, which is crucial to initiate fertilization. The C-terminal residue P254, where Izumo1 traverses the sperm membrane, was chosen as the pulling point and the moving restraint with a force constant of 7 kcal mol^−1^⋅Å^−2^ was applied on it. Juno, located on the extracellular membrane of the egg, is attached via a GPI anchor at residue 228. This point was used as an anchor in simulations with a force constant of 2 kcal mol^−1^⋅Å^−2^. Constant velocity pulling was performed at 0.001 Å⋅ps^−1^ until full unbinding or 90 ns. This strategy allowed us to closely mimic the experimental conditions of the AFM-SMFS setup (Fig. 1c). Despite requiring pulling speeds several orders of magnitude higher, it has been demonstrated that SMD can recapitulate key features of mechanically induced unbinding and provide valuable insights into experimental results^25,26,56,57^. We simulated 50 independent trajectories for wild-type Izumo1:Juno, Izumo1:JunoH177Q, and Izumo1:JunoH177E. Mutations were prepared using the QwikMD^61^ plugin in VMD version 1.9.4a12^62,63^. All the simulations were run on the Piz Daint supercomputer from the Swiss National Supercomputing Center in Lugano, Switzerland (CSCS).

#### SMD data analysis and visualization

Trajectories were analyzed using previously published protocols^56,57^ and in house VMD/tool command line (TCL) and Python scripts based on the PyContact^64^ and MDAnalysis^65^ packages. Simulations were visualized in VMD^62,63^. Contacts between Izumo1 and Juno were calculated using the distance threshold of 4.5 Å (Supplementary Fig. 17). Rupture events were defined as frames where the total number of contacts between Izumo1 and Juno dropped to 0. Since multiple unbinding and rebinding events were observed along each Izumo1:Juno pulling trajectory, the peak force was defined as the single rupture event with the highest overall force. The force loading rate was then determined by linearly fitting the force *vs*. time data immediately prior (0.5%–2% of the total trajectory frames) to the rupture event with the peak force. Rebinding distance and rebinding time were measured between the first and the last time Izumo1:Juno lost all the contacts. Rebinding rate was calculated as the number of rupture events per ns. C-terminal to C-terminal distances for Izumo1:Juno complexes were measured between the center of mass of anchoring residue on Juno (S228) and pulling residue on Izumo1 (P254). Contact surface area was calculated in VMD^62,63^ using the TCL terminal for each trajectory at the initial production frame assuming it represents the unloaded state and at 2 ns, assuming it is in a loaded state (Supplementary Fig. 18). Relevant version and protien model information for SDM data analysis and visualization is provided in Table 1.

**Table 1:** Version numbers for TCL, PyContact, and MDAnalysis

Reagent or Resource	Sourse	Identifier
Protein structure	Protein Data Bank	5JKA, 5JKB, 5F4Q, 5JKC, 5JKE, 5JKD, 5F4E
Protein models	Vogel Lab	[10.1038/s41598-023-46835-0]
NAMD 2.14	Theoretical and Computational Biophysics Group, University of Illinois	[10.1002/jcc.20289]
VMD 1.9.4 (TCL)	Theoretical and Computational Biophysics Group, University of Illinois	[10.1016/0263-7855(96)00018-5]
MDAnalysis 2.0.0	MDanalysis.org	[10.1002/jcc.21787]
PyContact 1.0.4	Scheurer et al.	[10.1016/j.bpj.2017.12.003]

### Reporting summary

Further information on research design is available in the Nature Portfolio Reporting Summary linked to this article.

## Supplementary information

Supplementary Information

Movie 1

Movie 2

Movie 3

Movie 4

Movie 5

Reporting Summary

Transparent Peer Review file

## Source data

Source data

## Data Availability

The source data underlying Figs. 1d–h, 2d–g, 3b, d, 4e–g, and 5a–c; and Supplementary Figs. 1–11, 13b, 14d–h, 15d–h, 16d–h, 17d–h, and 18–23b–h are provided in the Source Data file. The MD simulation setup and input/final trajectory files are included within the Source Data file. The PDB files used in this study are available using the following accession codes: 5JKA5JKB5F4Q5JKC5JKE5JKD5F4E Source data are provided with this paper.

## Code availability

Code for performing the Monte Carlo simulations in Python is available at [https://github.com/NashLab/Monte-Carlo]. Source data are provided with this paper.
