# Supplementary material for: Multi-state catch bond formed in the Izumo1:Juno complex that initiates human fertilization
Source: Nat Commun. 2025 Aug 26;16:7952. doi: 10.1038/s41467-025-62427-0 (PMC12381214; doi:10.1038/s41467-025-62427-0)
Supplement: Supplementary file 1 — Supplementary Information [file 41467_2025_62427_MOESM1_ESM.pdf]

<u>SUPPLEMENTARY MATERIALS</u>

Force-dependent Reorganization and Mechanostability of the Izumo1:Juno Complex Involved in Mammalian Fertilization

Sean Boult[1,2,3‡], Paulina Pacak[3‡], Byeongseon Yang[2], Haipei Liu[2], Viola Vogel[3,4*], Michael A. Nash[1,2,4,5*]

[1] Department of Biosystems Science and Engineering, ETH Zürich; Basel, 4056, Switzerland
[2] Institute of Physical Chemistry, Department of Chemistry, University of Basel; Basel, 4058, Switzerland
[3] Department of Health Sciences and Technology, ETH Zürich; Zürich, 8092, Switzerland.
[4] National Center for Competence in Research (NCCR), Molecular Systems Engineering; Basel, 4058, Switzerland
[5] Swiss Nanoscience Institute; 4056, Basel, Switzerland
[‡] Equally contributing

*Correspondence to: viola.vogel@hest.ethz.ch or michael.nash@bsse.ethz.ch

Supplementary Text

Flow cytometry-based bead-binding assay controls

Juno-coated polystyrene beads were functionalized as per the described method. These beads were then incubated with GFP at the concentrations shown in Supplementary Fig. 1. For the GFP control we observed minimal non-specific binding at concentrations up to 20 μM. As an additional control, to ensure that the upper dynamic range for our flow cytometry-based bead-binding assay covered all variants of interest, the binding affinity for mouse Izumo1:Juno (mIzumo1:Juno) was also measured at 6.05 ± 0.15 μM (± SE) (Supplementary Fig. 1). This result is several-fold above the highest expected binding affinity for the human Izumo1:Juno (hIzumo1:Juno) mutants and within 2-fold of the previously reported surface plasmon resonance (SPR) measured value of mIzumo1:Juno, 12.3 ± 0.2 μM[1]. As with the experimental measurements, both controls were completed for two biological replicates. In combination with the accuracy of the wild-type hIzumo1:Juno complex in our flow cytometry-based bead-binding assay, relative to SPR[2], which provides information on the lower dynamic range of our assay, we are confident in the accuracy of the equilibrium binding behavior described for the surface functionalized Izumo1:Juno variants used in this study.

Confirming the absence of intermediate states for P0

We sought to verify that the near complete absence of intermediate states in P0 was not simply the result of bias during force *vs.* extension curve selection or classification. A specific scenario where this might be problematic is the exclusion of two concurrent P0 unbinding events from an intermediate followed by P0 unbinding. In both instances you would expect to see two peaks at ~20-60 pN. To address this concern, we plotted the force distributions relating to the initial appearance of the intermediates in P1 and P2 for each constant pulling speed (Supplementary Fig. 4b,d). Here, we see that the force distributions for the initial appearance of the intermediate states do not overlap with the rupture force distribution for P0 shown in Fig. 1f. Thus, we conclude that it is more likely that the contour length increments measured relate to a specific conformational change linked to, or following, the state changes that result in P1 or P2 unbinding.

On possibility of Izumo1 Ig-like domain unfolding contributing to intermediate state formation

A more conventional explanation for the observation of intermediate states during Izumo1:Juno unbinding is the unfolding of Izumo1 residues located outside of the interconnected disulfide network. Such a scenario would be largely limited to the partial unfolding of the Izumo1 Ig-like domain (Supplementary Fig. 4e). While the unfolding of Izumo1 residues 166 to 181 and 234 to 255 would contribute a contour length increment of 13.1 nm (15 aa × 0.365 nm = 5.5 nm and 21 aa × 0.365 nm = 7.7 nm, respectively), we see no evidence of the Ig-like domain unfolding prior to rupture in the SMD simulations (Supplementary Movie 1, Supplementary Movie 2). Furthermore, across the different experimental constant pulling speeds, we often observe secondary peaks in the plotted contour length distributions (Supplementary Fig. 4). The ~25-40 nm final contour lengths of these secondary peaks are best explained by some combination of rare independent unfolding events (*i.e.* the unfolding of the Ig-like domain) in addition to the force-dependent re-organization of the Izumo1:Juno interface. The possibility of Izumo1 unfolding is further discussed in relation to Supplementary

Text sections for the constant speed AFM-SMFS of the Izumo1 point mutations, in particular for Izumo1K150A:Juno and Izumo1R160A:Juno.

An important consideration for any scenario involving fertilization is that any unfolding of Izumo1 or Juno that increases the membrane-to-membrane distance > 10 nm would actively inhibit membrane fusion[3]. Thus, unfolding of the Ig-like domain at the experimentally observed rates for the appearance of intermediate states during Izumo1:Juno unbinding would seem counterintuitive to sperm-egg cell fusion. We do not believe this assumption holds true in the force-dependent reorganization of the Izumo1:Juno interface purported by our SMD simulations. Here, Juno acts like a fulcrum to drive the N-terminal fusion associated residues of Izumo1 (F28, W88, and W113[4]) towards the egg cell membrane (Supplementary Fig. 21). Additionally, contact between Izumo1 and the egg-cell membrane would alleviate tension from the C-terminal Juno α-helix, prevents unfolding. Therefore, the increased distance between the C-termini of Izumo1 and Juno in our unbinding model would not necessarily inhibit membrane fusion in the same way that Ig-like domain unfolding might. Instead, the observed intermediate states may in fact represent key molecular events in overcoming the energetic barriers of membrane fusion. As discussed in the main text, such an unbinding mechanism also provides a possible explanation for the unexpectedly low rates of Izumo1 fusogenic activity observed under equilibrium conditions[4].

<u>Rationale for the selection of Izumo1 mutants based on sequence conservation, equilibrium binding behavior, and SMD simulations</u>

Izumo1E71A. Izumo1 E71 is a divergent residue that forms a salt bridge with Juno K163 at the equilibrium binding interface[2] (Supplementary Fig. 5b). An alanine substitution at Izumo1 E71, forming the complex Izumo1E71A:Juno, has previously been shown to have minimal impact on the equilibrium binding behavior[2]. Similarly, we observed a K_D for Izumo1E71A:Juno of 43 ± 5 nM (± SE) (Supplementary Fig. 1). To reiterate, in our wild-type Izumo1:Juno SMD simulations, when force is applied to through the pulling of the C-termini residues at a constant speed, Cluster 1 unbinds first (Fig. 2g,h). The resulting second-state Izumo1:Juno interface then consists primarily of Cluster 2 amino acid residues, which includes Izumo1 E71. During unbinding from the second state, Izumo E71 typically forms one of the terminal interactions with Juno (Supplementary Fig. 5b; Supplementary Movie 1). Stochastically, the Cluster 2 amino acids stabilize Izumo1:Juno long enough for the Cluster 3 interactions to form, thereby transitioning the system to a third state and therefore the three-state unbinding pathway (Supplementary Fig. 5c; Supplementary Movie 2). Once in the three-state unbinding pathway, Izumo1 E71 is removed from any interaction with Juno. Based on the analysis of these SMD simulations we reasoned that where Izumo1 E71A has minimal impact on the K_D, Izumo1E71A might impact the mechanostability of P1 and, or, the P1 → P2 transition rate. Based on this analysis and to further build upon previous characterization efforts of Izumo1E71A, we selected Izumo1E71A as one of the divergent Cluster 2 mutations used in this study.

Izumo1K150A. Izumo1 K150 is a divergent residue that forms a hydrogen bond with Juno C79 at the equilibrium binding interface[2] (Supplementary Fig. 6b). Previously uncharacterized, our equilibrium binding affinity experiments in Supplementary Fig. 1 demonstrates a modest decrease in K_D for Izumo1K150A:Juno to 110 ± 27 nM (± SE). At equilibrium, Izumo1 K150 represents the N-terminal-most Cluster 2 amino acid that is involved in the initial binding interface. With the application of force, additional contacts are made that extend the binding

interface beyond Izumo1 K150, thereby increasing the contact surface area (Supplementary Fig. 2e; Supplementary Fig. 6b,c). This increase in contact surface area under load is linked to the catch bond behavior we report for Izumo1:Juno. Similar to Izumo E71, as the system transitions from the two-state to the three-state unbinding pathway, Izumo1 K150 forms one of the terminal interaction points with Juno. Stochastically, these terminal interactions in our SMD simulations seem to support the formation of the Cluster 3 contacts necessary for transition to the three-state pathway. Furthermore, whilst Izumo1 K150 is a divergent residue, it is part of a highly-conserved short two amino acid loop boarded by two different disulfide bonds[2]. Of note, one of these cysteines, Izumo1 C152, forms a disulfide bond with the N-terminal most residue of Izumo1, C22 (Supplementary Fig. 4e). We therefore had to consider the possibility that a mutation in this region could alter the disulfide network and cause large intermediate unfolding events that might affect long term stability of AFM-SMFS measurements. Overall, we were interested in any possible effects of an alanine substitution Izumo1 K150 on the function of the short Izumo1 loop, in particular on the P0 → P1 and P1 → P2 transition rates.

Izumo1H157A. Izumo1 H157 is a highly conserved residue forming no clear non-covalent interactions with Juno in the crystal structure[2]. However, as previously shown[5], Izumo1 H157 forms a hydrogen with Juno R87 following equilibration in MD structures (Supplementary Fig. 7b). Experimentally, a single alanine substitution, forming the complex Izumo1H157A:Juno, has previously been shown to cause large changes to the equilibrium binding behavior of Izumo1:Juno[2]. In this study, we observed a K_D for Izumo1H157A:Juno of 243 ± 23 nM (± SE). As a Cluster 1 residue, we observed the involvement of Izumo1 H157 in the initial contacts between Izumo1:Juno before unbinding during the transition to the second-state in our SMD simulations (Supplementary Fig. 7b). Following, unbinding of Izumo1 H157 and transition to the second-state, Izumo1 H157 formed no additional interactions (Supplementary Fig. 7b,c). Therefore, we expected that any resulting changes to unbinding the behavior would be localized to the interaction between Izumo1 H157-Juno R87. Our rationale to select Izumo1 H157 was based on the high degree of sequence conservation across mammalian species[2] and as Cluster 1 with an established negative impact on equilibrium binding affinity[2]. From a mechanical perspective, we expected it to impact primarily the P0 → P1 transition rate.

Izumo1R160A. Izumo1 R160 is a conserved residue that forms hydrogen bonds with Juno E45 and Juno H65 in the equilibrium crystal structure[2] (Supplementary Fig. 8b). A single alanine substitution, forming the complex Izumo1R160A:Juno, has previously been shown to impact the equilibrium binding behavior of Izumo1:Juno[2]. Similarly, here, we observed a K_D for Izumo1R160A:Juno of 157 ± 14 nM (± SE) (Supplementary Fig. 1). A Cluster 2 residue, our SMD simulations predict that Izumo1R160 unbinds early following the transition to the second-state. Following this initial unbinding, Izumo1R160 forms no subsequent interactions and the system can evolve as either a two-or-three state unbinding trajectory, depending on if cluster the three amino acids form stable interactions (Supplementary Fig. 8b,c). Finally, as a Cluster 1 residue, Izumo1 R160 is located close to the Ig-like domain and it was hypothesized that disruption of the force-propagation networks in this area might provide additional insight into the origin of the contour length intermediates.

Analysis of fitted DHS and BE parameters for Izumo1 point mutations

We performed constant speed AFM-SMFS on the Izumo1 point mutations in triplicate, using the same experimental approach described for wild-type Izumo1:Juno. Similarly, to obtain insights into the theoretical energy landscapes for the unbinding pathways, we used the same force *vs.* extension curve sorting and model fitting approaches as wild-type Izumo1:Juno (Supplementary Table 2). Where broad agreements were observed in the DHS and BE derived k_0 values, they were limited to the increase in P2 k_0 for Izumo1E71A:Juno, the decrease in P1 k_0 for Izumo1K150A:Juno, and the decrease in P2 k_0 for Izumo1R160A:Juno (Supplementary Table 2). For the DHS and BE derived Δx values, agreement was found for decreased P0, increased P1, and decreased P2 in Izumo1E71A:Juno; and increased P0 and decreased P2 in Izumo1K150A:Juno (Supplementary Table 2). However, in no instances were these differences sufficient to result in more than subtle changes to mechanostability of the force activated pathways. Indeed, with the exception of P1 for Izumo1K150A:Juno, we see similar or greater median rupture forces for each of the Izumo1 mutants in P1 and P2 for all pulling speeds (Supplementary Figs. 5–8). Thus, as explained in the main text, it becomes clear that where alanine substitutions of single Izumo1 residues are sufficient to disrupt the equilibrium binding behavior of Izumo1:Juno (Supplementary Fig. 1)[2], the mechanical behavior of Izumo1:Juno offers a far greater level of functional redundancy. In the context of natural selection and the necessity of successful fertilization, this suggests that the mechanical stability of Izumo1:Juno is of greater import than the equilibrium binding affinity.

Analysis of unbinding pathway distribution and intermediate states for Izumo1 point mutations

Izumo1E71A:Juno. Interestingly, despite Izumo1 E71 forming terminal interactions during the two-state unbinding SMD simulations of the wild-type complex (Supplementary Fig. 5b); experimentally, we observed the ratio of P0 to P1 and P1 to P2 transitions increasing for Izumo1E71A:Juno (Supplementary Fig. 5d,e). This behavior, observed at all constant pulling speeds, suggests that the terminal interactions observed for Izumo1 E71 during SMD are not directly relevant for the mechanostablility of each unbinding pathway. Instead, we propose they regulate the formation of Cluster 3 interactions between Izumo1 and Juno (Supplementary Fig. 5b,c). Unique amongst the Izumo1:Juno variants used in this study, for Izumo1E71A:Juno we also observe the increased presence of long intermediate states in P0 at pulling speeds of 3200 and 6400 nms^{-1} (Supplementary Fig. 5f). Here, the median contour length changes of 30.1 ± 9.8 nm (± MAD) were observed. Given the established relationship between the higher order unbinding pathways and the observation of intermediate states in wild-type Izumo1:Juno, these results indicate the presence of unique intermediate states for Izumo1E71A:Juno in P0. We also see a tailing in the contour length distribution of P1 and P2 intermediates for Izumo1E71A:Juno that is a further indication that the additional intermediate states observed in P0 are also present in P1 and P2 (Supplementary Fig. 5g,h). However, the resulting median P1 (15.6 ± 7.2 nm, MAD) and P2 (16.2 ± 9.2 nm, MAD) contour length values remain similar to the wild-type Izumo1:Juno, suggesting that any such intermediates are of low abundance relative to the most prominent intermediate states. Overall, our constant speed AFM-SMFS experiments and SMD simulation implicate Izumo1 E71 primarily in the transition between the unbinding pathways.

Izumo1K150A:Juno. Like Izumo1E71A, Izumo1K150A is a Cluster 2 single alanine substitution**.** Unlike Izumo1E71A:Juno, the ratios for P0:P1:P2 in Izumo1K150A:Juno remain largely unchanged (Supplementary Fig. 6d,e). However, similar to Izumo1E71A:Juno, for Izumo1K150A:Juno the median P1 (20.7 ± 13.1 nm, MAD) and P2 (21.4 ± 15.5 nm, MAD) contour length values increase relative to the wild-type Izumo1:Juno at 3200 nms^{-1} (Supplementary Fig. 6g,h). We note that for Izumo1K150A:Juno, the AFM-SMFS measurements displayed poor stability, with cantilevers regularly losing activity after only 1-2 h. As such, only one replicate remained active for the entire measurement duration of 12 h and is shown in Supplementary Fig. 6. We posit that the increased contour lengths and broader distribution of the intermediate states for this mutant may indicate the presence of some additional states unique to Izumo1K150A:Juno (Supplementary Fig. 6g,h). It is possible that these additional intermediates impact long-term functionality following mechanical stresses. As previously discussed in the rationale for the selection of K150A as an Izumo1 mutant, this loss of mechanostability could be related to the bordering disulfide bonds (C22–C149). Disruption of this particular disulfide bond could lead to unfolding of parts of the Izumo1 alpha helical bundle (Supplementary Fig. 4e) and early failure of the cantilever tips through loss of Juno recognition.

Izumo1H157A:Juno. For the remaining Cluster 2 alanine substitution, Izumo1H157A, we observe inhibition of transition to the P2 pathway at all pulling speeds. Of particular note, only 7.9 ± 6.8% (SEM) of Izumo1H157A:Juno complexes unbind through P2 at 6400 nms^{-1} (Supplementary Fig. 7d,e). Furthermore, the median P0 and P1 rupture forces for Izumo1H157A:Juno are between 25.1 to 36.8 pN for loading rates of 1.2 to 14.6 nNs^{-1} and 89.2 to 144.9 pN for loading rates of 2.6 to 39.6 nNs^{-1}, respectively (Supplementary Fig. 7d). Thus, the possibility that the decrease in P2 unbinding is due to the premature rupture of Izumo1H157A:Juno can be excluded. Where the transition from P1 to P2 in Izumo1H157A:Juno is inhibited, a reverse transition to P0 increases in likelihood, thereby increasing the abundance of P0 at all pulling speeds except 800 nms^{-1}. Finally, for Izumo1H157A:Juno we observe no change to the relative rates for intermediate transition (Supplementary Fig. 7f) or the median contour lengths of the intermediate states for P1 or P2 (Supplementary Fig. 7g,h). Overall, it seems likely the conserved Cluster 2 residue Izumo1 H157 is involved in orienting the complex in the second-state for subsequent transition into the P2 unbinding pathway.

Izumo1R160A:Juno. Several interesting changes were observed for the Cluster 1 mutant Izumo1R160A (Supplementary Fig. 8). First, unique amongst all of the Izumo1:Juno variants tested, the GMM classifier identified a small proportion of Izumo1R160A:Juno complexes, 3.6 ± 2.8% (SEM), unbinding through P2 at pulling speeds of 800 nms^{-1} (Supplementary Fig. 8d,e). Yet, the percentage of P2 unbinding events for Izumo1R160A:Juno is lower for all other pulling speeds (Supplementary Fig. 8d,e). This effect is particularly evident at pulling speeds of 6400 nms^{-1} , where a ~20% reduction relative to wild-type Izumo1:Juno is observed (Supplementary Fig. 8d,e). Second, Izumo1R160A:Juno demonstrates an increase in P1 and P2 intermediate transitions events at all pulling speeds (Supplementary Fig. 8f). Third, we note that the median change in contour length following transition through these intermediate states increases for Izumo1R160A:Juno relative to wild-type Izumo1:Juno (Supplementary Fig. 8g). Here, the median contour length change is 22.4 ± 13.8 and 28.4 ± 14.9 nm (MAD), for P1 and P2 at 3200 nms^{-1}, respectively, nearly double that of the wild-type Izumo1:Juno. We predict that due

to the proximity of Izumo1 Cluster 1 residues to the Ig-like domain, the disruption of the force propagation pathway in Izumo1R160A:Juno (*i.e.* the hydrogen bonds between Izumo1 R160 and Juno E45 & H65) results in the unfolding of the Izumo1 Ig-like domain (amino acids 165–182 and 233–255, 17 + 22 × 0.365 = 14.2 nm) (Supplementary Fig. 8g). Given the possible sources of contour length increments for Izumo1 and Juno (shown in Supplementary Fig. 4e,f, respectively), the additional increases in contour length observed in Izumo1R160A:Juno support our initial conclusions from the wild-type Izumo1:Juno SMD simulations. Specifically, the contour length increments that are observed in wild-type Izumo1:Juno are largely the result of force induced reorientation of the Izumo1 and Juno binding interface rather than direct unfolding events.

In summary, regarding the presence of Izumo1:Juno folded intermediate states, our mutational analyses reveal changes to the Izumo1:Juno interface that result in distinct unbinding stages and contour lengths not observed in the wild-type complex. The reproducible nature of these unique contour length intervals across multiple measurements supports the interpretation that Izumo1:Juno folded intermediates, in general, correspond to discrete mechanistic states rather than artifacts arising from non-specific adhesion or multiple binding events. These findings further support the interpretation of the P1 and P2 pathways as functionally relevant, force-dependent intermediates in the Izumo1:Juno unbinding process.

Support for the reversibility of unbinding pathway transitions from constant speed AFM-SMFS

Despite the overall preservation of mechanostability for each unbinding pathway in the studied Izumo1 mutants, the alanine substitution of the well-conserved Cluster 2 residues had clear impacts on the P0, P1, and P2 unbinding ratios (Supplementary Figs. 5–8). This effect was particularly prevalent for the probability of unbinding through P2 at higher pulling speeds. At 6400 nms^{-1}, we observe a ~20% increase in P2 unbinding for Izumo1E71A:Juno and a ~20% decrease in P2 unbinding for Izumo1H157A:Juno (Supplementary Figs. 5 and 7). For reference, the well-conserved Cluster 1 mutant, Izumo1R160A:Juno, showed a ~10% decrease in P2 unbinding at 6400 nms^{-1} (Supplementary Fig. 8) and the divergent Cluster 2 mutant, Izumo1K150A:Juno, showed no change in P2 unbinding at 6400 nms^{-1} (Supplementary Fig. 6). This dichotomy of Cluster 2 alanine substitutions increasing or decreasing unbinding through higher order unbinding pathways, whilst subtly enhancing overall mechanostability in both instances, suggests the possibility of a dynamic equilibrium between the unbinding pathways. In particular it brings into focus a mechanistic question of whether the alanine substitutions are simply affecting the forward transition to higher order unbinding pathways (*i.e.* P0 $\rightarrow$ P1 $\rightarrow$ P2) or if reversible transitions are indeed possible on the experimental timescale (*i.e*. P0 $\rightleftharpoons$ P1 $\rightleftharpoons$ P2)[6]. In scenarios or mutants that favor the transition to higher order pathways or inhibit the transition back to lower order pathways, the mechanostability of the eventual unbinding pathway would be enhanced as less time is spent in lower mechanostability states[6]. Thus, we hypothesize that the small increases in mechanostability of Izumo1E71A:Juno and Izumo1H157A:Juno are the result of less time spent in lower order states during unbinding.

Unfolding events observed in SMD simulations

We observed two types of secondary structure adjustments across the SMD simulations. The first was the previously discussed unfurling of the C-terminal Juno α-helix, where the

occurrence increased from 4% in wild-type to 14% in both JunoH177Q and JunoH177E (Supplementary Fig. 19). The second major adjustment involved the stretching of the Izumo1 between residues E66 and V78, and subsequent unfurling of the α2-helix of Izumo1 (Supplementary Fig. 20). This structural change was most prevalent in the Izumo1:JunoH177Q and Izumo1:JunoH177E simulations, but was also observed to a lesser degree in the wild-type simulations.

Supplementary Figures

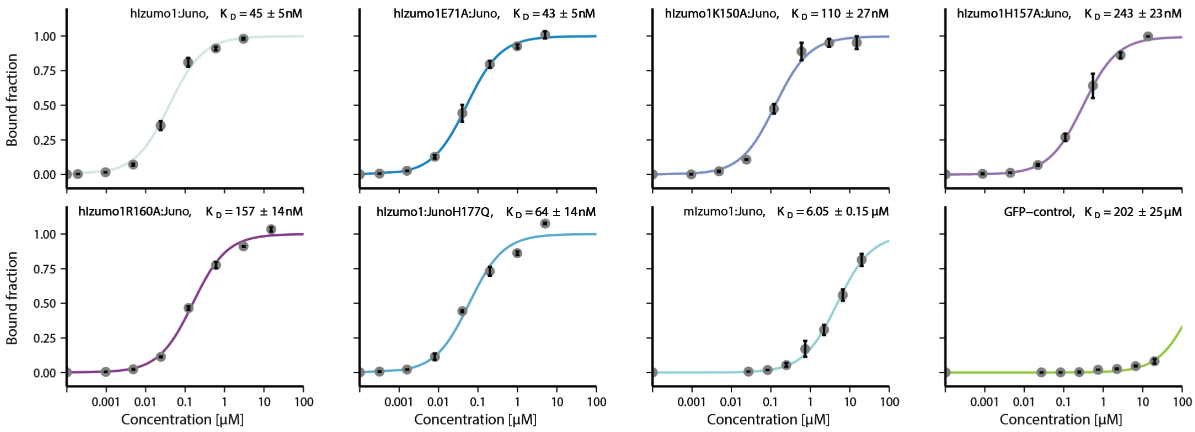

Supplementary Fig. 1: Flow cytometry-based bead-binding assay. Binding assays were performed for wild-type human (h) Izumo1:Juno, Izumo1E71A:Juno, Izumo1K150A, Izumo1H15A:Juno, Izumo1R160A:Juno, and Izumo1:JunoH177Q. Also included are mouse (m) Izumo1:Juno as an upper dynamic range control and GFP-only as a negative control. The bound fraction for each biological replicate represents the average of three measurements. Dots represent the average of two independent biological replicates. Error bars represent the range of the two independent biological replicates. Lines represent non-linear least squares fitting of the Hill equation with a single binding site. Binding affinities reported as K_D ± SE of the fitted parameters.

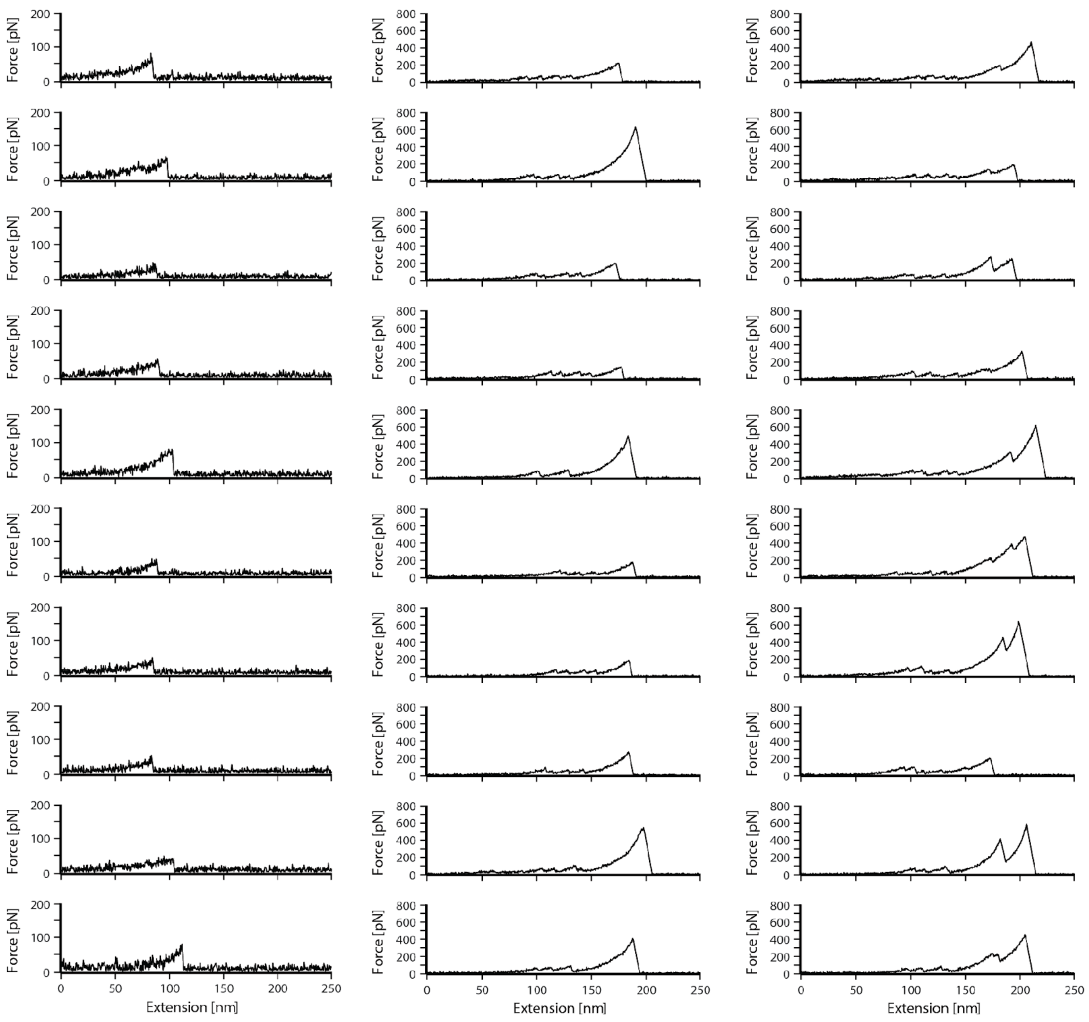

Supplementary Fig. 2: Representative constant speed SMFS force *vs.* extension curves from this study following automated and manual sorting. Each curve represents a distinct single Izumo1:Juno rupture event corresponding to each unbinding pathway subgroup a, b, or c. In the first column are force *vs.* extension curves showing Izumo1:Juno rupture at low force and in the absence of ddFLN4 fingerprint unbinding, classified as a. In the second column are force *vs.* extension curves showing Izumo1:Juno rupture following ddFLN4 fingerprint unfolding, classified as b. In the third column are force *vs.* extension curves showing Izumo1:Juno rupture following ddFLN4 fingerprint unfolding and an additional conformational rearrangement within the Izumo1:Juno complex, classified as c. Regardless of the subgroup (a, b, or c, which is determined by the number of unfolding peaks), each trace can be classified as P0, P1 or P2 depending on the rupture force.

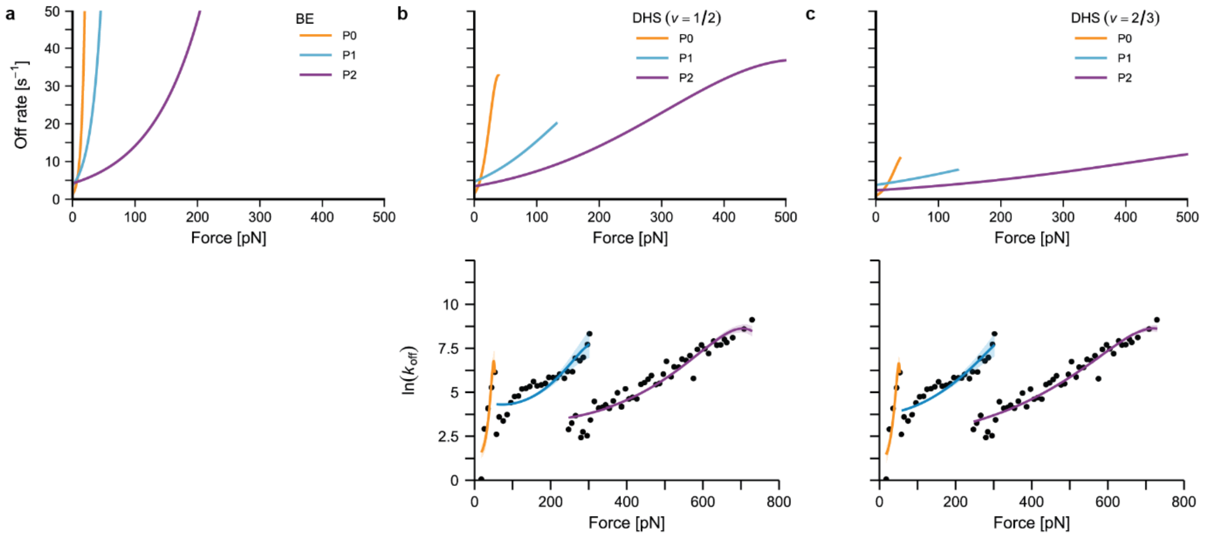

Supplementary Fig. 3: Force-dependent off-rates for Izumo1:Juno. Force-dependent off-rates for Izumo1:Juno calculated from the fitted Bell-Evans (BE) and Dudko-Hummer-Szabo (DHS) kinetic parameters shown below each respective plot. **a**, BE model fitted kinetic parameters were used to calculate force-dependent off rates for P0, P1, P2 (Eq. 11). **b**, Top: DHS model fitted kinetic parameters were used to calculate force-dependent off rates for P0, P1, P2 (Eq. 6), where v = 1/2. Bottom: ln(k_{off}) *vs*. force used in DHS model fitting to extract k_0, Δx, $\Delta G^{\ddagger}$. Lines show DHS model fits (Eq. 6). Ribbons show 95% confidence intervals of fits. **c**, Top: DHS model fitted kinetic parameters were used to calculate force-dependent off rates for P0, P1, P2 (Eq. 6), where v = 2/3. Bottom: ln(k_{off}) *vs*. force used in DHS model fitting to extract k_0, Δx, $\Delta G^{\ddagger}$. Lines show DHS model fits (Eq. 6). Ribbons show 95% confidence intervals of fits. All fitted kinetic parameters from this study are available in Supplementary Table 1.

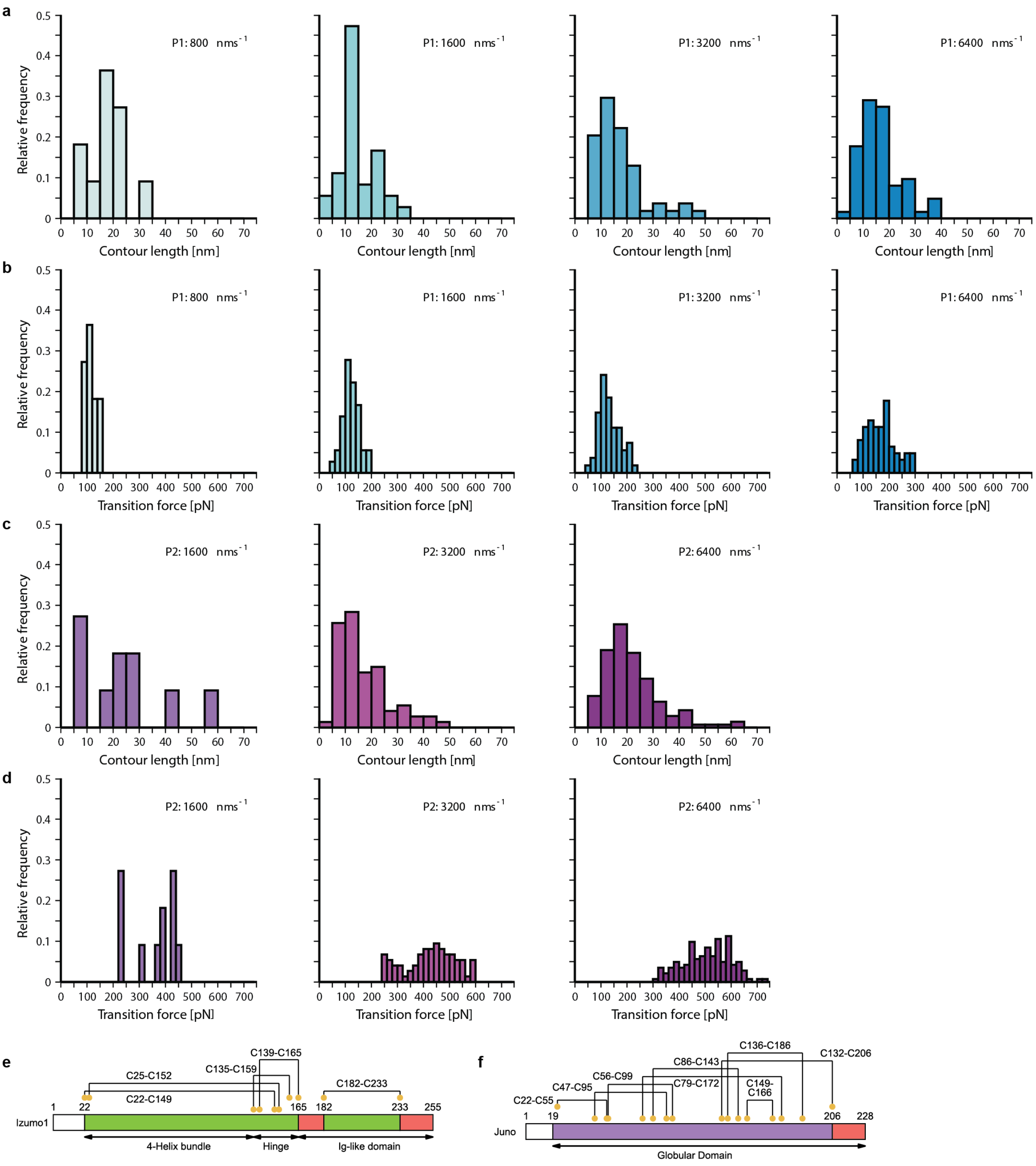
a
b
c
d
e
f
Relative frequency
Contour length [nm]
Transition force [pN]
P1: 800 nms-1
P1: 1600 nms-1
P1: 3200 nms-1
P1: 6400 nms-1
P2: 1600 nms-1
P2: 3200 nms-1
P2: 6400 nms-1
Izumo1
1
22
165
182
233
255
C25-C152
C22-C149
C135-C159
C139-C165
C182-C233
4-Helix bundle
Hinge
Ig-like domain
Juno
1
19
206
228
C22-C55
C47-C95
C56-C99
C86-C143
C79-C172
C136-C186
C149-C166
C132-C206
Globular Domain

Supplementary Fig. 4: Contour length distributions for Izumo1:Juno folded intermediate states. **a**, Distribution of contour length increments observed for Izumo1:Juno intermediate unfolding/rearrangement occurring prior to rupture through P1. **b**, Unfolding event force distributions for Izumo1:Juno rearrangements occurring prior to rupture through P1. **c**, Distribution of contour length increments for Izumo1:Juno rearrangements occurring prior to rupture through P2. **d**, Unfolding event force distributions for Izumo1:Juno rearrangements occurring prior to rupture through P2. Contour lengths show the total contour length change of Izumo1:Juno complexes following ddFLN4 unfolding until final Izumo1:Juno rupture. Transition forces show the force at which the first non-ddFLN4 contour length change occurs.). Data in **a**, **b**, **c**, **d** was drawn from four pooled independent experiments that were divided based on unbinding pathway for DHS model fitting For P1 800, 1600, 3200, and 6400 nms^{-1}, n = 10, 35, 53, 61. For P2 1600, 3200, and 6400 nms^{-1}, n = 9, 73, 145. **e**, Structural topology for Izumo1, including the conserved disulfide network. **f**, Structural topology for Juno, including the conserved disulfide network. For both Izumo1 and Juno topologies, red indicates regions available for mechanically induced unfolding. Ig-like = Immunoglobulin-like.

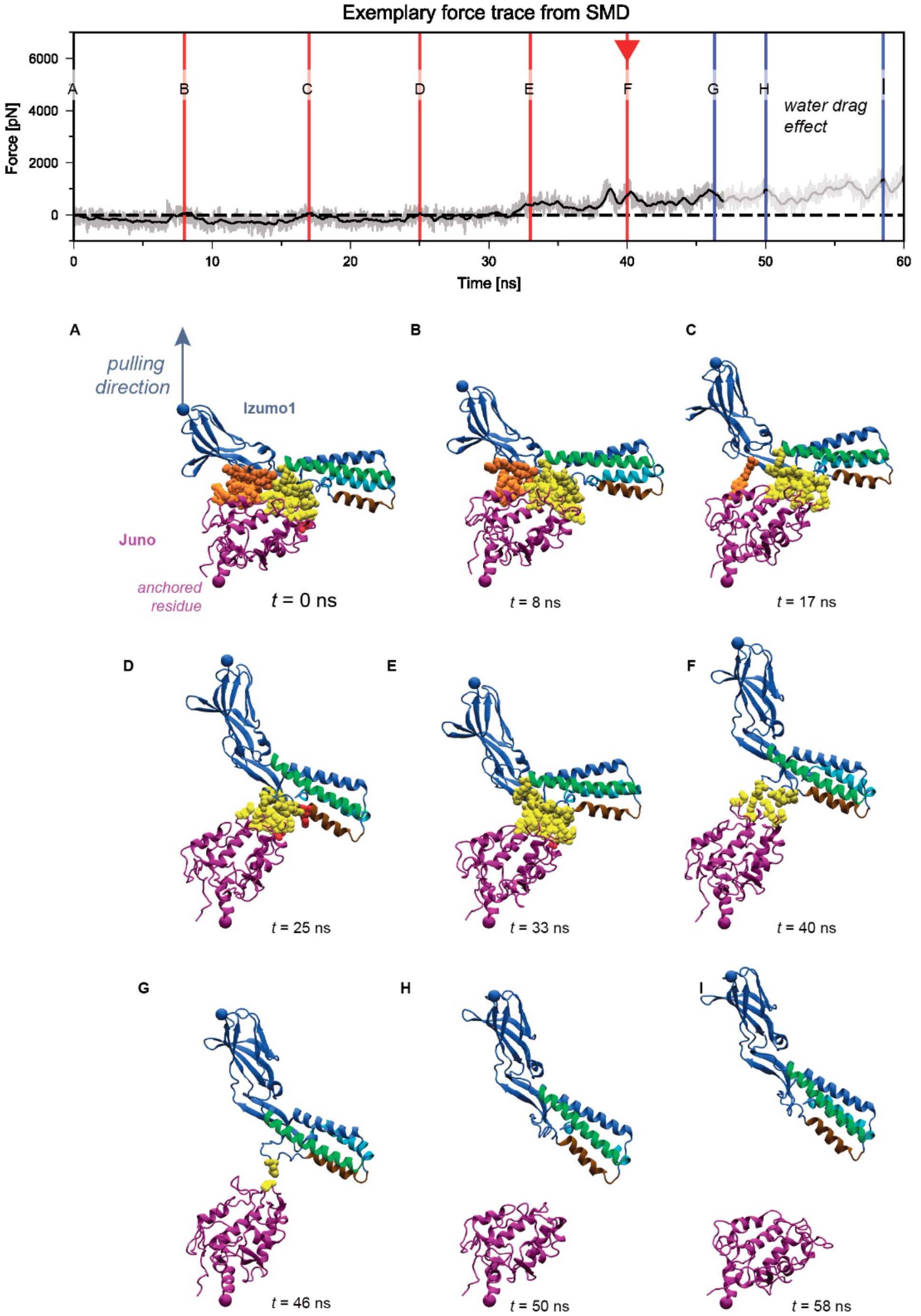

Supplementary Fig. 5: Force *vs.* time trace with structure snapshots representative of the two-state wild-type Izumo1:Juno unbinding observed in SMD simulations. Letters and lines mark timepoints at which snapshots were taken and the red triangle marks the first Izumo1:Juno rupture event. Gray traces represent force for individual frames and black lines are the rolling average force across the 50 frames. The number of contacts between Izumo1 and Juno as a function of time can be found in Supplementary Fig. 9a and the details of the corresponding interactions are shown in Supplementary Fig. 6. Preview of the unbinding process can be observed in Supplementary Movie 1.

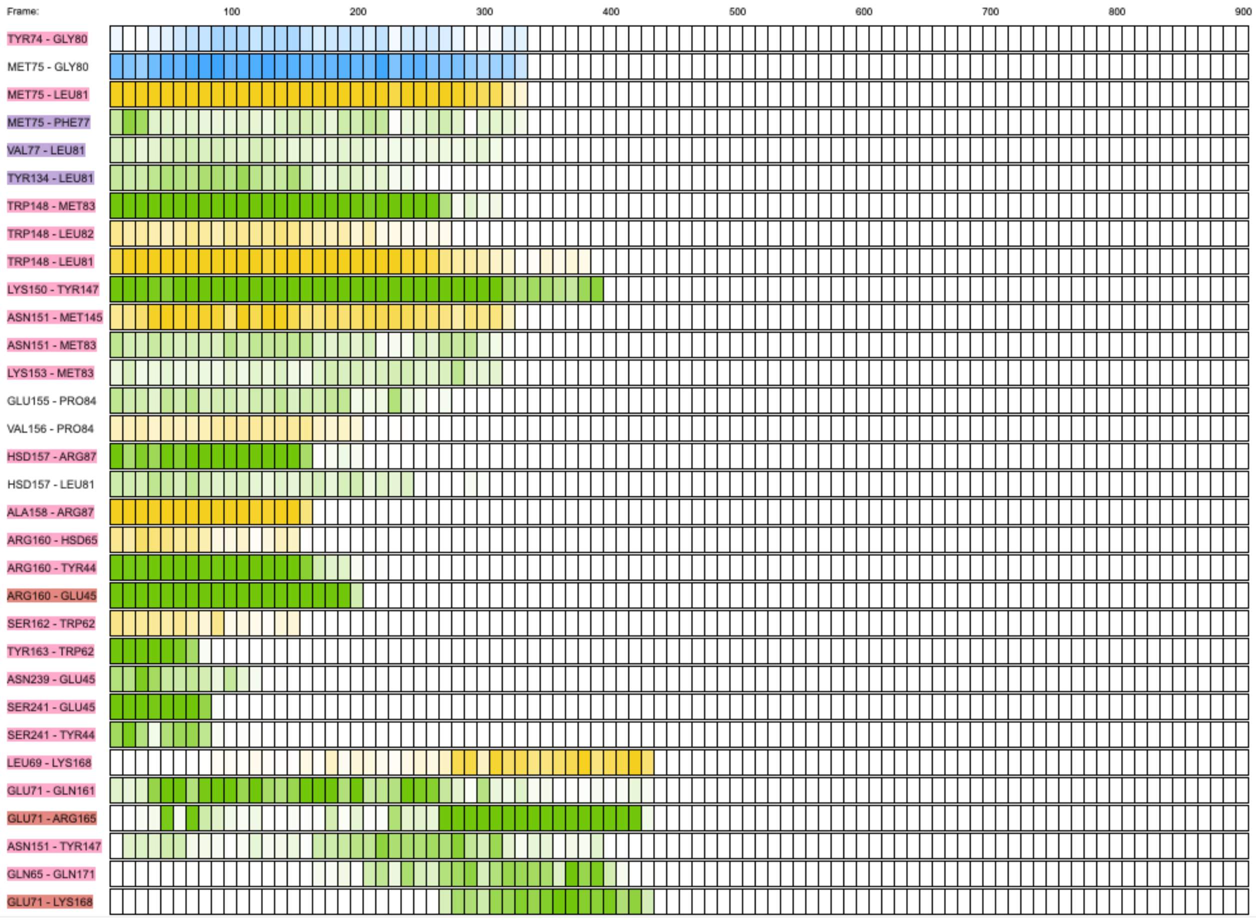

Supplementary Fig. 6: Exemplary time series of the non-covalent interactions as observed in the two-state unbinding pathway of the wild-type Izumo1:Juno complex in SMD simulations. Known binding interface of Izumo1:Juno complex gradually breaks within 50 ns, forming only few transient interactions in the process. Supplementary Movie 1 shows the preview of the corresponding simulation. The first residue in the label belongs to Izumo1, and the second residue belongs to Juno. The color of the label is determined by interaction type and geometry: red, salt bridge; blue, hydrophobic; and pink, hydrogen bond. Each box corresponds to the equal trajectory segment, their color signifies interaction type and the intensity shows the strength of the interaction: green, side chain/side chain; yellow, side chain/backbone; blue, backbone/backbone. PyContact[7] was used to extract this data with a filter of mean score value > 0.25 for trajectory of the total simulation time of 90 ns (900 frames extracted).

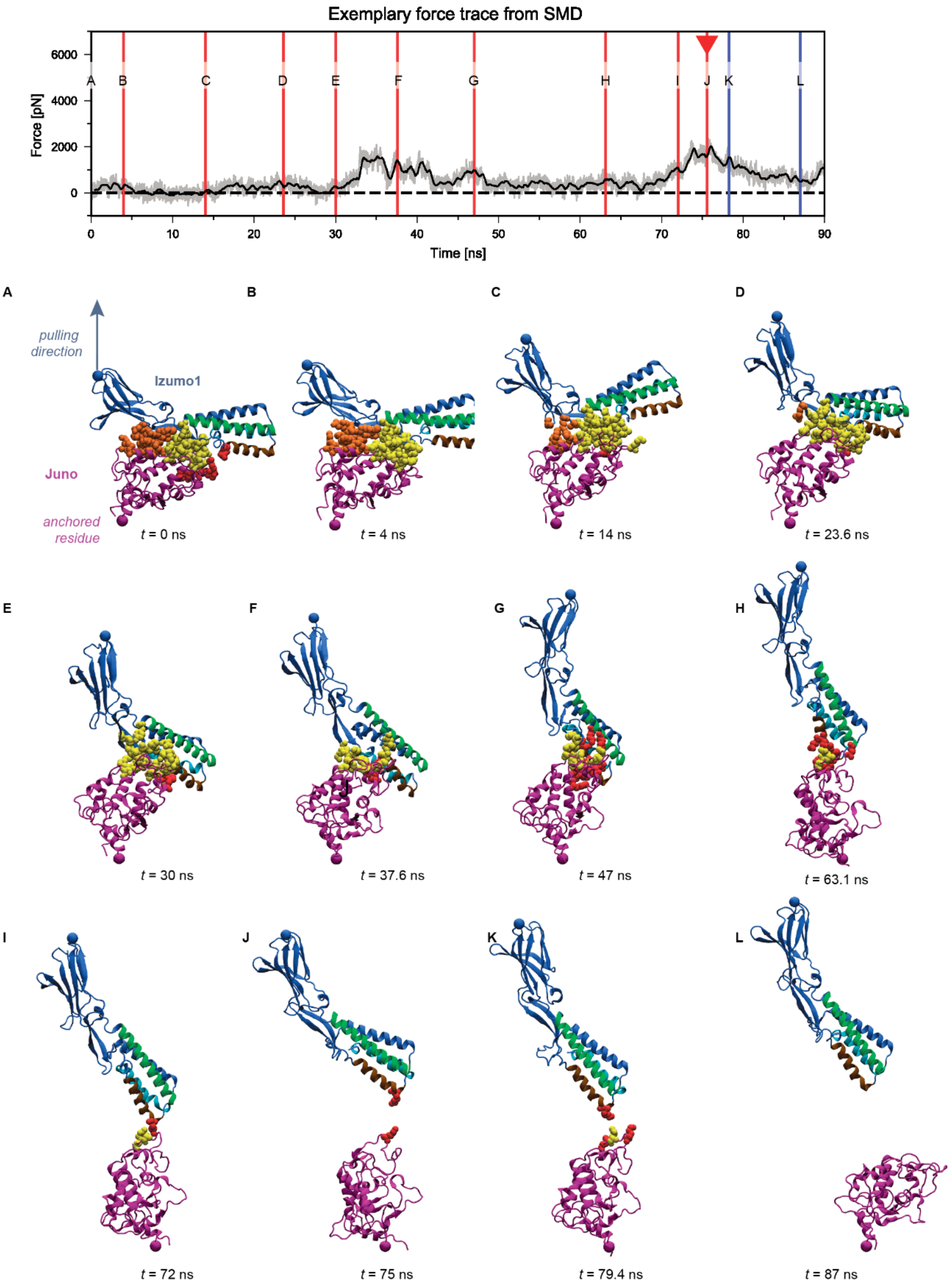

Exemplary force trace from SMD
Force [pN]
6000
4000
2000
0
0
10
20
30
40
50
60
70
80
90
Time [ns]
A
B
C
D
E
F
G
H
I
J
K
L
pulling direction
Izumo1
Juno
anchored residue
t = 0 ns
t = 4 ns
t = 14 ns
t = 23.6 ns
t = 30 ns
t = 37.6 ns
t = 47 ns
t = 63.1 ns
t = 72 ns
t = 75 ns
t = 79.4 ns
t = 87 ns

Supplementary Fig. 7: Force *vs.* time trace with structure snapshots representative of the three-state wild-type Izumo1:Juno unbinding observed in SMD simulations. Letters and lines mark timepoints at which snapshots were taken and the red triangle marks the first Izumo1:Juno rupture event. Gray traces represent force for individual frames and black lines are the rolling average force across the 50 frames. The number of contacts between Izumo1 and Juno as a function of time can be found in Supplementary Fig. S9b and the details of the corresponding interactions are shown in Supplementary Fig. 8. Preview of the unbinding process can be observed in Supplementary Movie 2.

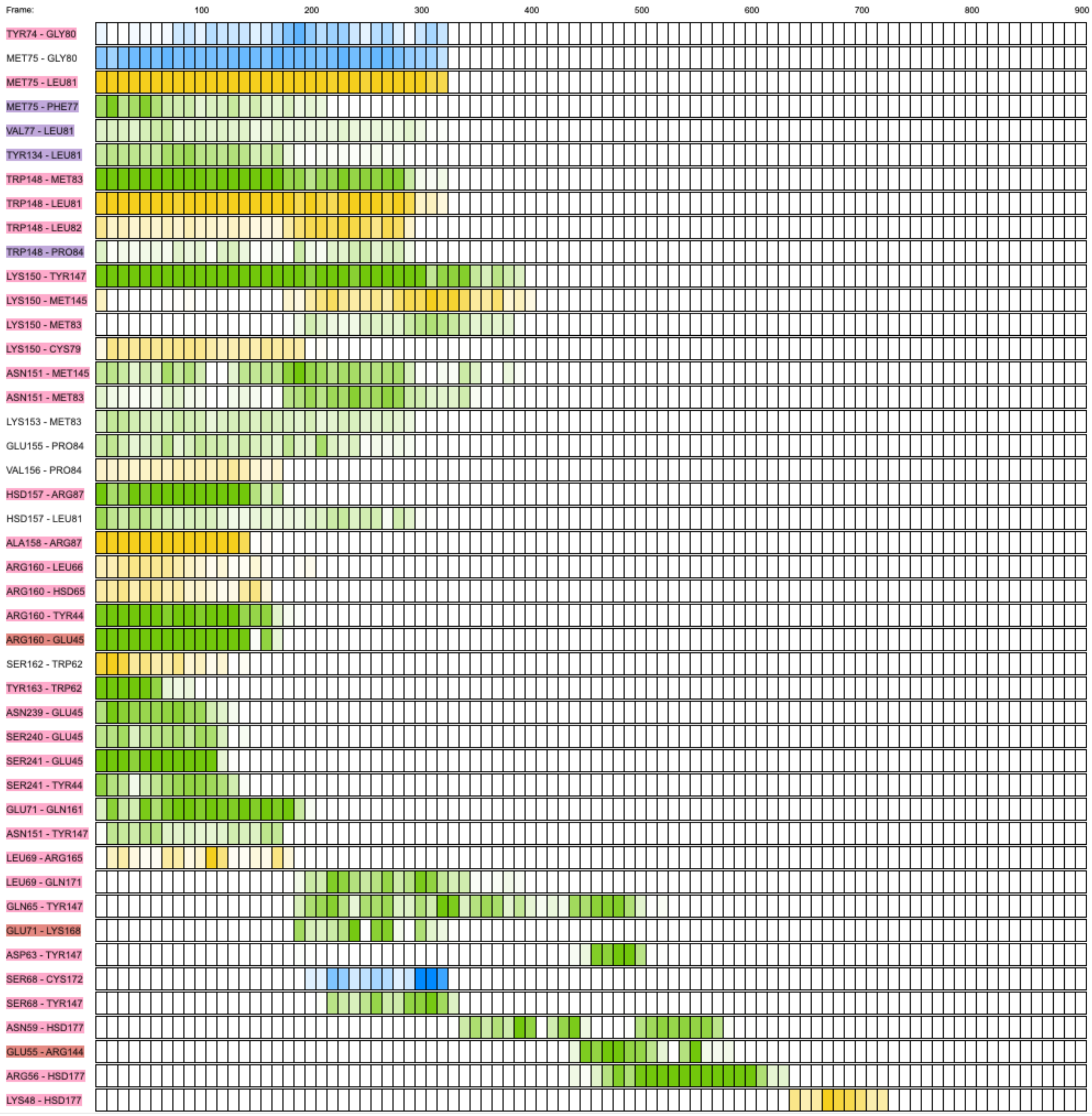

Supplementary Fig. 8: Exemplary time series of the non-covalent interactions as observed in the three-state unbinding pathway of the wild-type Izumo1:Juno complex in SMD simulations. In this trajectory the strongest late stage interactions were mediated by H177 of Juno forming three hydrogen bonds with N59, R56 and K48 of Izumo1**.** Total unbinding time was prolonged to over 70 ns. The strongest late stage interactions are mediated by H177 of Juno. Supplementary Movie 2 shows the preview of the corresponding simulation. The first residue in the label belongs to Izumo1, and the second residue belongs to Juno. The color of the label is determined by interaction type and geometry: red, salt bridge; blue, hydrophobic; and pink, hydrogen bond. Each box corresponds to the equal trajectory segment, their color signifies interaction type and the intensity shows the strength of the interaction: green, side chain/side chain; yellow, side chain/backbone; blue, backbone/backbone. PyContact[7] was used to extract this data with a filter of mean score value > 0.25 for trajectory of the total simulation time of 90 ns (900 frames extracted).

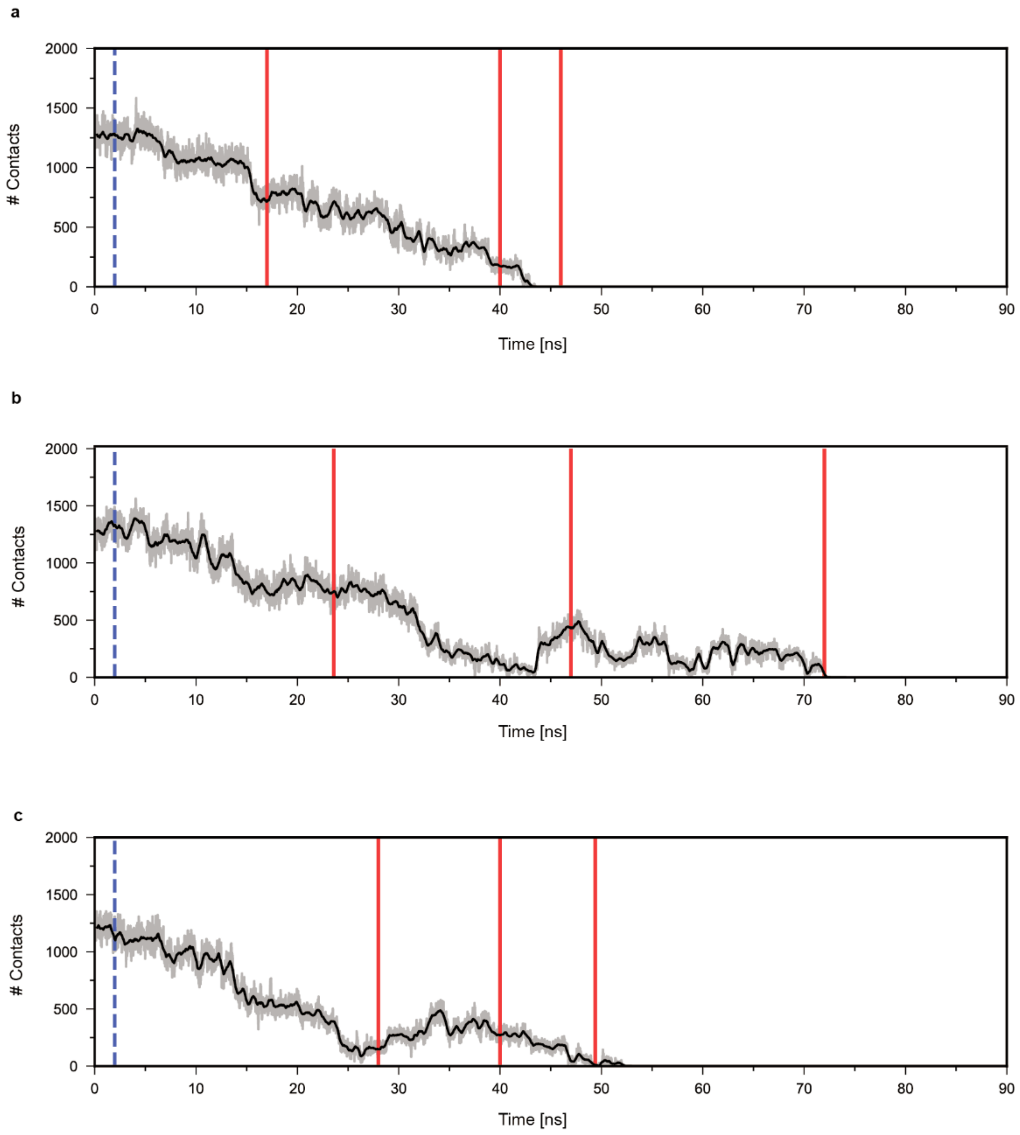

Supplementary Fig. 9: Number of contacts between Izumo1 and Juno traced as a function of time for representative unbinding trajectories of each type. a, During two-state unbinding, the number of contacts between Izumo1 and Juno steadily decreases over time. **b**, During three-state unbinding, the number of contacts increases during the first 2 ns. This is followed by an extended transition phase from ~15-30 ns, during which the number of contacts between Izumo1 and Juno plateaus at ~750. During the detachment of the second binding site at ~43 ns, the rotation of Izumo1 and Juno allows new contacts amongst residues that were previously unconnected. **c**, Number of contacts for the three-state unbinding subtype is consistent with the number of contacts during three-state unbinding shown in panel **b**. While the initial binding interface is lost earlier at ~25 ns, new contacts are formed and increase from 0 to ~500. Gray traces represent number of contacts for individual frames and black lines are the rolling average number of contacts across the 50 frames. Red lines mark frames as shown in Fig. 2. Dashed blue line marks frame from which contact surface area was extracted to represent the loaded state.

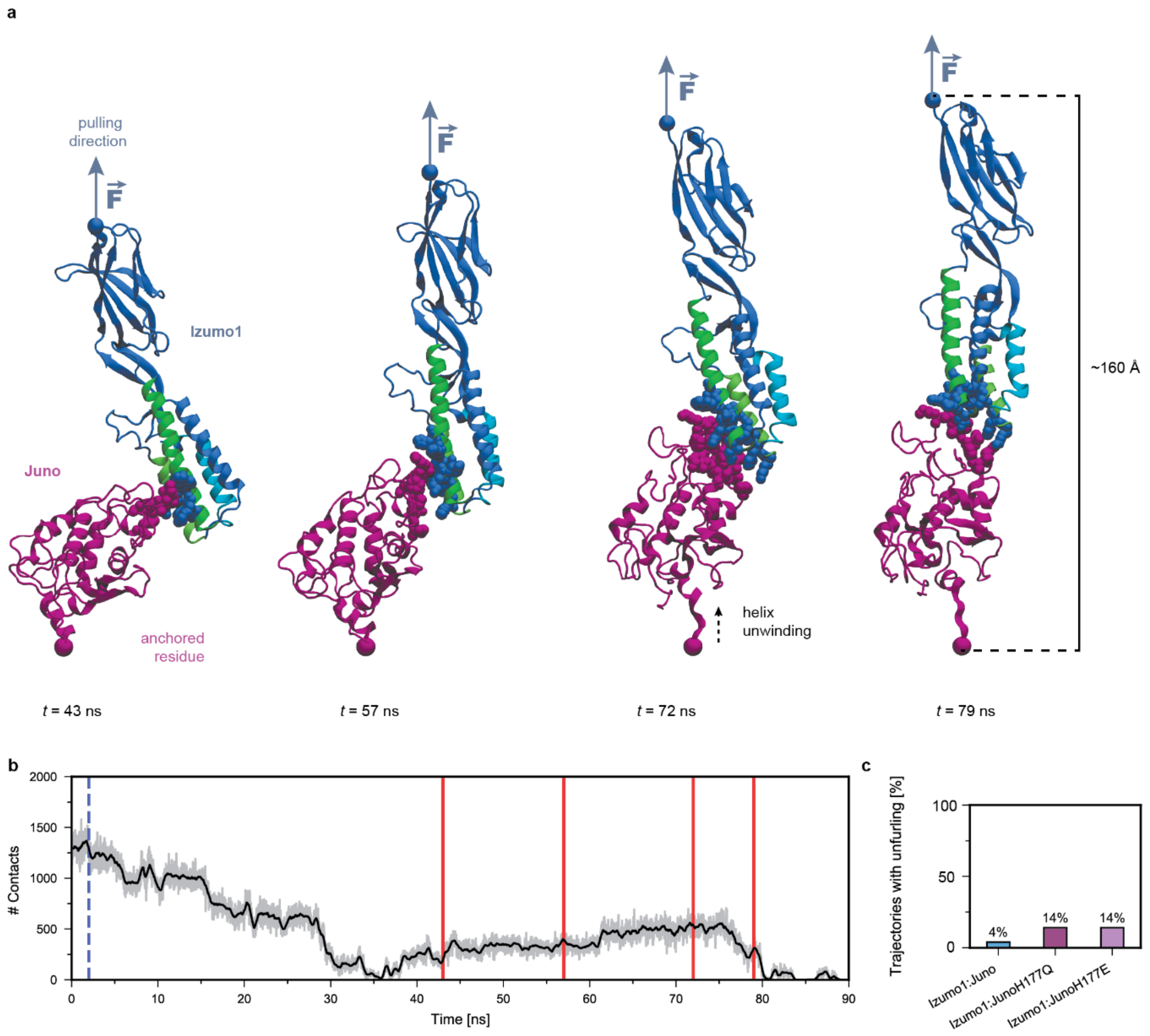

Supplementary Fig. 10: The unwinding of the C-terminal helix of Juno as observed in some SMD simulations of Izumo1:Juno complex under constant velocity pulling. a, Representative snapshots of structural deformations in the secondary structure of Juno during unbinding. As the known binding interface is lost, new interactions are gradually formed, prolonging the interaction between Izumo1 and Juno until the C-terminal of Juno unwinds. **b**, Number of contacts between Izumo1 and Juno traced as a function of time. Gray traces represent number of contacts for individual frames and the black line represents the rolling average number of contacts across the 50 frames. Red lines mark frames shown in **a**. **c**, Percentage of trajectories that show unwinding of the Juno C-terminal α-helix in wild-type Izumo1:Juno (n = 2), Izumo1:JunoH177Q (n = 7), and Izumo1:JunoH177E (n = 7).

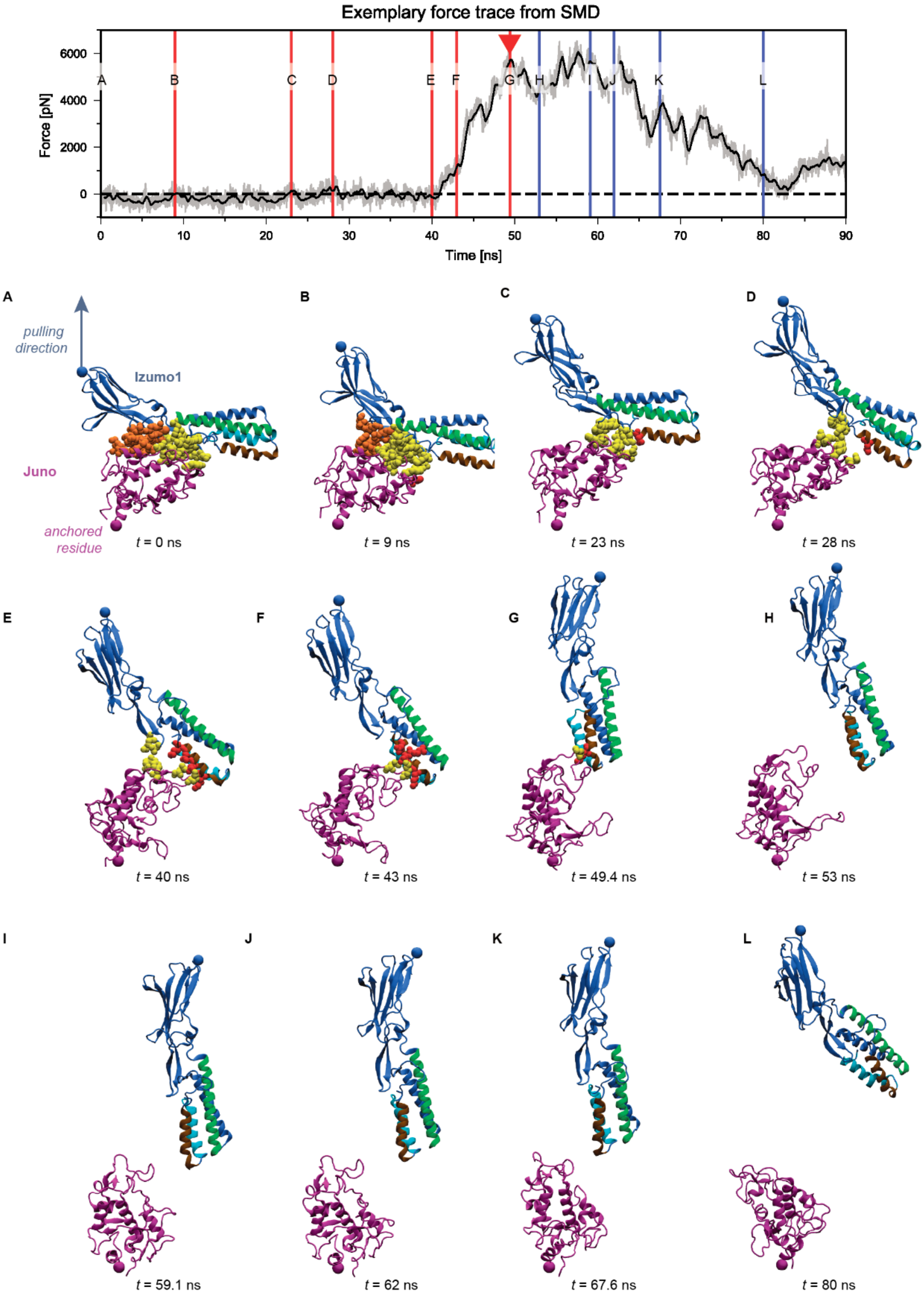

Exemplary force trace from SMD
Force [pN]
Time [ns]
0
2000
4000
6000
0
10
20
30
40
50
60
70
80
90
A
B
C
D
E
F
G
H
I
J
K
L
pulling direction
Izumo1
Juno
anchored residue
t = 0 ns
t = 9 ns
t = 23 ns
t = 28 ns
t = 40 ns
t = 43 ns
t = 49.4 ns
t = 53 ns
t = 59.1 ns
t = 62 ns
t = 67.6 ns
t = 80 ns

Supplementary Fig. 11: Force *vs.* time trace with structural snapshots representative of the three-state wild-type Izumo1:Juno unbinding subtype observed in SMD simulations. Different residues are involved in the Cluster 3 (red) formation in three-state subtype trajectories when compared to the majority of the three-state unbinding trajectories. Letters and lines mark timepoints at which snapshots were taken and the red triangle marks the first rupture event. Gray traces represent force for individual frames and black lines are the rolling average force across the 50 frames. The number of contacts between Izumo1 and Juno as a function of time can be found on Supplementary Fig. 9c. Preview of the unbinding process can be observed in Supplementary Movie 3.

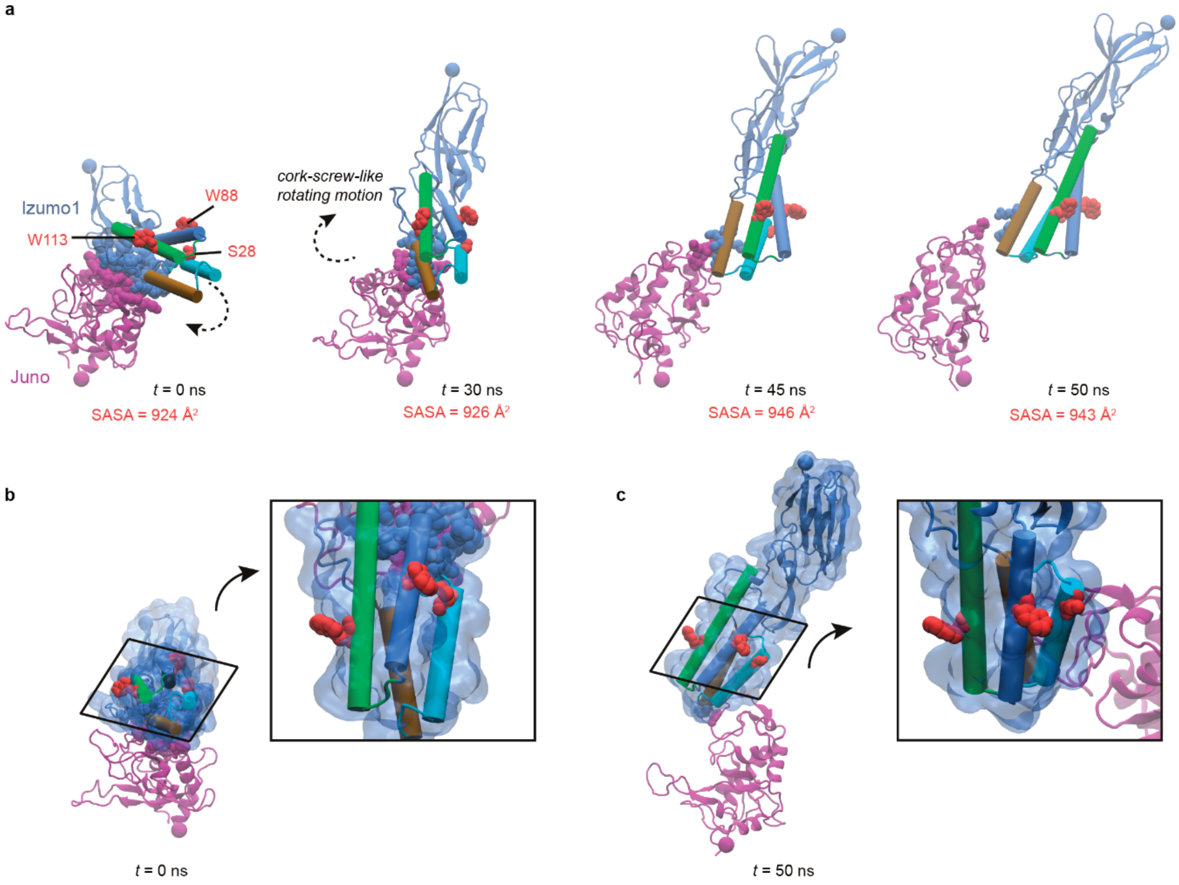

Supplementary Fig. 12: Reorganization of membrane fusion-essential residues in the three-state wild-type Izumo1:Juno unbinding subtype observed during SMD simulations. **a**, Upwards pulling of Izumo1 results in a cork-screw-like rotation of Izumo1 with respect to Juno. During this process, the membrane fusion-essential residues; W113, W88 and S28[4]; partially buried in the 4-helix bundle of Izumo1 (shown in red), become increasingly solvent exposed (SASA calculated using water probe radius of 1.4 Å reported for each frame) and aligned across same interaction plane. Such conformational changes would allow for larger hydrophobic interaction paths between Izumo1 and the egg cell membrane, supporting the membrane fusion activity of Izumo1 expressing cells. **b**, Initial orientation of W113, W88 and S28 in the 4-helix bundle of Izumo1. **c**, Final orientation of W113, W88 and S28, now aligned to form a large hydrophobic patch on the Izumo1 surface. See also Supplementary Movie S4.

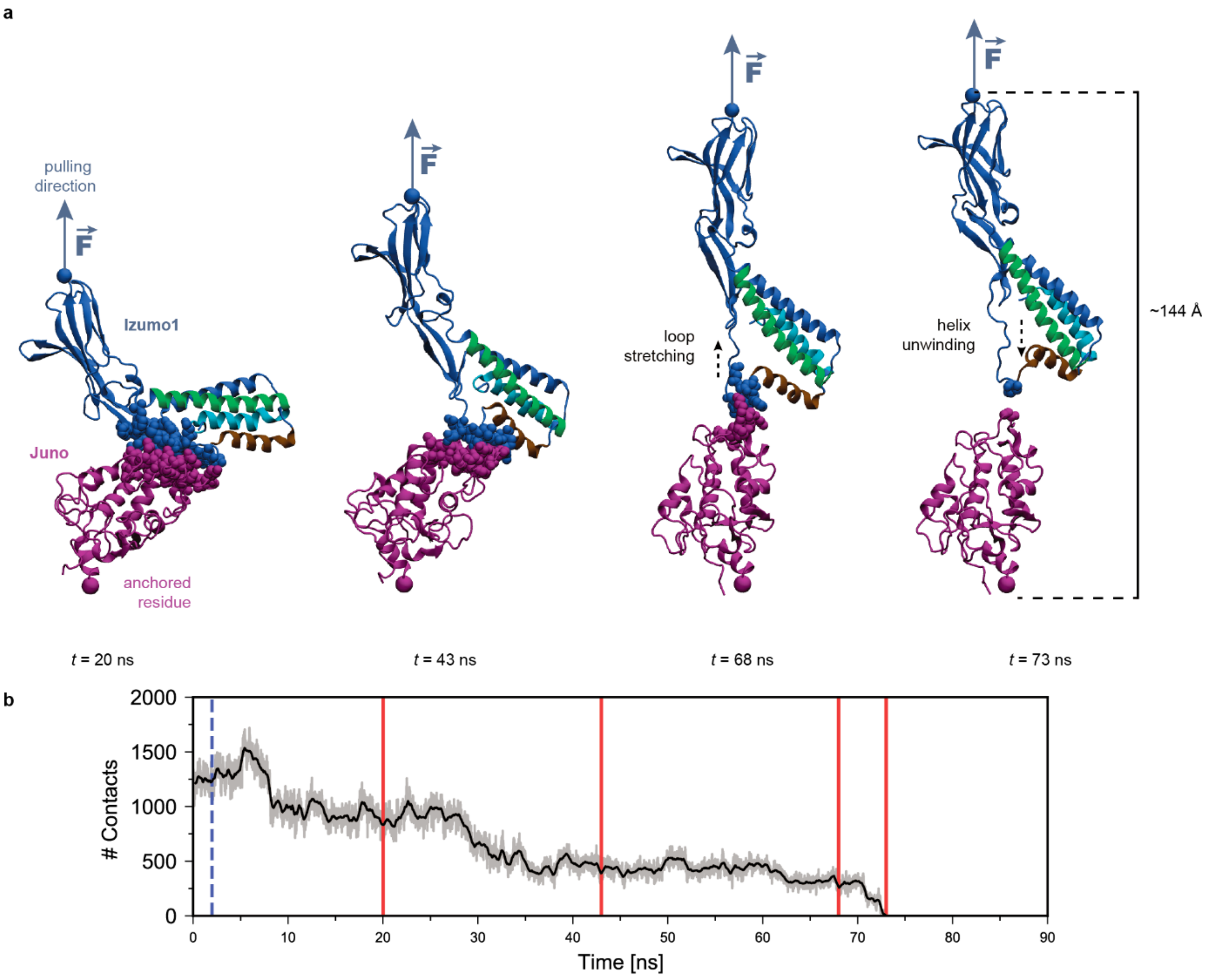

Supplementary Fig. 13: The partial unwinding of Izumo1 4-helix bundle stochastically observed in SMD simulations of the Izumo1:JunoH177Q complex. a, Representative snapshots of structural deformations of the Izumo1 secondary structure during unbinding. The Cluster 2 interactions formed a strong association between Juno and the deformed region of Izumo1 around the residue E71. Where SMD simulations are performed at 0.001 Å·ps^{-1}, it is possible that the 4-helix bundle of Izumo1 undergoes further rearrangements at physiological speeds. **b**, Number of contacts between Izumo1 and Juno traced as a function of time. Gray traces represent number of contacts for individual frames and the black line represents the rolling average number of contacts across the selected frames. Red lines mark frames shown in **b**.

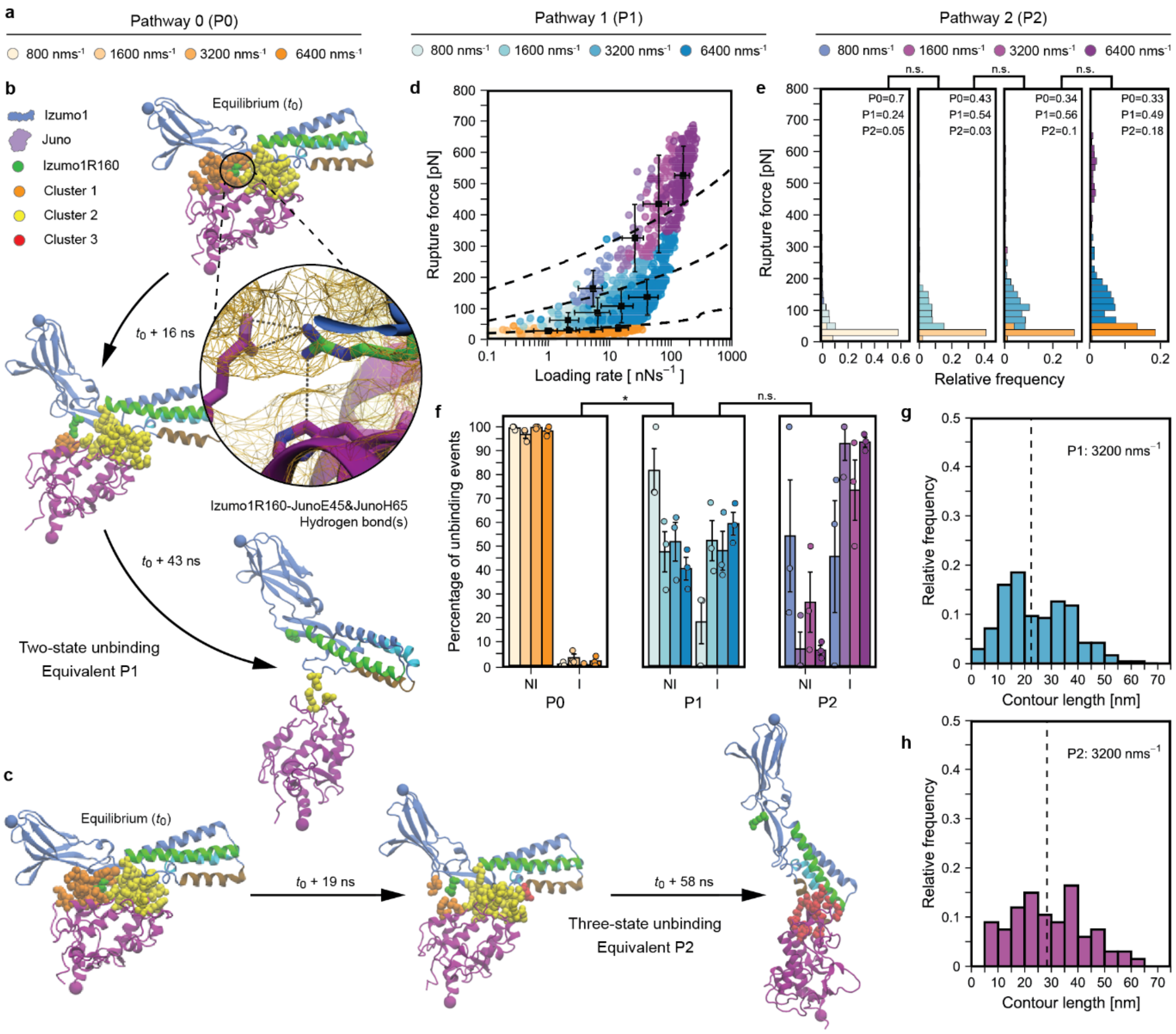

a
Pathway 0 (P0)
800 nms-1 1600 nms-1 3200 nms-1 6400 nms-1
Pathway 1 (P1)
800 nms-1 1600 nms-1 3200 nms-1 6400 nms-1
Pathway 2 (P2)
800 nms-1 1600 nms-1 3200 nms-1 6400 nms-1
b
Izumo1
Juno
Izumo1R160
Cluster 1
Cluster 2
Cluster 3
Equilibrium (t0)
t0 + 16 ns
Izumo1R160-JunoE45&JunoH65
Hydrogen bond(s)
t0 + 43 ns
Two-state unbinding
Equivalent P1
c
Equilibrium (t0)
t0 + 19 ns
t0 + 58 ns
Three-state unbinding
Equivalent P2
d
Rupture force [pN]
Loading rate [nNs−1]
e
P0=0.7 P1=0.24 P2=0.05
P0=0.43 P1=0.54 P2=0.03
P0=0.34 P1=0.56 P2=0.1
P0=0.33 P1=0.49 P2=0.18
n.s.
Rupture force [pN]
Relative frequency
f
Percentage of unbinding events
NI I
P0 P1 P2
*
n.s.
g
P1: 3200 nms−1
Relative frequency
Contour length [nm]
h
P2: 3200 nms−1
Relative frequency
Contour length [nm]

Supplementary Fig. 14: Supplementary constant speed AFM-SMFS data for Izumo1R160A:Juno. a, Legend for P0, P1, and P2 unbinding events at each constant pulling speed. **b**, Equilibrated starting structure ($t = t_0$) for Izumo1:Juno showing Izumo1R160 (green) and amino acids clusters 1 (orange), 2 (yellow) and 3 (red). Izumo1:Juno complex unbinding though the two-state pathway. **c**, Equilibrated starting structure ($t = t_0$), color coded as per panel **b**, with Izumo1:Juno unbinding through the third-state pathway. **d**, Izumo1R160A:Juno rupture force *vs.* loading rates (median, error bars ± MAD). Dashed lines show the loading rate *vs*. rupture force behavior predicted by DHS model fitting (Eq. (6), Eq. (7), Supplementary Table 1). **e**, Izumo1R160A:Juno rupture force histograms (bins = 20 pN). **f**, Direct Izumo1R160A:Juno unbinding (NI) compared to Izumo1R160A:Juno unbinding through intermediate folded states (I) (mean, error bars ± SEM). **g**, Contour length distributions for Izumo1R160A:Juno rearrangement prior to rupture through P1 at 3200 nms^{-1} (n = 238). **h**, Contour length distributions for Izumo1R160A:Juno rearrangement prior to rupture through P2 at 3200 nms^{-1} (n = 67). Data in **d** and **e** represent three pooled independent experiments that were divided based on unbinding pathway for DHS model fitting (P0: n = 359, 330, 298. P1: n = 584, 491, 379. P2: n = 314, 221, 239). For **f,** independent replicates were kept separate and further divided based on pathway and constant pulling speed (P0 800; 1600; 3200; 6400 nms^{-1}: n = 107, 276, 102; 102, 288, 139; 46, 129, 118; 51, 145, 120. P1 800; 1600; 3200; 6400 nms^{-1}: n = 66, 80, 22; 106, 104, 57; 199, 176, 108; 177, 181, 107. P2 800, 1600; 3200; 6400 nms^{-1}: n = 27, 9, 1; 19, 8, 1; 48, 30, 4; 81, 65, 20). For **e**, significance (*p < 0.05) was determined using Kurskal-Wallis tests followed by pairwise Wilcoxon rank-sum tests, exact n-values provided in the Source Data file. For **f**, significance (*p < 0.05) was determined using Kurskal-Wallis tests followed by Dunn tests (Šidák-adjusted), P-values: 0.013 (P0 vs P1), < 0.0001 (P0 vs P2), 0.061 (P1 vs P2). Full statistical details are provided in the Methods.

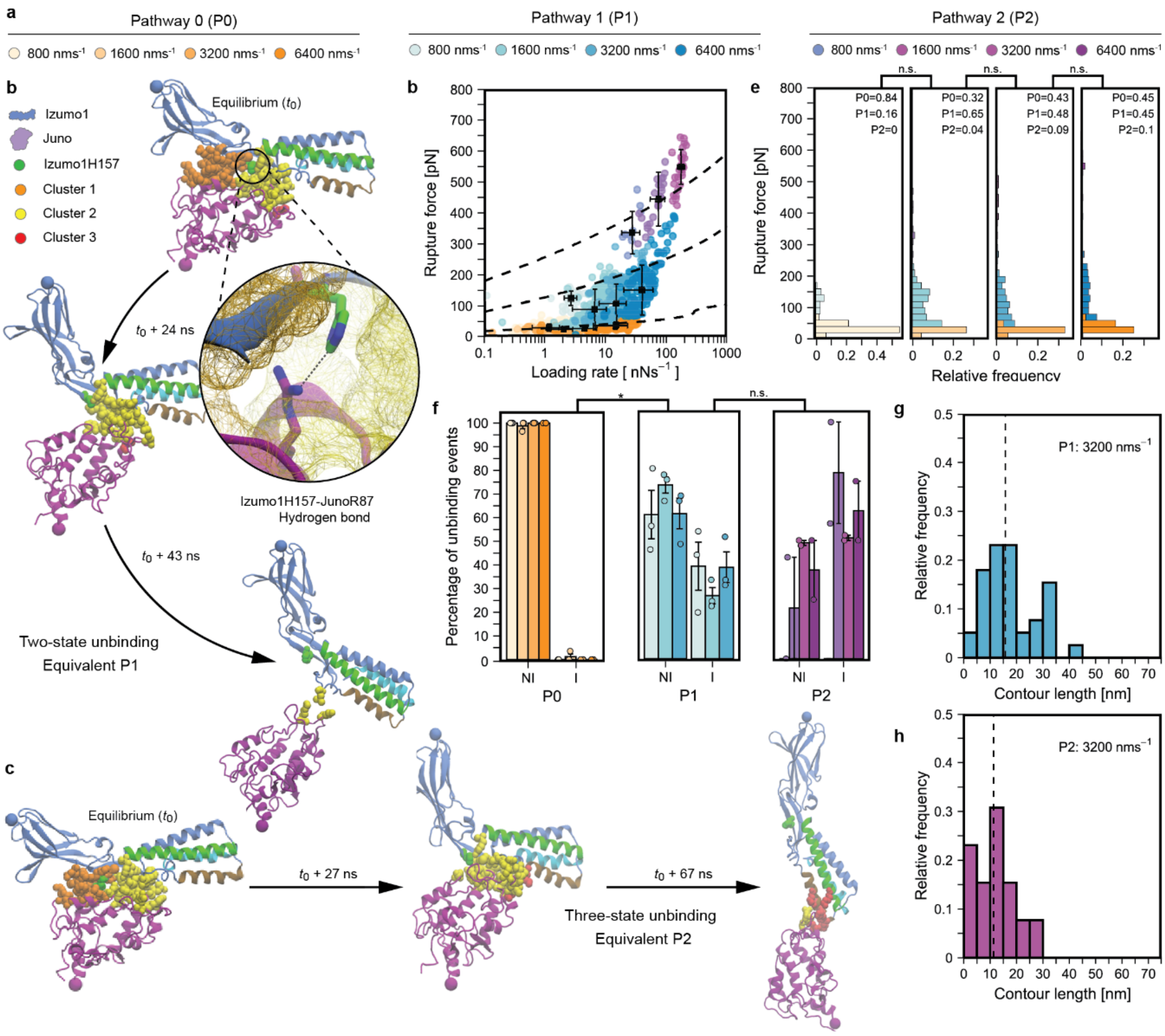
a
Pathway 0 (P0)
800 nms-1 1600 nms-1 3200 nms-1 6400 nms-1
Pathway 1 (P1)
800 nms-1 1600 nms-1 3200 nms-1 6400 nms-1
Pathway 2 (P2)
800 nms-1 1600 nms-1 3200 nms-1 6400 nms-1
b
Izumo1
Juno
Izumo1H157
Cluster 1
Cluster 2
Cluster 3
Equilibrium (t0)
t0 + 24 ns
Izumo1H157-JunoR87
Hydrogen bond
t0 + 43 ns
Two-state unbinding
Equivalent P1
c
Equilibrium (t0)
t0 + 27 ns
t0 + 67 ns
Three-state unbinding
Equivalent P2
Rupture force [pN]
Loading rate [nNs−1]
e
P0=0.84 P1=0.16 P2=0
P0=0.32 P1=0.65 P2=0.04
P0=0.43 P1=0.48 P2=0.09
P0=0.45 P1=0.45 P2=0.1
n.s.
Relative frequency
f
Percentage of unbinding events
NI
I
P0
P1
P2
g
P1: 3200 nms−1
Relative frequency
Contour length [nm]
h
P2: 3200 nms−1
Relative frequency
Contour length [nm]

Supplementary Fig. 15: Supplementary constant speed AFM-SMFS data for Izumo1H157A:Juno. a, Legend for P0, P1, and P2 unbinding events at each constant pulling speed. **b**, Equilibrated starting structure ($t = t_0$) for Izumo1:Juno showing Izumo1H157 (green) and amino acids clusters 1 (orange), 2 (yellow) and 3 (red). Izumo1:Juno complex unbinding though the two-state pathway. **c**, Equilibrated starting structure ($t = t_0$), color coded as per panel **b**, with Izumo1:Juno unbinding through the three-state pathway. **d**, Izumo1H157A:Juno rupture force *vs.* loading rates (median, error bars ± MAD). Dashed lines show the loading rate *vs.* rupture force behavior predicted by DHS model fitting (Eq. (6), Eq. (7), Supplementary Table 1). **e**, Izumo1H157A:Juno rupture force histograms (bins = 20 pN). **g**, Direct Izumo1H157A:Juno unbinding (NI) compared to Izumo1H157A:Juno unbinding through intermediate folded states (I) (mean, error bars ± SEM). **g**, Contour length distributions for Izumo1H157A:Juno rearrangements occurring prior to rupture through P1 at 3200 nms^{-1} (n = 39). **h**, Contour length distributions for Izumo1H157A:Juno rearrangements occurring prior to rupture through P2 at 3200 nms^{-1} (n = 13). Data in **d** and **e** represent three pooled independent experiments that were divided based on unbinding pathway for DHS model fitting (P0: n = 174, 118, 131. P1: n = 114, 53, 94. P2: n = 118, 89, 102). For **f,** independent replicates were kept separate and further divided based on pathway and constant pulling speed (P0 800; 1600; 3200; 6400 nms^{-1}: n = 18, 58, 53; 47, 56, 62; 55, 33, 38; 56, 27, 49. P1 1600; 3200; 6400 nms^{-1}: n = 13, 41, 46; 37, 36, 66; 33, 45, 54. P2 1600; 3200; 6400 nms^{-1}: n = 3, 7, 0; 2, 23, 0; 2, 28, 0). For **e**, significance (*p < 0.05) was determined using Kurskal-Wallis tests followed by pairwise Wilcoxon rank-sum tests, exact n-values are provided in the Source Data file. For **f**, significance (*p < 0.05) was determined using Kurskal-Wallis tests followed by Dunn tests (Šidák-adjusted), P-values: 0.002 (P0 vs P1), < 0.001 (P0 vs P2), 0.183 (P1 vs P2).Full statistical details are provided in the Methods.

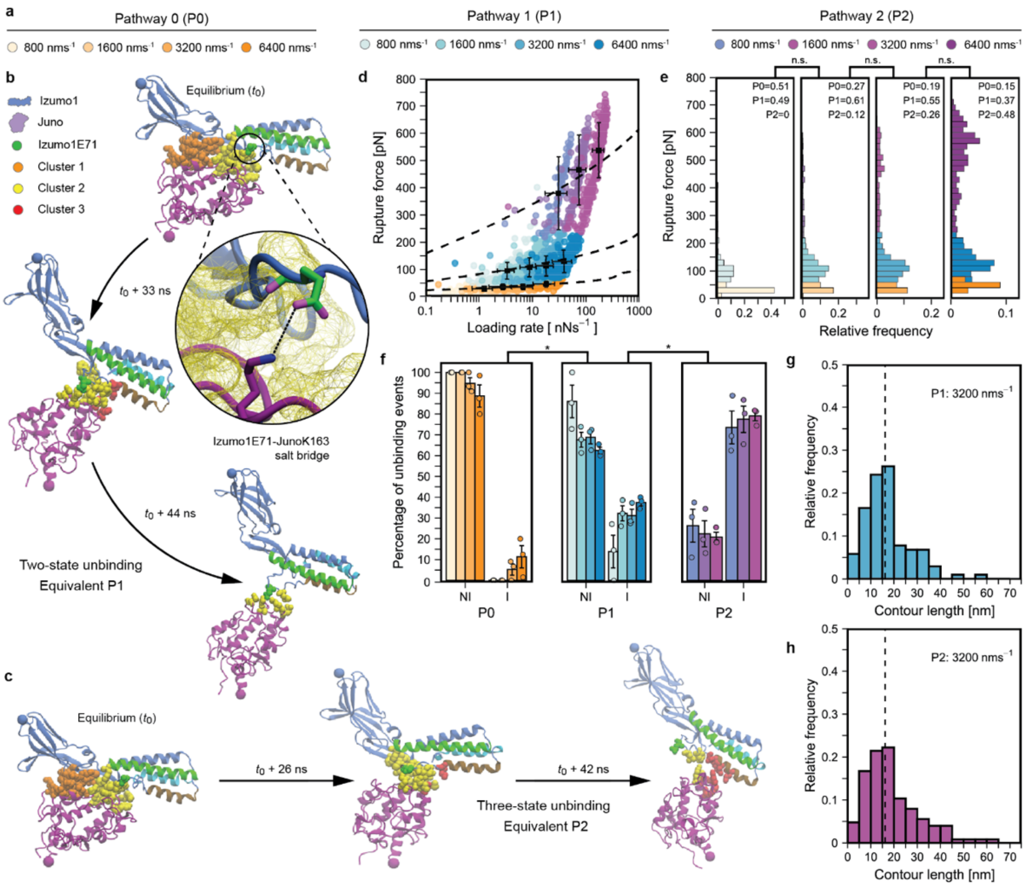

Supplementary Fig. 16: Supplementary constant speed AFM-SMFS data for Izumo1E71A:Juno. a, Legend for P0, P1, and P2 unbinding events at each constant pulling speed. **b**, Equilibrated starting structure ($t = t_0$) for Izumo1:Juno showing Izumo1E71 (green) and amino acids clusters 1 (orange), 2 (yellow) and 3 (red). Izumo1:Juno complex unbinding though the two-state pathway. **c**, Equilibrated starting structure ($t = t_0$), color coded as per panel **b**, with Izumo1:Juno unbinding through the three-state pathway. **d**, Izumo1E71A:Juno rupture force *vs.* loading rates (median, error bars ± MAD). Dashed lines show the loading rate vs. rupture force behavior predicted by DHS model fitting (Eq. (6), Eq. (7), Supplementary Table 1). **e**, Izumo1E71A:Juno rupture force histograms (bins = 20 pN). **f**, Direct Izumo1E71A:Juno unbinding (NI) compared to Izumo1E71A:Juno unbinding through intermediate folded states (I) (mean, error bars ± SEM). **g**, Contour length distributions for Izumo1E71A:Juno rearrangements occurring prior to rupture through P1 at 3200 nms^{-1} (n = 103). **h**, Contour length distributions for Izumo1E71A:Juno rearrangements occurring prior to rupture through P2 at 3200 nms^{-1} (n = 126). Data in **d** and **e** represent three pooled independent experiments that were divided based on unbinding pathway for DHS model fitting (P0: n = 35, 20, 10. P1: n = 311, 206, 176. P2: n = 358, 506, 565). For **f,** independent replicates were kept separate and further divided based on pathway and constant pulling speed (P0 800; 1600; 3200; 6400 nms^{-1}: n = 33, 165, 17; 22, 124, 21; 11, 91, 15; 5, 66, 16. P1 800; 1600; 3200; 6400 nms^{-1}: n = 151, 13, 41; 180, 44, 149; 127 75, 140; 60, 42, 113. P2 1600; 3200; 6400 nms^{-1}: n = 27, 8, 36; 53, 29, 76; 64, 43, 175). For **e**, significance (*p < 0.05) was determined using Kurskal-Wallis tests followed by pairwise Wilcoxon rank-sum tests, exact n-values are provided in the Source Data file. For **f**, significance (*p < 0.05) was determined using Kurskal-Wallis tests followed by Dunn tests (Šidák-adjusted), P-values: 0.013 (P0 vs P1), < 0.001 (P0 vs P2), 0.326 (P1 vs P2).Full statistical details are provided in the Methods.

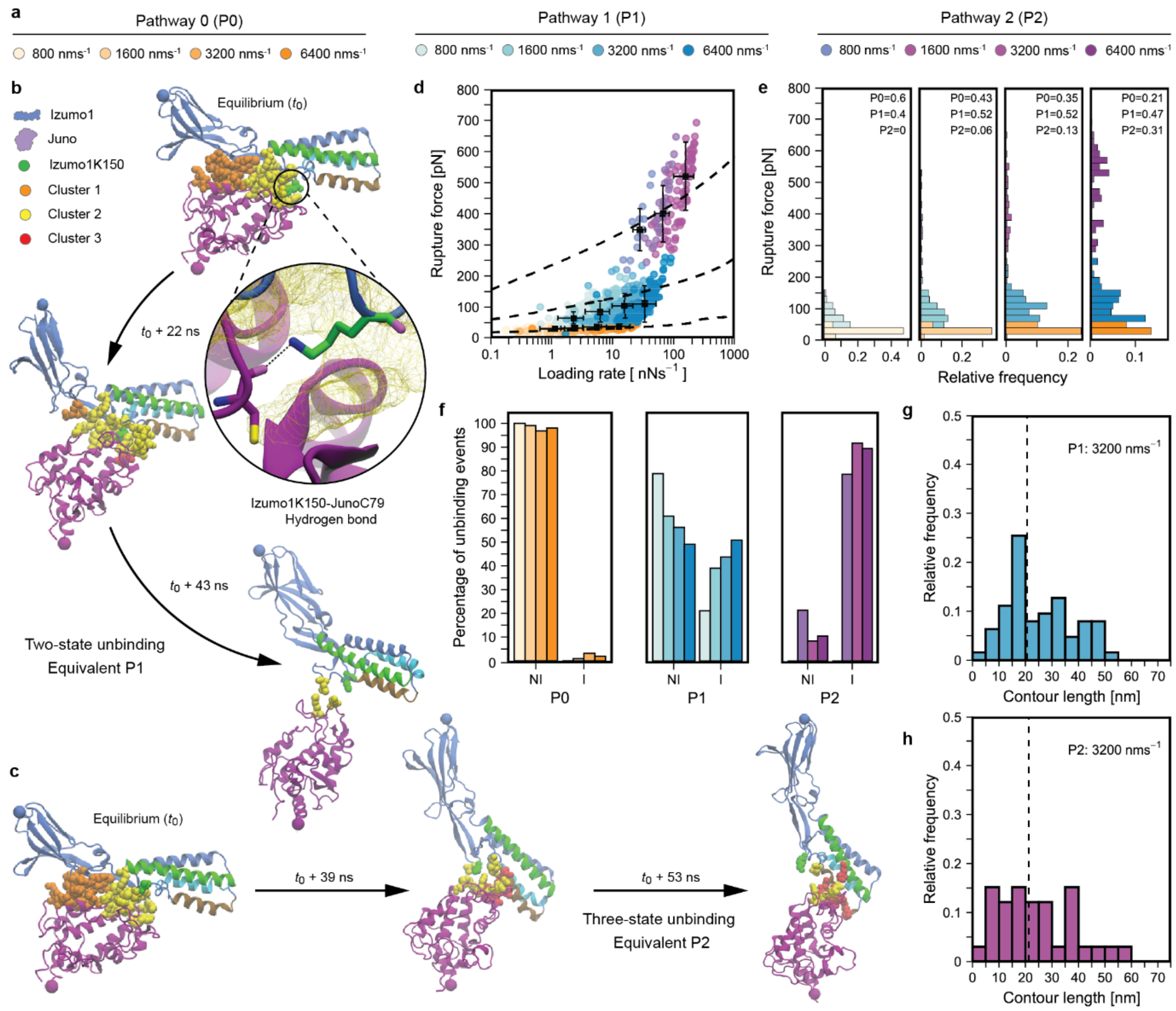

Supplementary Fig. 17: Supplementary constant speed AFM-SMFS data for Izumo1K150:Juno. a, Legend for P0, P1, and P2 unbinding events at each constant pulling speed. **b**, Equilibrated starting structure ($t = t_0$) for Izumo1:Juno showing Izumo1K150 (green) and amino acids clusters 1 (orange), 2 (yellow) and 3 (red). Izumo1:Juno complex unbinding though the two-state pathway. **c**, Equilibrated starting structure ($t = t_0$), color coded as per panel **b**, with Izumo1:Juno unbinding through the three-state pathway. **d**, Izumo1K150A:Juno rupture force *vs.* loading rates (median, error bars ± MAD). Dashed lines show the loading rate *vs*. rupture force behavior predicted by DHS model fitting (Eq. (6), Eq. (7), Supplementary Table 1). **e**, Izumo1K150A:Juno rupture force histograms (bins = 20 pN). **f**, Direct Izumo1K150A:Juno unbinding (NI) compared to Izumo1K150A:Juno unbinding through intermediate folded states (I) (mean, error bars ± SEM). **g**, Contour length distributions for Izumo1K150A:Juno rearrangements occurring prior to rupture through P1 at 3200 nms^{-1} (n = 63). **h**, Contour length distributions for Izumo1K150A:Juno rearrangements occurring prior to rupture through P2 at 3200 nms^{-1} (n =33). Data in **d** and **e** represent a single experiment divided based on unbinding pathway for DHS model fitting (P0: n = 331. P1: n = 338. P2: n = 294). For **f,** data was further divided based on pathway and constant pulling speed (P0 800; 1600; 3200; 6400 nms^{-1}: n = 135, 106, 95, 52. P1 800; 1600; 3200; 6400 nms^{-1}: n = 90, 128, 144, 114. P2 1600; 3200; 6400 nms^{-1}: n = 14, 36, 76). Hypothesis testing was not performed for this variant due to long-term instability of independent experimental replicates, see supplementary text for specific details.

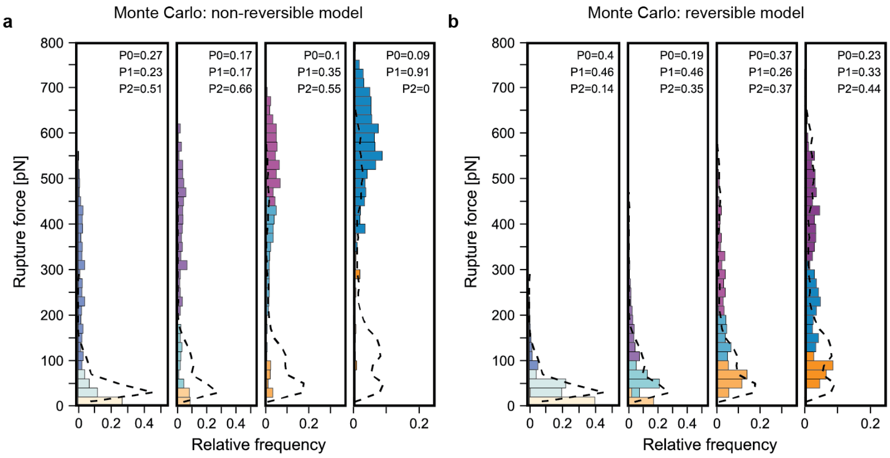

Supplementary Fig. 18: Constant speed Monte Carlo simulations of multi-pathway Izumo1:Juno unbinding through intermediate states. **a**, Non-reversible transitions between intermediate states that give rise to P0, P1, and P2 unbinding pathways. Residual sum of squares fit to the binned experimental and Monte Carlo simulated rupture force distributions (bins = 20 pN) is 0.189. **b**, Reversible transitions between intermediate states that give rise to P0, P1, and P2 unbinding pathways. Residual sum of squares fit to the binned experimental and Monte Carlo simulated rupture force distributions (bins = 20 pN) is 0.173. For all constant speed Monte Carlo simulations, time step = 1000, *n* = 500, and dashed lines represent a smoothed approximation of the experimental rupture force histogram distribution (fitted using a cubic spline).

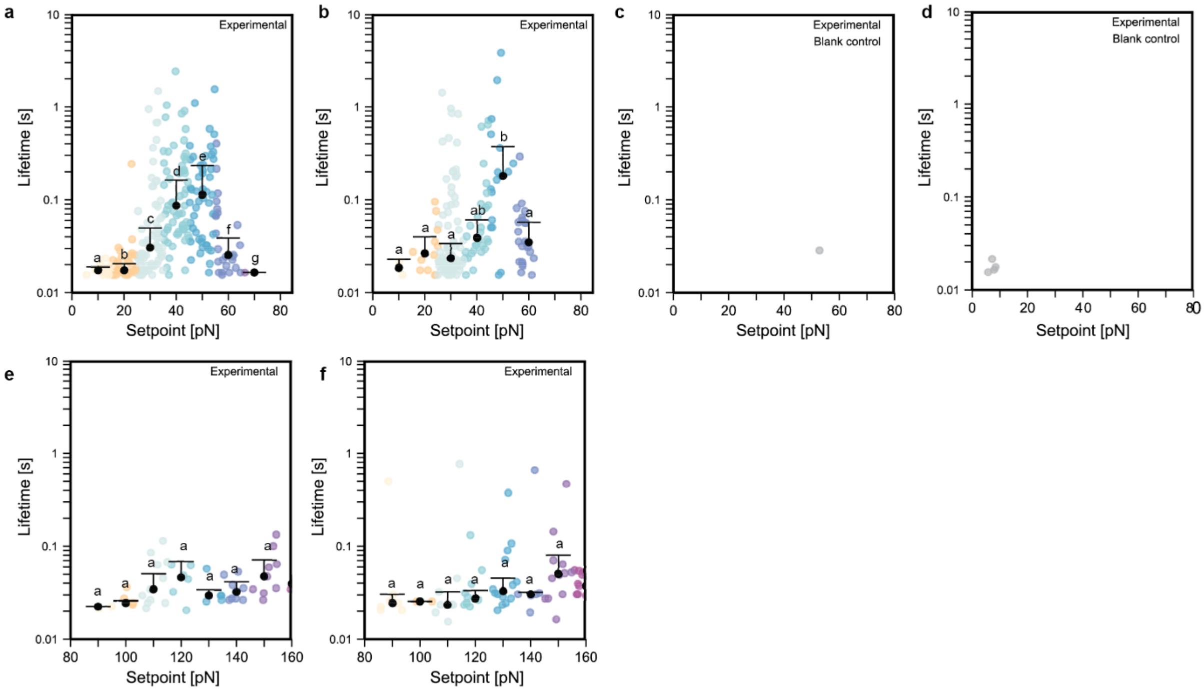

Supplementary Fig. 19: Force clamp AFM-SMFS Izumo1:Juno biological replicates and blank controls. a–b, Biological replicate 2 and 3 for Izumo1:Juno force clamp AFM-SMFS performed in the 10-80 pN setpoint range (10 pN bins , *n* = 15, 34, 68, 60, 49, 25, 1. And, *n* = 2, 12, 73, 30, 17, 24; respectively). **c–d**, Representative blank controls for Izumo1:Juno force clamp AFM-SMFS performed in the 10-80 pN setpoint range. Representative blank controls for Izumo1:Juno force clamp AFM-SMFS performed in the 80-160 pN setpoint range not included due to the absence of non-specific binding events (*n* = 1 and *n* = 3, respectively). **e–f**, Biological replicate 2 and 3 as representative examples for Izumo1:Juno force clamp AFM-SMFS performed in the 80-160 pN setpoint range (*n* = 3, 6, 11, 6, 5, 9, 9, 12. And, *n* = 7, 3, 8, 11, 14, 6, 13, 12, respectively). Colored dots represent individual bond lifetime measurements. Dark gray dots represent non-specific force clamping events. Black dots represent median bond lifetime (error bars ± MAD) across 10 pN bins. Shared letters indicate the absence of statistically significant differences ($p > 0.05$) using Kruskal–Wallis tests followed by Dunn's multiple-comparison tests (Šidák-adjusted), Exact *P*-values are provided in the Source Data file (see supplementary text for specific details). Blank control consists of the Izumo1 functionalized tips, with activity established using constant speed SMFS, probed against cover glasses with only the AFM-handle functionalized (*i.e.* no Juno).

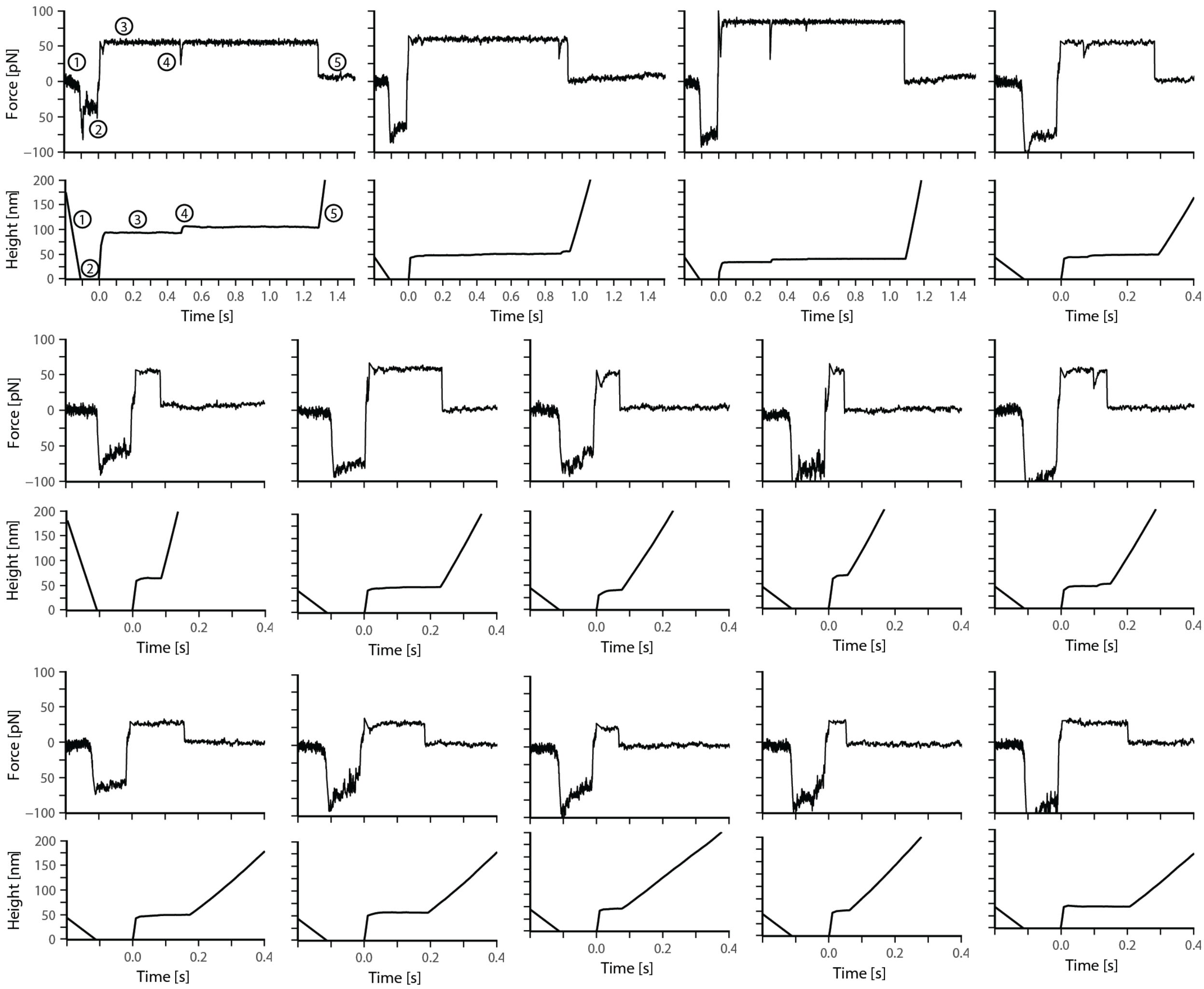

Supplementary Fig. 20: Force clamp AFM-SMFS representative curves. Top panels show force *vs.* time curves. Bottom panels show height *vs.* time curves. (1) Shows the approach of the cantilever to the surface. (2) Shows the pause of the cantilever tip at the surface for 0.2 s. (3) Shows the retraction of the cantilever to the target setpoint. (4) Where present in force *vs.* time and height *vs.* time curves this feature shows the presence of a contour length change in the Izumo1:Juno complex. (5) Shows complex rupture and resumes retraction of the cantilever.

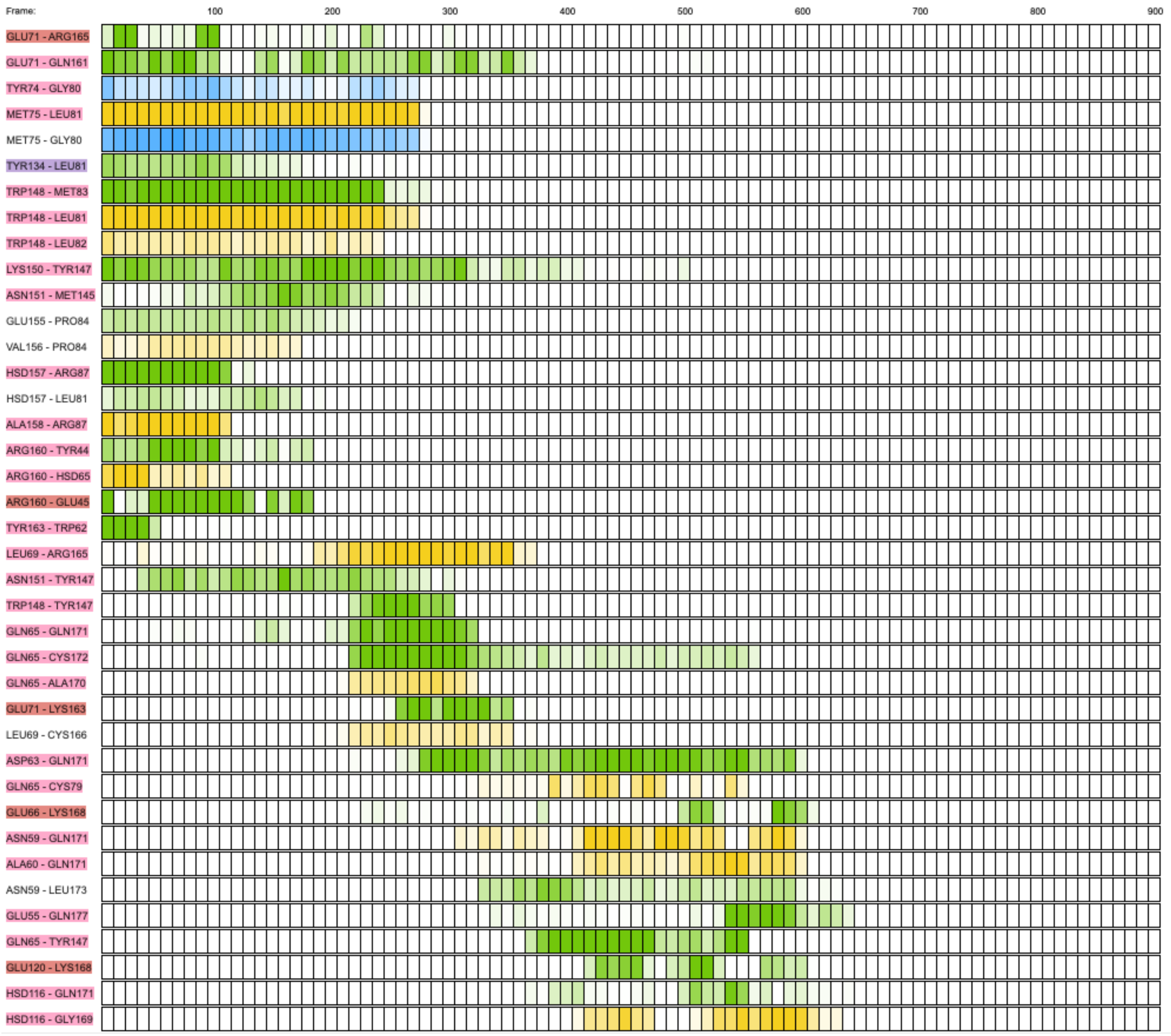

Supplementary Fig. 21: Exemplary time series of the non-covalent interactions as observed in the three-state unbinding pathway of the Izumo1:JunoH177Q mutant complex in SMD simulations. In simulations of Izumo1:JunoH177Q, fewer trajectories showed entry into the third unbinding pathway. Overall, the residues on Izumo1 that formed prolonged interactions with JunoH177Q were similar to those interacting with Juno in the wild-type Izumo1:Juno complex. However, where Juno H177 was unavailable to form strong interactions, Juno Q177 formed only one hydrogen bond with Izumo1 E55. Supplementary Movie 5 shows the preview of the corresponding simulation. The first residue in the label belongs to Izumo1, and the second residue belongs to Juno. The color of the label is determined by interaction type and geometry: red, salt bridge; blue, hydrophobic; and pink, hydrogen bond. Each box corresponds to the equal trajectory segment, their color signifies interaction type and the intensity shows the strength of the interaction: green, side chain/side chain; yellow, side chain/backbone; blue, backbone/backbone. PyContact[7] was used to extract this data with a filter of mean score value > 0.25 for trajectory of the total simulation time of 90 ns (900 frames extracted).

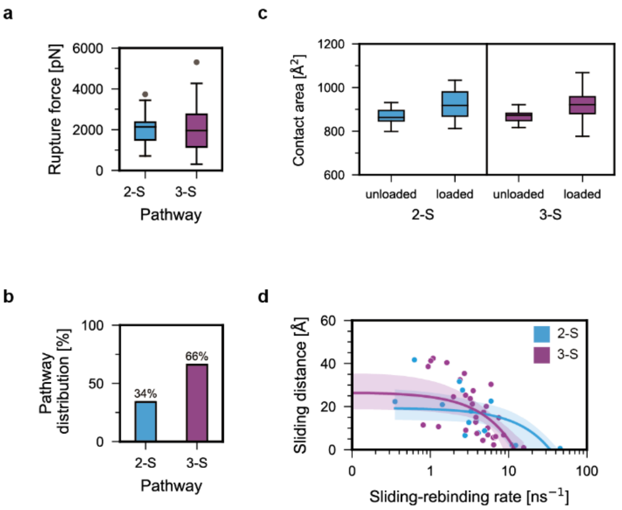

Supplementary Fig. 22: SMD simulations of Izumo1:JunoH177E unbinding. **a**, Peak forces extracted from two-state (2-S) and three-state (3-S) SMD simulations of Izumo1:JunoH177E complexes (median, error bars ± MAD). **b**, Two-state and three-state unbinding pathway distribution for Izumo1:H177E across 50 SMD trajectories. **c**, Observed increases in contact area between Izumo1 and JunoH177E in unloaded (t = 0 ns) and loaded (t = 0 ns) complexes for the two-state and three-state pathways. **d**, Sliding-rebinding distance *vs.* sliding-rebinding rate for the force-dependent rearrangement of Izumo1:JunoH177E complexes in the two-state and three-state pathways (shaded areas, 95% CI). Boxplots show the median, interquartile range (IQR), and whiskers extending to 1.5 times the IQR. Points beyond the whiskers are outliers. For two-state and three-state, n = 17 and n = 33, respectively.

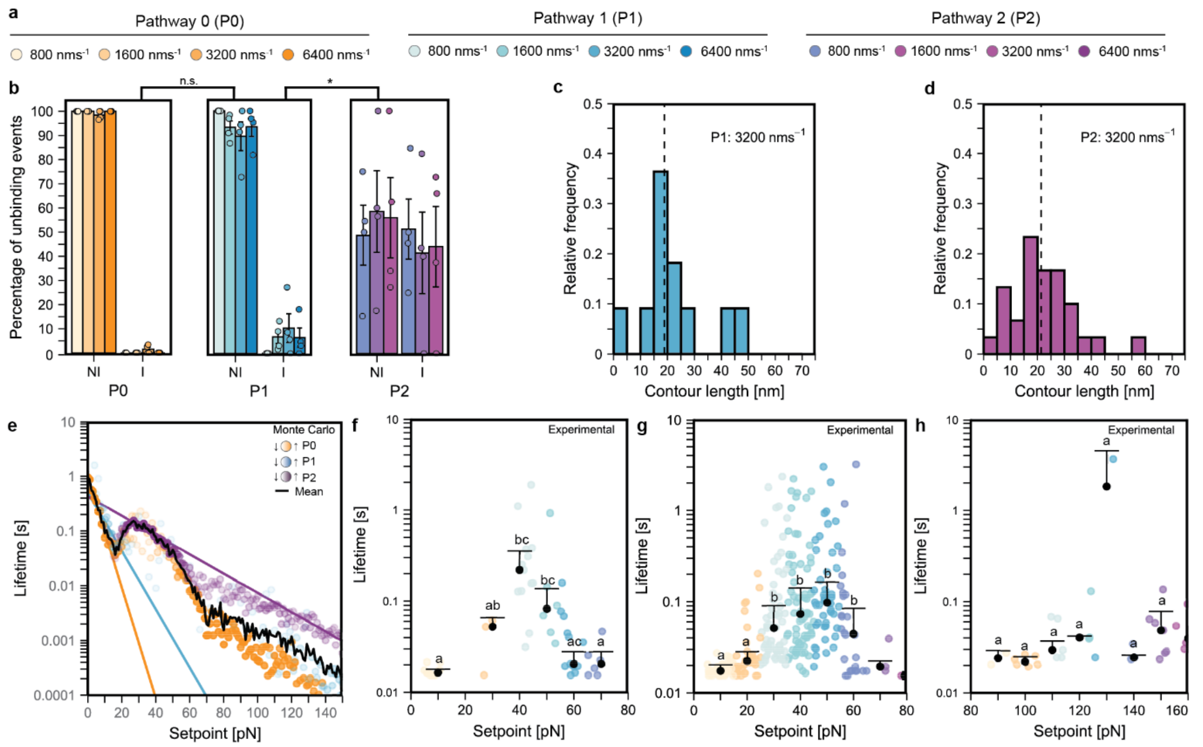

Supplementary Fig. 23: Monte Carlo force clamp and supplemental AFM-SMFS data for Izumo1:JunoH177Q. **a**, Legend for P0, P1, and P2 unbinding events at each constant pulling speed. **b**, Direct Izumo1:JunoH177Q unbinding (NI) compared to unbinding through intermediate folded states (I) (mean, error bars ± SEM). Significance (**p* < 0.05) determined using Kurskal-Wallis tests followed by Dunn tests (Šidák-adjusted), *P*-values: 0.053 (P0 vs P1), < 0.001 (P0 vs P2), 0.005 (P1 vs P2). For **b,** data was divided based on pathway and constant pulling speed (P0 800; 1600; 3200; 6400 nms^{-1}: *n* = 83, 108, 72, 28; 62, 79, 37, 40; 77, 68, 29, 35; 69, 21, 60, 14. P1 800; 1600; 3200; 6400 nms^{-1}: *n* = 15, 5, 5, 18; 60, 33, 30, 32; 23, 52, 22, 32; 42, 45, 63, 22. P2 1600; 3200; 6400 nms^{-1}: *n* = 4, 3, 0, 0; 8, 22, 13, 2; 23, 15, 17, 2; 47, 1, 16, 11). **c**, Contour length distributions for rearrangements during P1 at 3200 nms^{-1} (*n* = 11). **d**, Contour length distributions for rearrangements during P2 at 3200 nms^{-1} (*n* = 30). **e**, Monte Carlo simulated force clamp AFM-SMFS for Izumo1:JunoH177Q. Colored dots represent mean bond lifetime, intensity represents the relative frequency. Colored lines represent lifetimes calculated directly from BE-derived kinetic parameters (Supplementary Table 1**,** Eq. (11)). Black line represents the mean bond lifetime across all three pathways. **f–g**, Biological replicate 2 and 3 as representative examples for Izumo1:Juno force clamp AFM-SMFS performed in the 80-160 pN setpoint range (*n* = 11, 3, 13, 7, 13, 7. And, *n* = 39, 39, 61, 70, 46, 26, 3, 1, respectively). **h**, Representative force clamp AFM-SMFS experiment performed between 90-160 pN (*n* = 2, 6, 5, 3, 2, 3, 7, 7). Colored dots represent individual bond lifetime measurements. Black dots represent median bond lifetime (error bars ± MAD) across 10 pN bins. Shared letters indicate the absence of significance (*p* > 0.05) determined using Kruskal–Wallis tests followed by Dunn's multiple-comparison tests (Šidák-adjusted), exact *P*-values are provided in the Source Data file. Full statistical details are provided in the Methods.

Supplementary Table 1: Non-equilibrium kinetic parameters determined using constant speed AFM-SMFS. Error reported as standard error.

Protein Complex	Pathway	BE model		DHS model (υ = 1/2)			DHS model (υ = 2/3)		
		k_0 [s^{-1}]	Δx [nm]	k_0 [s^{-1}]	Δx [nm]	$\Delta G^{\ddagger}$ [k_BT]	k_0 [s^{-1}]	Δx [nm]	$\Delta G^{\ddagger}$ [k_BT]
Izumo1:Juno	0	1.28±2.56	0.779 ± 0.264	1.46 ± 0.95	0.681 ± 0.191	19.52 ± 4.38	0.92 ± 0.68	0.405 ± 0.136	15.14 ± 4.29
	1	3.62± 3.22	0.240 ± 0.039	4.66 ± 0.25	0.072 ± 0.006	13.89 ± 0.96	3.78 ± 0.24	0.032 ± 0.004	9.49± 2.22
	2	4.20± 0.37	0.046 ± 0.001	3.41 ± 0.19	0.039 ± 0.001	16.12 ± 0.37	2.36 ± 0.15	0.022 ± 0.001	11.75 ± 0.35
Izumo1E71A:Juno	0	1.27± 1.79	0.726 ± 0.178	2.43 ± 0.86	0.447 ± 0.082	17.28 ± 2.43	1.69 ± 0.64	0.252 ± 0.056	12.70 ± 2.26
	1	4.10±4.49	0.244 ± 0.047	3.34 ± 0.46	0.116 ± 0.012	15.13 ± 1.00	2.49 ± 0.38	0.061 ± 0.008	10.44 ± 0.92
	2	4.30± 0.67	0.045 ± 0.002	3.88 ± 0.18	0.035 ± 0.001	15.94 ± 0.38	2.76 ± 0.14	0.018 ± 0.001	11.35 ± 0.34
Izumo1K150A:Juno	0	1.24± 1.98	0.809 ± 0.226	1.84 ± 0.75	0.663 ± 0.123	18.07 ± 2.75	1.20 ± 0.52	0.387 ± 0.085	13.72 ± 2.53
	1	1.39± 0.57	0.227 ± 0.022	3.78 ± 0.58	0.097 ± 0.014	14.78 ± 1.23	2.00 ± 0.51	0.049 ± 0.010	9.78± 1.08
	2	2.55± 2.11	0.041 ± 0.01	3.66 ± 0.26	0.038 ± 0.002	16.16 ± 0.70	2.65 ± 0.27	0.020 ± 0.002	12.14 ± 1.61
Izumo1H157A:Juno	0	1.07± 3.5	0.937 ± 0.525	3.46 ± 0.68	0.401 ± 0.047	14.69 ± 1.53	2.57 ± 0.51	0.221 ± 0.029	10.07 ± 1.31
	1	4.34± 20.09	0.23± 0.192	4.01 ± 0.34	0.063 ± 0.006	14.66 ± 0.90	3.34 ± 0.32	0.030 ± 0.004	9.51± 1.08
	2	2.14± 0.20	0.036 ± 0.001	3.43 ± 0.23	0.040 ± 0.002	17.44 ± 0.62	2.30 ± 0.18	0.022 ± 0.001	12.85 ± 0.62
Izumo1R160A:Juno	0	1.14± 1.68	0.875 ± 0.222	3.06 ± 0.83	0.376 ± 0.074	14.74 ± 2.15	2.28 ± 0.66	0.198 ± 0.048	10.10 ± 1.94
	1	0.91± 0.21	0.169 ± 0.011	4.57 ± 0.32	0.067 ± 0.006	13.53 ± 0.63	3.70 ± 0.30	0.032 ± 0.004	8.64± 0.59
	2	2.54± 0.13	0.039 ± 0.001	3.03 ± 0.20	0.043 ± 0.002	16.72 ± 0.41	2.06 ± 0.17	0.024 ± 0.001	12.42 ± 0.39

Izumo1:JunoH177Q*	0	1.05± 1.66	0.956 ± 0.261	1.98 ± 0.91	0.752 ± 0.186	17.31 ± 3.15	1.30 ± 0.65	0.434 ± 0.127	13.01 ± 2.95
	1	1.98± 8.10	0.506 ± 0.361	2.21 ± 1.08	0.329 ± 0.076	17.77 ± 3.31	1.59 ± 0.82	0.183 ± 0.051	12.95 ± 3.06
	2	2.34± 3.46	0.167 ± 0.051	5.03 ± 0.28	0.048 ± 0.006	10.89 ± 0.62	4.18 ± 0.31	0.020 ± 0.004	6.12± 0.60

* During global fitting of the DHS model, forces greater than 400 pN were excluded as outliers that impacted convergence of the fitting algorithm.

Supplementary Table 2: Summary of the SMD simulations performed.

Protein complex	Replicas	Time [ns]	Box shape [Å]	Total atoms	Water molecules	[NaCl]
Izumo1:Juno	50	90	130x130x180 Å	~283,000	~92,000 (TIP3P model)	0.15 M
Izumo1:JunoH177Q	50	90	130x130x180 Å	~283,000	~92,000 (TIP3P model)	0.15 M
Izumo1:JunoH177E	50	90	130x130x180 Å	~283,000	~92,000 (TIP3P model)	0.15 M

Supplementary Table 3: Distribution of Izumo1:Juno residues between different interaction clusters. Positions of bolded residues can be seen in Fig. 2.

Cluster	Izumo1 residues	Juno residues
1	A158, C159, R160, K161, S162, Y163, D164<br><br>N239, S241, P242, A243	K42, Y44, E45<br><br>L58, T59, W62, H65, L66
2	Q65, S68, L69, E71, D72, A73, Y74, M75, P84<br><br>Y134<br><br>L146, W148, K150, N151, E155, H157	D67<br><br>F77, L81, M83, P84, R87<br><br>M145<br><br>Q161, R165, L168, Q171, L173, P174
3	K34, E37, K38<br><br>K48, H49, K51, M54, E55, R56, N59, K62, N63<br><br>D105, K109, F112, W113, H116, K119	Q133<br><br>W140, E141, R144, Y147<br><br>H177<br><br>T181

Supplementary Table 4: Kinetic parameters for unbinding pathway switching determined using Monte Carlo simulation of non-reversible transitions.

Protein complex	Pathway	BE model	
		k_0 [s^{-1}]	Δx [nm]
Izumo1:Juno	0 → 1	0.0002	5.28
	1 → 2	0.0008	3.05

Supplementary Table 5: Kinetic parameters for unbinding pathway switching determined using Monte Carlo simulation of reversible transitions.

Protein complex	Pathway	BE model	
		**k_0 [s^{-1}]**	**Δx [nm]**
Izumo1:Juno	0 → 1	0.0008	3.46
	1 → 0	41.0662	0.28
	1 → 2	0.8874	1.34
	2 → 1	129.2846	0.03

Supplementary References

1. Bianchi, E., Doe, B., Goulding, D. & Wright, G. J. Juno is the egg Izumo receptor and is essential for mammalian fertilization. *Nature* **508**, 483–487 (2014).
2. Aydin, H., Sultana, A., Li, S., Thavalingam, A. & Lee, J. E. Molecular architecture of the human sperm IZUMO1 and egg JUNO fertilization complex. *Nature* **534**, 562–565 (2016).
3. Hernández, J. M. & Podbilewicz, B. The hallmarks of cell-cell fusion. *Development* **144**, 4481–4495 (2017).
4. Brukman, N. G. *et al.* A novel function for the sperm adhesion protein IZUMO1 in cell-cell fusion. *J. Cell Biol.* **222**, (2023).
5. Pacak, P., Kluger, C. & Vogel, V. Molecular dynamics of JUNO-IZUMO1 complexation suggests biologically relevant mechanisms in fertilization. *Sci. Rep.* **13**, 20342 (2023).
6. Pierse, C. A. & Dudko, O. K. Distinguishing Signatures of Multipathway Conformational Transitions. *Phys. Rev. Lett.* **118**, 088101 (2017).
7. Scheurer, M. *et al.* PyContact: Rapid, Customizable, and Visual Analysis of Noncovalent Interactions in MD Simulations. *Biophys. J.* **114**, 577–583 (2018).
8. Stone, J. E. An efficient library for parallel ray tracing and animation. (Missouri University of Science and Technology, 1998).
9. Humphrey, W., Dalke, A. & Schulten, K. VMD: visual molecular dynamics. *J. Mol. Graph.* **14**, 33–8, 27–8 (1996).
