# Supplementary material for: Multi-state catch bond formed in the Izumo1:Juno complex that initiates human fertilization
Source: Nat Commun. 2025 Aug 26;16:7952. doi: 10.1038/s41467-025-62427-0 (PMC12381214; doi:10.1038/s41467-025-62427-0)
Supplement: Supplementary file 9 — Source data [file 41467_2025_62427_MOESM9_ESM.zip › Source Data Zip/MD configuration file annealing.pdf]

```
cutoff 12.0
pairlistdist 14.0
switching on
switchdist 10.0
PME on
PMEGridspacing 1
wrapAll on
wrapWater on
###########################################################################
#
#cr
#cr            (C) Copyright 1995-2009 The Board of Trustees of the
#cr                        University of Illinois
#cr                         All Rights Reserved
#cr
###########################################################################
#

###########################################################################
#
# RCS INFORMATION:
#
#       $RCSfile: Annealing.conf,v $
#       $Author: jribeiro $        $Locker:  $             $State: Exp $
#       $Revision: 1.2 $       $Date: 2017/05/10 19:03:08 $
#
###########################################################################
#
##START HERE###
##Simulation Template##
# Simulation conditions
coordinates smd_QwikMD.pdb
structure smd_QwikMD.psf

binCoordinates Minimization.restart.coor
binVelocities  Minimization.restart.vel
extendedSystem Minimization.restart.xsc

# Simulation conditions
#temperature 60


# Harmonic constraints

constraints on
consref Minimization_restraints.pdb
conskfile Minimization_restraints.pdb
constraintScaling 2
consexp 2
conskcol B


# Output Parameters

binaryoutput no
```

```
outputname Annealing
outputenergies 40
outputtiming 40
outputpressure 40
binaryrestart yes
dcdfile Annealing.dcd
dcdfreq 1000
XSTFreq 1000
restartfreq 1000
restartname Annealing.restart


# Thermostat Parameters
langevin on
langevintemp 60
langevinHydrogen    off
langevindamping 1

# Barostat Parameters


langevinpiston on
usegrouppressure yes
useflexiblecell no
useConstantArea no
langevinpistontarget 1.01325
langevinpistonperiod 200
langevinpistondecay 100
langevinpistontemp 60

# Integrator Parameters

timestep 2
firstTimestep 0
fullElectFrequency 2
nonbondedfreq 1
#stepspercycle 10

# Force Field Parameters

paratypecharmm on
parameters toppar_water_ions_namd.str
parameters toppar_all36_carb_glycopeptide.str
parameters par_all36_lipid.prm
parameters par_all36_na.prm
parameters par_all36_prot.prm
parameters par_all36_carb.prm
parameters par_all36_cgenff.prm
exclude scaled1-4
1-4scaling 1.0
rigidbonds all


#Implicit Solvent Parameters

gbis off
```

```
alphaCutoff         14.0
ionConcentration    0.15

# Script
set Temp 310
set barostat 1
set nSteps 580
for {set t 60} {$t <= $Temp} {incr t} {run $nSteps;langevintemp $t;if
{$barostat} {langevinpistontemp $t}}

set file [open Annealing.check w+]
set done 1
if {[file exists Annealing.restart.coor] != 1 || [file exists
Annealing.restart.vel] != 1 || [file exists Annealing.restart.xsc] != 1 } {
  set done 0
}
if {$done == 1} {
 puts $file "DONE"
    flush $file
  close $file
} else {
 puts $file "One or more files failed to be written"
   flush $file
  close $file
}
```
