# Supplementary material for: Multi-state catch bond formed in the Izumo1:Juno complex that initiates human fertilization
Source: Nat Commun. 2025 Aug 26;16:7952. doi: 10.1038/s41467-025-62427-0 (PMC12381214; doi:10.1038/s41467-025-62427-0)
Supplement: Supplementary file 9 — Source data [file 41467_2025_62427_MOESM9_ESM.zip › Source Data Zip/MD configuration file equilibration.pdf]

```
cutoff 12.0
pairlistdist 14.0
switching on
switchdist 10.0
PME on
PMEGridspacing 1
wrapAll on
wrapWater on
############################################################################
#
#cr
#cr            (C) Copyright 1995-2009 The Board of Trustees of the
#cr                        University of Illinois
#cr                         All Rights Reserved
#cr
############################################################################
#

############################################################################
#
# RCS INFORMATION:
#
#       $RCSfile: Equilibration.conf,v $
#       $Author: jribeiro $        $Locker:  $             $State: Exp $
#       $Revision: 1.2 $       $Date: 2017/05/10 19:03:08 $
#
############################################################################
#
##START HERE###
##Simulation Template##
# Simulation conditions
coordinates smd_QwikMD.pdb
structure smd_QwikMD.psf

binCoordinates Annealing.restart.coor
binVelocities Annealing.restart.vel
extendedSystem Annealing.restart.xsc

# Simulation conditions
#temperature 60


# Harmonic constraints

constraints on
consref Minimization_restraints.pdb
conskfile Minimization_restraints.pdb
constraintScaling 2
consexp 2
conskcol B


# Output Parameters

binaryoutput no
```

```
outputname Equilibration
outputenergies 500
outputtiming 500
outputpressure 500
binaryrestart yes
dcdfile Equilibration.dcd
dcdfreq 5000
XSTFreq 5000
restartfreq 5000
restartname Equilibration.restart


# Thermostat Parameters
langevin on
langevintemp 310
langevinHydrogen    off
langevindamping 1

# Barostat Parameters


langevinpiston on
usegrouppressure yes
useflexiblecell no
useConstantArea no
langevinpistontarget 1.01325
langevinpistonperiod 200
langevinpistondecay 100
langevinpistontemp 310

# Integrator Parameters

timestep 2
firstTimestep 0
fullElectFrequency 2
nonbondedfreq 1
#stepspercycle 10

# Force Field Parameters

paratypecharmm on
parameters toppar_water_ions_namd.str
parameters toppar_all36_carb_glycopeptide.str
parameters par_all36_lipid.prm
parameters par_all36_na.prm
parameters par_all36_prot.prm
parameters par_all36_carb.prm
parameters par_all36_cgenff.prm
exclude scaled1-4
1-4scaling 1.0
rigidbonds all


#Implicit Solvent Parameters

gbis off
```

```
alphaCutoff          14.0
ionConcentration     0.15

run 500000

set file [open Equilibration.check w+]
set done 1
if {[file exists Equilibration.restart.coor] != 1 || [file exists
Equilibration.restart.vel] != 1 || [file exists Equilibration.restart.xsc]
!= 1 } {
  set done 0
}
if {$done == 1} {
 puts $file "DONE"
    flush $file
  close $file
} else {
 puts $file "One or more files failed to be written"
   flush $file
  close $file
}
```
