# Supplementary material for: Multi-state catch bond formed in the Izumo1:Juno complex that initiates human fertilization
Source: Nat Commun. 2025 Aug 26;16:7952. doi: 10.1038/s41467-025-62427-0 (PMC12381214; doi:10.1038/s41467-025-62427-0)
Supplement: Supplementary file 8 — Transparent Peer Review file [file 41467_2025_62427_MOESM8_ESM.pdf]

nature portfolio

Peer Review File

Force-dependent Reorganization and Mechanostability of the Izumo1:Juno Complex Involved in Human Fertilization

Corresponding Author: Professor Michael Nash

This file contains all reviewer reports in order by version, followed by all author rebuttals in order by version.

Version 0:

Reviewer comments:

Reviewer #1

(Remarks to the Author)
In this manuscript, the authors investigate the mechanical behavior of the Izumo1:Juno protein complex, which plays a crucial role in human fertilization by mediating sperm-egg adhesion. Using atomic force microscopy-based single-molecule force spectroscopy (AFM-SMFS), Monte Carlo modeling, and all-atom steered molecular dynamics (SMD) simulations, the authors identified three distinct unbinding pathways of the Izumo1:Juno complex. These pathways give rise to a multi-state catch bond mechanism. Furthermore, the authors explore the implications of a mutation in Juno (JunoH177Q), which is associated with infertility. This mutation does not affect the equilibrium binding affinity but is implicated in the proposed high-force stabilization mechanism. This finding highlights the critical role of the Izumo1:Juno complex's mechanostability in fertility, and suggests possible targets for drug development. The experimental and computational methods are sound and appropriate to mechanically characterize the system. The manuscript is well structured and clear. The data analysis is quite confusing as described below. The work would be of interest to the broad readership of Nature communications. We have some comments that would help improve the readability and enhance the impact of the manuscript and that should be addressed before acceptance.
Comments and suggestions:
1. Regarding the fits to the experimental data, if I understand well, the authors first derive the koff(F) (Eq. 4) from the rupture force histograms at each velocity for then fitting the DHS model. For the BE model analysis, they directly fit the rupture forces versus loading rates. Finally, they use the BE model results to carry out the MC simulations, while plotting the DHS results in Fig. 1. Overall, the reasons behind using these different approaches/models are difficult to understand and the overall strategy is not clear. Why plotting DHS in Fig. 1 (and equivalent figures) while using BE for the MC simulations? Importantly, the fits in Fig. 1 (and in S14, S15 and S17) do not seem to follow the median rupture forces, which suggests that the chosen model or the resulting fits are not appropriate. This may be due to the spreading of the rupture force distributions due to experimental uncertainties, which may induce bias of the DHS fit. Besides, the experimental koff derived from the rupture force histograms should be shown together with the fits (Fig. S3). If possible, I would suggest using just one model (BE or DHS). This would simplify the analysis and interpretation of the results. In any case, the fitting procedure should be better explained.
2. Related to the previous comment, the Monte Carlo simulation strategy is not clear either and I am not sure if it provides insightful information. If I understand well, the constant speed MC results are used to fit the experimental data and extract the energy landscape parameters to confront the fitted model parameters. What was the outcome of these simulations? The fitter parameters used? On the other hand, in the methods, the authors mention that they use the experimentally derived BE model parameters to feed the MC simulations (line 389ss in SI), while they also mention the DHS model for the MC fits (SI, lines 279ss). Again, this is not clear and should be better explained and the underlying rationale justified. If possible, a simplified strategy should be used.
3. Regarding the force clamp MC simulations, the authors use these results to propose the most probable pathway at the different force regimes. However, they do not explain how they extract this information from the MC simulations. Also, how where the reversible rates determined?
4. The details of the statistical test should be provided. For example, it would be useful to clearly state the H0 hypothesis (as well as the H1 hypothesis) of each statistical test. These hypotheses form the foundation for the test and help the reader to define the context for evaluating the data. (Figure.1, line 105)
5. Regarding the SMD simulations, it would be useful to give more details about the steps before pulling: minimization and equilibration; such as the total number of steps used to minimize and total time in nanoseconds to equilibrate.
6. The authors mention that the Izumo1 histidine H157 is highly conserved in Supp. data (page 19 line 573). It is known that

highly conserved residues are often involved in maintaining the protein's three-dimensional structure. Histidine, particularly in its protonated form, can participate in hydrogen bonds that contribute to stabilizing protein structure (which the authors mention in Supp.data). Did the authors consider the potential impact of mutating this conserved histidine on the protein's structure, as such mutation could have an impact on the 3D structure leading to misfolding ? Typically by first predicting the structure of the mutant using Alpha Fold then comparing the WildType structure to the mutant structure (RMSD shows the differences between the two structures). This also applies for the other mutation R160A.
7. The authors claim that "the combined distance intervals [...] in SMD are consistent with the intermediate unfolding events observed experimentally" (lines 219ss). However, the contour length increments in SMD ranged from 5.7 nm to 7.7 nm (lines 214ss), while most of the experimental increments ranged from 10 nm to 30 nm (Fig. S4). This is difficult to justify and may be due to the large gap between experimental and pulling velocities. This should be mentioned.
8. In Fig. S2, pathways are named a, b and c instead of P0, P1 and P2 (I guess).
9. In Fig. S3, experimental data should be shown.
10. Fig. S19G is totally blank. Is this on purpose? If so, I don't see the point in showing a blank plot.
11. What is the difference between Fig. 5C and Figs. S23 F-H? Are they different datasets? It is not clear from the text. Why not merge the different datasets to increase the statistical power?
12. The pdb codes of the proteins and complexes used in the simulations should be provided.
13. SI line 298 "where l is (ln) loading rate." Is "l" really "(ln) loading rate"?

Reviewer #2

(Remarks to the Author)
I co-reviewed this manuscript with one of the reviewers who provided the listed reports. This is part of the Nature Communications initiative to facilitate training in peer review and to provide appropriate recognition for Early Career Researchers who co-review manuscripts.

Reviewer #3

(Remarks to the Author)
This is a very interesting study of the interactions between the two key proteins involved in the process of fertilization, namely Izumo1 (from the sperm cell) and Juno (expressed on the egg cell surface.) The work is performed primarily at the single-molecule level using a powerful AFM-based single-molecule force spectroscopy, which brings the two proteins together and then directly measures the force necessary to separate them (detachment or rupture force). SMFS measurements are accompanied by all-atom steered molecular dynamics simulations, essentially modeling the AFM experiments and allowing the structural insights under force, at the atomic level. In addition, an infertility postulated mutation is engineered in Juno and its effects on the mechanostablity of the Izumo1-Juno complex are probed by experimental SMFS and computationally by SMD and Monte Carlo simulations. The main conclusion of this work includes identifying a new mechanism supporting the enhancement of the mechanical stability of the Izumo1-Juno interactions through so-called catch-bonds, which are bonds that counter-intuitively are stronger under force or are established under force. Another important conclusion is that the infertility-causing H177Q mutation in Juno adversely affects these catch bonds and overall weakens Izumo1-Juno interactions. The experimental and computational procedures are executed expertly, however, their description, particularly in the Supporting Information reveals some issues and inconsistencies that need to be improved/corrected.
Below I list my suggestions and concerns:
Main text:
1. Please explain briefly the fingerprinting role of ddFLN4 in the main text and check the current description in the SI. Not everyone knows/remembers that two force peaks are expected for each ddFLN4 module, producing (mainly) a four-peak SMFS fingerprint.

2. P. 5 "For both DHS and BE fitting of P0, P1, and P2 rupture events, we observed a several fold decrease in Δx "
Δx is not defined in the main text up to this point. One can guess it is the distance to the unbinding transition state, but it should be clearly defined as such in the text. Also, in the SI, Δx has a double dagger, which is not used in the main text.
3. p. 6 "Across the constant speed AFM-SMFS Izumo1:Juno datasets, we also identified the presence of several intermediate Izumo1:Juno folded states. To further explore these observations and their relationship to P0, P1, and P2, we subdivided the P0, P1, and P2 force vs. extension curves based on the presence of these intermediates (Fig. 1D)".

Please explain how these intermediates manifest themselves and are identified in the force-extension curves.

Supporting Information:
1. p. 7, "In the final modification step, the ybbR-tags located at the C-terminus of the target proteins…"
What is meant by the target protein here? The constructs and sequences involving ybbR tags shown on pp. 4 and 5 indicate the N-terminal location of ybbR not C-terminal.

2. Supp Info, p.8 "Boltzman", please correct spelling.

3. The authors choose the Freely-Rotating-Chain (FRC) model for their molecular system to analyze force-extension data and to determine the contour length of their constructs, from the F-x data.

Please provide some rationale for choosing this model over other approaches. Can the segment elasticity (bond-angle bending) be neglected at the forces explored in this work?

On page 8, the authors present various approximations to the FRC model to calculate the contour length L, following reference 52. Please review this section carefully as these equations are not copied/adapted correctly from reference 52. Please introduce the necessary definitions for the variable used, and check that they are consistently used.
Why would one like to calculate the bond length "b" using the Kuhn length "a" (eq. 2)? Also, "l" is the persistence length and not "p" in those equations.

4. p.8 "Under the first selection criteria, single molecule interactions between Izumo1 and Juno were identified as individual rupture events < 100 pN at contour lengths of ~135 nm This criterion was based on FRC-fitting of the expected contour length the polypeptide chains for this force range.

The expected contour length of ~135 nm, needs to be carefully accounted for considering that the pulling handles have a complex composition involving unstructured ELPs and structured Spytag-Spycatcher modules that potentially may unfold in these measurements, in addition to the ddFLN4 domains. If this unfolding is prevented by the specific orientation of the SpyCatcher domains in the constructs, this should be explained and described in SI.

Reviewer #4

(Remarks to the Author)
Izumo1:Juno mediate the adhesion between sperm and egg cells. This manuscript explores the role of mechanical forces in regulating the human Izumo1:Juno complex. While this study provides important insights into the mechanical underpinnings that regulate basis of human fertilization, there are numerous issues that need to be addressed before publication.

Major Concerns
1. The manuscript is incredibly difficult to read and digest. Essential details required to interpret the experiments and simulations are missing from the manuscript. Key details and interpretations/discussion are buried a >60 page SI. Prior to publication, this manuscript will need to be completely overhauled.

2. While measuring catch bonds from AFM force clamp measurements are intuitive and rigorous, interpreting catch bonds from AFM single molecule force spectroscopy (SMFS) is fraught since interpretations are completely model dependent. Puzzlingly, while the authors invest so much effort in performing SMFS experiments (that are riddled with artifacts), they perform the force clamp measurements in an extremely superficial fashion (i.e. only Fig 3D). The force-clamp data is also described very cursorily. I would recommend that the authors perform force clamp measurements in a more rigorous and systematic manner and describe these measurements in far greater detail.

3. It is unclear how the authors decided from the SMFS experiments that Izumo1:Juno predominantly unbind along three distinct pathways. This is a critical point in this paper and is poorly justified. It appears that the authors determined multiple unbinding pathways solely using a Gaussian Mixture Model which is not convincing. At the very least, the authors need to provide orthogonal experimental evidence using a different experimental technique to justify their model.

4. It is also unclear how the authors determined from their AFM data that the P0 pathway involves no intermediate states while the P1 and P2 pathways involve intermediate states. It appears that the only justification for this were the small unbinding peaks in the SMFS force curves which is a wholly insufficient justification since it could be an artifact of surface functionalization or due to multiple unbinding events. Several additional controls and orthogonal experiments are needed to substantiate this claim.

5. In the Izumo1:Juno rupture force vs. loading rate plots, the P1 and P2 experimental data (purple and cyan in Fig 1F) do not fit to the DHS model. How do the authors rationalize these poor fits?

6. The authors should provide the probability of single and multiple unbinding events. These probabilities are needed to determine if the unbinding event rate follows a Poisson distribution in order to determine the likelihood that they are indeed single molecule unbinding events.

7. Are the 'histograms' in Figure 1D, conventional histograms or are they kernel density estimators? Some of these histograms only have 22 events (for instance P1a) and seem remarkably normally distributed for such a low event rate. Also why do the authors refer to these as "force vs. contour length histograms"? These just seem like 1D histograms of the contour length (and not 2D force vs. contour length histograms). The authors need to be more precise in the language they use throughout the manuscript.

8. Several critical controls are lacking. For instance, how did the authors rule-out that P1 and P2 are separate pathways and do not merely arise from unbinding of multiple Izumo1:Juno molecules?

9. The SMD simulations performed with the JunoH177Q mutation are intriguing. I believe that further molecular details can be obtained from these simulations by performing additional analysis. For instance, the authors only analyze the contact area between Izumo and Juno (which I assume is the Solvent Accessible Surface Area, although this is never specified). However, I think they should also monitor the formation of hydrogen bonds and other electrostatic interactions that are

abolished by the JunoH177Q mutation. For instance, in order to conclude that the increased mechanostability of JunoH177E is charge related, the authors should monitor salt-bridge formation across the simulation frames

Minor Concern
1. The statement on page 3 that “presence of C-terminal SpyTags were used to confirm the successful expression and purification of Izumo1 and Juno” is vague. The authors need to explicitly state what they mean by this statement.

Version 1:

Reviewer comments:

Reviewer #1

(Remarks to the Author)
The authors have addressed most of our comments and the manuscript is now easier to follow. However, the answer to my comment states that “We used the DHS model for directly fitting the rupture force vs. loading rate plots”, while, in the methods it is mentioned that the k_off(F) plots derived from the force distributions were used to fit the DHS model. This is contradictory to me, and I guess that it is mistake in the answer.
In any case, I think that the main problem comes from the fact that the authors do not show any of the fits of the k_off(F) plots derived from the force distribution, but the final predictions (not fits, as stated in line 328) of the DHS parameters in the force vs loading rate plot. I think that including the experimental plots of k_off(F) from the distribution with the corresponding fits used to extract the DHS parameters would importantly help understanding all the data analysis process. Perhaps a pipeline showing the data processing and analysis will help, too.
Besides, I insist that reporting a blank plot is nonsense and leads to confusion (Fig. S19G). I would rather remove it and mention that it is missing in the figure caption due to the lack of data.
After addressing these points, the manuscript will be suitable for publication.

Reviewer #2

(Remarks to the Author)
I co-reviewed this manuscript with one of the reviewers who provided the listed reports. This is part of the Nature Communications initiative to facilitate training in peer review and to provide appropriate recognition for Early Career Researchers who co-review manuscripts.

Reviewer #3

(Remarks to the Author)
I am satisfied with the Authors' responses to my questions and by their corrections of identified errors.
I have no further questions/suggestions.

Reviewer #4

(Remarks to the Author)
Most of my concerns arose from essential details that were missing in the original submission. The changes the authors have incorporated in their revision, and the additional analysis they have performed, ally these concerns.

Open Access This Peer Review File is licensed under a Creative Commons Attribution 4.0 International License, which permits use, sharing, adaptation, distribution and reproduction in any medium or format, as long as you give appropriate credit to the original author(s) and the source, provide a link to the Creative Commons license, and indicate if changes were made.
In cases where reviewers are anonymous, credit should be given to 'Anonymous Referee' and the source.
The images or other third party material in this Peer Review File are included in the article's Creative Commons license, unless indicated otherwise in a credit line to the material. If material is not included in the article's Creative Commons license and your intended use is not permitted by statutory regulation or exceeds the permitted use, you will need to obtain permission directly from the copyright holder.
To view a copy of this license, visit https://creativecommons.org/licenses/by/4.0/

Reviewer #1 (Remarks to the Author):

"*In this manuscript, the authors investigate the mechanical behavior of the Izumo1:Juno protein complex, which plays a crucial role in human fertilization by mediating sperm-egg adhesion. Using atomic force microscopy-based single-molecule force spectroscopy (AFM-SMFS), Monte Carlo modeling, and all-atom steered molecular dynamics (SMD) simulations, the authors identified three distinct unbinding pathways of the Izumo1:Juno complex. These pathways give rise to a multi-state catch bond mechanism. Furthermore, the authors explore the implications of a mutation in Juno (JunoH177Q), which is associated with infertility. This mutation does not affect the equilibrium binding affinity but is implicated in the proposed high-force stabilization mechanism. This finding highlights the critical role of the Izumo1:Juno complex's mechanostability in fertility, and suggests possible targets for drug development. The experimental and computational methods are sound and appropriate to mechanically characterize the system. The manuscript is well structured and clear. The data analysis is quite confusing as described below. The work would be of interest to the broad readership of Nature communications. We have some comments that would help improve the readability and enhance the impact of the manuscript and that should be addressed before acceptance.*"

We thank the reviewers for the positive feedback.

Comments and suggestions:

1. *"Regarding the fits to the experimental data, if I understand well, the authors first derive the koff(F) (Eq. 4) from the rupture force histograms at each velocity for then fitting the DHS model. For the BE model analysis, they directly fit the rupture forces versus loading rates. Finally, they use the BE model results to carry out the MC simulations, while plotting the DHS results in Fig. 1. Overall, the reasons behind using these different approaches/models are difficult to understand and the overall strategy is not clear. Why plotting DHS in Fig. 1 (and equivalent figures) while using BE for the MC simulations? Importantly, the fits in Fig. 1 (and in S14, S15 and S17) do not seem to follow the median rupture forces, which suggests that the chosen model or the resulting fits are not appropriate. This may be due to the spreading of the rupture force distributions due to experimental uncertainties, which may induce bias of the DHS fit. Besides, the experimental koff derived from the rupture force histograms should be shown together with the fits (Fig. S3). If possible, I would suggest using just one model (BE or DHS). This would simplify the analysis and interpretation of the results. In any case, the fitting procedure should be better explained."*

We used the DHS model for directly fitting the rupture force *vs.* loading rate plots. The BE model was used for Monte Carlo (MC) force spectroscopy simulations and fitting the rate constants for transition between the intermediate states. These choices were made based on a balance of physical realism and computational feasibility.

Briefly, the DHS model provides a more nuanced interpretation of the shape of the energy landscape by explicitly incorporating the effects of force on Δx. This provides a non-linear response of the model and makes the DHS model well-suited for fitting experimental rupture force distributions. In our experience, we very often see non-linearities in rupture force vs. loading rate data, especially when covering a wide range of loading rates. The DHS model allows us to capture some of this behavior, albeit in a somewhat rudimentary framework. We also like being able to extract the $\Delta G^{\ddagger}$ (activation barrier height) parameter because it allows an additional point of comparison between different systems (e.g., different pathways, mutational variants, etc…). However, an inherent limitation of the DHS model is the occurrence of a singularity or critical force, at which point the model predicts that the energy barrier vanishes. Beyond this critical force, the

model predicts non-physical behaviors such as imaginary or negative rates and it ceases to be valid. The model assumes thermally activated barrier crossing, but in the absence of a barrier it becomes invalid. Quantitatively, this occurs when the dimensionless number $F \cdot \Delta x / \Delta G^{\ddagger} \geq 1$. Due to this numerical instability, when we use the DHS rate expression in numerical Monte Carlo simulations, we encounter instabilities, stalled simulation runs and other problems. Based on this experience, we find it is much simpler to use the BE model for numerical MC simulations in these systems. However, when fitting experimental data directly (as in Figure 1), we find that the DHS model is useful and provides better fits.

We also believe there is value in presenting the kinetic parameters derived from both models. It provides a degree of cross-validation of the theoretical analyses in the presence of different underlying assumptions regarding Δx. Since our MC force spectroscopy simulations involve millions of iterations, we opted for the BE model, which is computationally efficient and avoids the numerical instabilities of the DHS model, while still capturing the essential biophysics and multi-state kinetic pathways of force-dependent Izumo1:Juno unbinding (*i.e*. the relative behavior of Δx and k_0 for each unbinding pathway).

Regarding the difference between the median rupture forces and the DHS model fits: Due to the nature of how the DHS model is fit, we do not expect them to closely follow the median rupture force-median loading rate coordinates in the same way the BE model typically does. As the reviewer correctly pointed out, this is because the DHS model fits the full shape of the rupture force probability distribution for each pathway, rather than just taking the median rupture force values.

In response to this comment we made the following revisions:

1. We introduce the DHS and BE models together by name instead of just the BE model and referring to the DHS as an extension. This is to improve clarity and to show the relationship between the two models (**p.5**)
2. We explain the key difference between the DHS and BE models in the main text (**p.5**).
3. We provide justification for the use of BE kinetic parameters in MC simulations due to numerical constraints in the main text (**p.10**) and SI (**p.15**).
4. We reviewed the Monte Carlo force spectroscopy section in the main text providing additional detail to improve clarity (**p.10-11**).
5. We state the value in comparing both the DHS and BE models when analyzing energy landscapes in the main text (**p.6**).
6. We explicitly state that the median rupture forces-median loading rates coordinates are not necessarily meant to be congruent to the DHS fit due to the nature of how the DHS model is fit and what it represents in a complex multi-state system (**SI p.10**).
7. As requested, we included the fitted k_0, Δx, and $\Delta G^{\ddagger}$ values within this figure showing the calculated force-dependent off rates (**fig.S3**, **SI p.28**).
8. We reviewed and provided additional clarification on the DHS fitting procedure described in the SI (**p.10-11**).

2. *"Related to the previous comment, the Monte Carlo simulation strategy is not clear either and I am not sure if it provides insightful information. If I understand well, the constant speed MC results are used to fit*

the experimental data and extract the energy landscape parameters to confront the fitted model parameters. What was the outcome of these simulations? The fitter parameters used? On the other hand, in the methods, the authors mention that they use the experimentally derived BE model parameters to feed the MC simulations (line 389ss in SI), while they also mention the DHS model for the MC fits (SI, lines 279ss). Again, this is not clear and should be better explained and the underlying rationale justified. If possible, a simplified strategy should be used."

We appreciate the reviewer's questions and recognize the need for further clarity. The experimental data in Fig. 1 was fit with both the BE and DHS models. However, only the BE parameters were then used in the Monte Carlo force spectroscopy simulations. To test and fit the "invisible" inter-state transition rates, we generated millions of simulated rupture force distributions using randomly sampled Δx and k_0 values. We then selected the inter-state transition parameter set with the lowest residual sum of squares for the simulated rupture force distributions when compared to the experimental rupture force distribution. We further used this difference minimization approach to select the catch bond model (reversible or irreversible) that was more representative of the experimental data.

To fit the DHS model to the experimental constant-speed AFM-SMFS data, an enhanced Monte Carlo (random) sampling strategy was used to test a broad range of initialization parameters to locate the global minima. This fitting approach is unrelated to the Monte Carlo force spectroscopy simulations.

In response to this comment, we made the following revisions:

1. We reviewed and simplified the description of the DHS fitting procedure in the SI (**p.10-11**). As part of these changes we avoid the term Monte Carlo, instead describing it in terms of random sampling of initialization parameters.
2. Where needed, we provided additional clarification to all uses of the term "Monte Carlo" throughout the manuscript (*i.e.* whether they are Monte Carlo constant speed force spectroscopy or Monte Carlo force clamp force spectroscopy simulations.
3. Regarding the outcome of the Monte Carlo force spectroscopy simulations, we have carefully revised the main text MC force spectroscopy simulation section to explicitly state how these MC force spectroscopy simulations provide unique insights into the Izumo1:Juno system (*i.e.* the evaluation of the experimentally invisible barriers separating the native and intermediate states, **p.10**) and how they serve to help validate experimental interpretations (*i.e*. the presence of three-unbinding pathways) through cross-validation across orthogonal experimental force spectroscopy modalities and different types of MC force spectroscopy simulations, **p.12**).

3. "*Regarding the force clamp MC simulations, the authors use these results to propose the most probable pathway at the different force regimes. However, they do not explain how they extract this information from the MC simulations. Also, how where the reversible rates determined?*"

The most frequent rupture pathways were determined from the ratio of the total unbinding events through P0, P1, or P2 at each force set point. For example, if we performed 100 simulations at a given force set point, and 70 unbind through P0, 20 unbind through P1, and 10 unbind through P2, we conclude that for that particular force set point, P0 is the most frequent unbinding pathway.

The reversible rates were fit using iterative constant speed MC force spectroscopy simulations where the BE parameters for the rupture events (P0, P1, P2) were taken from the experimentally measured events and fed into the model. We then use algorithmic optimization of the reversible (invisible) rates between the various states. This allowed us to fit the experimentally invisible kinetic barriers separating N, I_1, and I_2.

In response to this comment, we made the following revisions:

1. We have changed our word choice in the main text to better reflect that this is a frequency comparison *i.e.* we describe it "as the most frequent unbinding pathway at rupture" (first paragraph, **p.11**).
2. Similarly, we updated our word choice in the figure legends of **Fig. 3** (**p.12**) and **fig. S23** (**SI p.55**) that describes how colour intensity reflects unbinding pathway frequency in our Monte Carlo force clamp simulation plots.
3. We have also explicitly stated how unbinding pathway frequency can be calculated from the MC force clamp simulations in the SI (**p.16**).
4. As part of our response to Reviewer #1 comment 2, we take care to specifically state the reversible kinetic parameters as an output of our Monte Carlo force spectroscopy simulation approach (**p.10**).

4. "*The details of the statistical test should be provided. For example, it would be useful to clearly state the H0 hypothesis (as well as the H1 hypothesis) of each statistical test. These hypotheses form the foundation for the test and help the reader to define the context for evaluating the data. (Figure.1, line 105)*"

We thank the reviewers for highlighting this omission.

In response to this comment we made the following revisions:

1. We have provided a detailed explanation of what constitutes the H0 and H1 hypothesis in each instance of statistical hypothesis testing used in this study (**SI p.16-17**).
2. We added comments throughout the manuscript directing readers to the statistical analysis section for further details where required.

5. "*Regarding the SMD simulations, it would be useful to give more details about the steps before pulling: minimization and equilibration; such as the total number of steps used to minimize and total time in nanoseconds to equilibrate.*"

The Izumo1:Juno structure was placed in a cubic box, neutralized and solvated in a physiological NaCl salt concentration of 0.15 M and TIP3 water molecules using 2 fs timesteps. Preparation of the system prior to pulling involved: minimization over 2000 steps, heating between 0 and 310 K over 145,000 steps, and equilibration (with backbone restraints of 2 kcal $\cdot$mol^{-1} $\cdot$Å^{-2}) for 10 ns. The Piz Daint supercomputer from the Swiss National Supercomputing Center in Lugano, Switzerland (CSCS) was used in all instances. It it worth noting the systems were periviously equilibrated for their structural features and stability, and this equilibration process here was only to ensure right solvent distribution in a new larger box compared to equilibrium simulations published previously.

In response to this comment we made the following revision:

1. We have added the number of minimization steps and total equilibration time to the description of the molecular dynamics set up (SI. p16).

6. "*The authors mention that the Izumo1 histidine H157 is highly conserved in Supp. data (page 19 line 573). It is known that highly conserved residues are often involved in maintaining the protein's three-dimensional structure. Histidine, particularly in its protonated form, can participate in hydrogen bonds that contribute to stabilizing protein structure (which the authors mention in Supp.data). Did the authors consider the potential impact of mutating this conserved histidine on the protein's structure, as such mutation could have an impact on the 3D structure leading to misfolding ? Typically by first predicting the structure of the mutant using Alpha Fold then comparing the WildType structure to the mutant structure (RMSD shows the differences between the two structures). This also applies for the other mutation R160A.*"

We appreciate the reviewer's comment on the potential structural impact of mutating H157 and R160 in Izumo1 to alanine. While we know that AlphaFold is powerful for global structure prediction, we don't think it is fully validated for accurate modeling of single-point mutations and their effects on local stability. We therefore did not study the suggested mutations with AlphaFold. However, our experimental binding affinity data confirmed binding activity, which would not be possible if Izumo1 were significantly misfolded.

7. "*The authors claim that "the combined distance intervals [...] in SMD are consistent with the intermediate unfolding events observed experimentally" (lines 219ss). However, the contour length increments in SMD ranged from 5.7 nm to 7.7 nm (lines 214ss), while most of the experimental increments ranged from 10 nm to 30 nm (Fig. S4). This is difficult to justify and may be due to the large gap between experimental and pulling velocities. This should be mentioned.*"

We agree with the reviewer that differences in the pulling velocities and timescales between AFM-SMFS experiments and steered molecular dynamics (SMD) simulations can influence the observed contour length increments. However, the length changes observed in the SMD are longer than what is cited by the reviewer. Our SMD showed median distance changes between the C-termini of Izumo1 and Juno of 5.7 ± 1.6 and 6.4 ± 0.9 nm for two-state and three-state unbinding trajectories. Additionally, 4% of these simulations showed partial unfolding of the Juno C-terminal alpha helix. We do not consider the percent of trajectories from the SMD showing the C-terminal Juno alpha helix unfolding as quantitatively predictive of what we can expect from experiments due to large differences in pulling speeds between experiment and SMD and the different loading rate dependencies of the various events (*i.e.* loading rate dependency of alpha helix unfolding vs. P0, P1 and P2 pathways). In the experimental datasets, we observe an intermediate unfolding event ~14-70% of the time, depending on the pathway and pulling speed. The fact that we observe C-terminal Juno alpha helix unfolding in SMD simulations at all is a sign that it is a likely populated pathway in our experiments, and most likely occurs more frequently than in the SMD simulations. This C-terminal Juno alpha helix unfolding event adds an additional 7.7 nm contour length to the system from SMD. Therefore, we felt it is reasonable to report experimentally observed Izumo1:Juno folded states (14.8-19.2 nm, fig. S4) as being roughly consistent with the combined median distance intervals seen in all-atom SMD, which includes contributions from the binding site rearrangements and the C-terminal Juno alpha helix unfolding, totalling 13.4-14.1 nm. Admittedly, this consistency relies on some speculation on our part that the C-terminal Juno helix unfolding that we observe in 4% of the SMD traces is a more frequently populated pathway (14-70%) in our experiments.

In response to this comment, we made the following revisions:

1. We added a brief discussion of how SMD pulling velocities could limit the observation of Juno unfolding or other intermediates (**p.9**).
2. We have directly provided the combined SMD Izumo1:Juno reorganization and Juno-unfolding contour length increments for easier comparison to the median experimental values (**p.9**).

8. "*In Fig. S2, pathways are named a, b and c instead of P0, P1 and P2 (I guess).*"

Curve subtypes a, b, and c as referred to in Fig. S2 are not the same as P0, P1, and P2. Subclass 'a' refers to curves lacking ddFLN4 unfolding. Subclass 'b' refers to curves with 1 ddFLN4 fingerprint domain, while subclass 'c' includes curves with ddFLN4 unfolding and Izumo1-Juno intermediate unfolding events. These details are provided in the caption. The subtype classifications of the representative force vs. extension curves provided in **fig. S2** are therefore correctly labeled.

To clarify this, we made the following revisions:

1. We have added a point of clarification regarding force vs. extension curve subtype classification and rupture pathway classification in the fig. S2 legend (**SI p.27**).
2. In describing how the intermediate states manifest in the force *vs*. extension curves in the main text, as requested by reviewer 3, we also include a reference to the subtype classification scheme examples in **fig. S2** (**p.6**)

9. "*In Fig. S3, experimental data should be shown.*"

We would like to clarify that **fig. S3** does not contain direct fits of the experimental data sets from **Fig. 1**, but rather illustrations of the calculated force-dependent off rates based on the experimentally derived kinetic parameters. These plots were prepared to aid readers in visualizing the discussion of Izumo1:Juno behavior across biologically relevant force regimes (**p.5-6**).

To address this, we made the following revisions:

1. We changed "compared" to "calculated" in the main text (**p.5**) to improve clarity.
2. In line with our response to Reviewer #1 comment 1, we have added the kinetic parameters presented in table S1 to the plots showing the calculated-force dependent off rates rather than simply referring to them in the legend.

10. "*Fig. S19G is totally blank. Is this on purpose? If so, I don't see the point in showing a blank plot.*"

Fig. S19G appears blank due to the absence of bond-clamping events passing the filtering and selection criteria. This serves as a negative control for the force clamp AFM-SMFS measurements. In contrast, representatives of the control experiments for the lower force regimes show 1-3 clamping events (**fig. S19C-D**).

To clarify this, we have made the following revisions:

1. We have added additional clarification in the **fig. S19** caption and in the figure panel itself (**SI. p51**).

11. “*What is the difference between Fig. 5C and Figs. S23 F-H? Are they different datasets? It is not clear from the text. Why not merge the different datasets to increase the statistical power?*”

The force clamp AFM-SMFS datasets shown in **Fig. 5C** and **fig. S23F-G** represent independently performed biological replicates (n = 3). While merging datasets could increase statistical power, we opted to keep them separate due to the sensitivity of the force clamp setup. Additionally, in keeping them separate, we are able to ensure that the variability across different biological replicates is accurately represented.

Fig. S23 H is a representative example of the 3 biological force clamp AFM-SMFS replicates performed at a higher force range for Izumo1:JunoH177Q. To ensure sufficient sampling within each bin for statistical testing, we needed to evaluate bond lifetimes in these two force-clamp regimes separately. Due to the sensitivity of the experimental set up, we did not feel comfortable merging the lower force (10-80 pN) and the higher force (80-160 pN) force clamp AFM-SMFS datasets as they represent separate experiments (different days, different surface preparations, different cantilevers, etc…).

To clarify these points, we have made the following revisions:

1. We have added a comment explicitly stating our use of biological replicates across our force clamp AFM-SMFS experiments in the main text (**p.11**).
2. We now explicitly state in the main text that the low force (10-80 pN) and the high force (80-160 pN) force regimes were measured separately to ensure sufficient sampling (**p.11**).

12. “*The pdb codes of the proteins and complexes used in the simulations should be provided.*”

We have added the PBD codes (5F4E, 5JKC, 5JKD, 5JKE) next to the relevant references in the description of the molecular dynamics set up (SI. p16)

13. “*SI line 298 “where l is (ln) loading rate.” Is “l” really “(ln) loading rate”?*”

We thank the reviewer for the careful reading of our SI. "*l*" refers to the loading rate itself, not its natural logarithm. The notation in (**SI p.11**) was corrected and we appreciate the attention to detail of the reviewer.

Reviewer #2 (Remarks to the Author):

“*I co-reviewed this manuscript with one of the reviewers who provided the listed reports. This is part of the Nature Communications initiative to facilitate training in peer review and to provide appropriate recognition for Early Career Researchers who co-review manuscripts.*”

We thank the reviewer for their feedback and contribution to improving this manuscript.

Reviewer #3 (Remarks to the Author):

“*This is a very interesting study of the interactions between the two key proteins involved in the process of fertilization, namely Izumo1 (from the sperm cell) and Juno (expressed on the egg cell surface.) The work is performed primarily at the single-molecule level using a powerful AFM-based single-molecule force*

spectroscopy, which brings the two proteins together and then directly measures the force necessary to separate them (detachment or rupture force). SMFS measurements are accompanied by all-atom steered molecular dynamics simulations, essentially modeling the AFM experiments and allowing the structural insights under force, at the atomic level. In addition, an infertility postulated mutation is engineered in Juno and its effects on the mechanostablity of the Izumo1-Juno complex are probed by experimental SMFS and computationally by SMD and Monte Carlo simulations. The main conclusion of this work includes identifying a new mechanism supporting the enhancement of the mechanical stability of the Izumo1-Juno interactions through so-called catch-bonds, which are bonds that counter-intuitively are stronger under force or are established under force. Another important conclusion is that the infertility-causing H177Q mutation in Juno adversely affects these catch bonds and overall weakens Izumo1-Juno interactions. The experimental and computational procedures are executed expertly, however, their description, particularly in the Supporting Information reveals some issues and inconsistencies that need to be improved/corrected."

We thank the reviewer for their positive comments and overall evaluation of the manuscript.

"*Below I list my suggestions and concerns:*
Main text:"

1. "*Please explain briefly the fingerprinting role of ddFLN4 in the main text and check the current description in the SI. Not everyone knows/remembers that two force peaks are expected for each ddFLN4 module, producing (mainly) a four-peak SMFS fingerprint.*"

We thank the reviewer for their suggestion to clarify the role of ddFLN4 as a single-molecule fingerprint. Each ddFLN4 domain unfolds via two distinct peaks at ~50 pN, corresponding to a contour length increase of ~32 nm. In our setup, ddFLN4 domains downstream of both Izumo1 and Juno result in a four-peak unfolding pattern with a total extension of ~64 nm, allowing precise identification of single-molecule events.

In response to this comment, we have made the following revisions:

1. We have added a brief explanation of the ddFLN4 fingerprint domain unfolding behavior to the main text (**p.3-4**).
2. We have reviewed and refined the SI description of the use of ddFLN4 fingerprint domains (**SI p.9**) to ensure consistency with our updated main text explanation.

2. "*P. 5 For both DHS and BE fitting of P0, P1, and P2 rupture events, we observed a several fold decrease in Δx"*
Δx is not defined in the main text up to this point. One can guess it is the distance to the unbinding transition state, but it should be clearly defined as such in the text. Also, in the SI, Δx has a double dagger, which is not used in the main text."

We appreciate the reviewer's suggestion. We made the following revisions:

1. We have a provided a definition of Δx in the main text upon its first mention (**p.5**)
2. We have replaced all instances of $\Delta x^{\ddagger}$ with Δx in the SI and updated eq. 6, 7, and 8 (**SI p.11**).

3. "*p. 6 "Across the constant speed AFM-SMFS Izumo1:Juno datasets, we also identified the presence of several intermediate Izumo1:Juno folded states. To further explore these observations and their*

relationship to P0, P1, and P2, we subdivided the P0, P1, and P2 force vs. extension curves based on the presence of these intermediates (Fig. 1D)".

Please explain how these intermediates manifest themselves and are identified in the force-extension curves."

These intermediate Izumo1:Juno folded states manifest in force vs. extension curves as additional sawtooth-like peak with short contour length increments that are distinct from the characteristic ddFLN4 fingerprint unfolding patterns (Fig. 1D). Figure S4 shows the forces at which these intermediates occur and their associated contour length increments.

To summarize our response to this comment we have made the following revisions:

1. We have added a description of how these intermediate Izumo1:Juno folded states present in the force *vs*. extension curves to the main text (**p.6**).

"*Supporting Information:*"

1. "p. 7, "In the final modification step, the ybbR-tags located at the C-terminus of the target proteins…" What is meant by the target protein here? The constructs and sequences involving ybbR tags shown on pp. 4 and 5 indicate the N-terminal location of ybbR not C-terminal."

On the AFM-handle, the ybbR-tag is indeed located at the N-terminus. However, following SpyTag/SpyCatcher isopeptide bond formation, the ybbR-tag/surface conjugation point is located at the C-terminus relative to the protein of interest (*i.e*. Izumo1/Juno). So with the SpyCatcher system, we have two proteins joined by an isopeptide bond that are oriented as follows: Surface-(N-term protein 1)-(C-term protein 1)-isopeptide-(C-term protein 2)-(N-term protein 2). This setup ensures that the native orientation of Izumo1/Juno on the cell membranes is preserved and maintains an orientation of SpyCatcher that prevents unfolding during constant speed AFM-SMFS experiments.

To summarize our response to this comment we have made the following revisions:

1. We have clarified the SI statement in question as the "the proteins of interest (*i.e*. Izumo1/Juno with covalently attached ybbR-tags present on AFM-handles)" to improve clarity regarding our experimental setup (**SI p.7**) .

2. Supp Info, p.8 "Boltzman", please correct spelling.

We have corrected "Boltzman" to "Boltzmann" in the SI (**SI p.8**) and further reviewed the supplemental section for possible errors of this nature.

3. "*The authors choose the Freely-Rotating-Chain (FRC) model for their molecular system to analyze force-extension data and to determine the contour length of their constructs, from the F-x data.*

Please provide some rationale for choosing this model over other approaches. Can the segment elasticity (bond-angle bending) be neglected at the forces explored in this work?

On page 8, the authors present various approximations to the FRC model to calculate the contour length L, following reference 52. Please review this section carefully as these equations are not copied/adapted correctly from reference 52. Please introduce the necessary definitions for the variable used, and check that they are consistently used.
Why would one like to calculate the bond length "b" using the Kuhn length "a" (eq. 2)? Also, "l" is the persistence length and not "p" in those equations."

This comment led us to re-examine the equations of the FRC model and we did indeed find a typographical error. We are confident the equations presented in the current version of the supplementary information are correct. In fact, reference 52 also contains a typographical error in the FRC equations that to our knowledge was never corrected (In ref. 52, terms "4" and "c" are in the denominator instead of the numerator in their respective expressions). We had personal correspondence with the authors some years ago about this typo. The corrected equations are given as follows:

$$\frac{x}{L} = \begin{cases} \frac{Fa}{3k_BT} & \text{for } \frac{Fb}{k_BT} < \frac{b}{p} \\ 1 - \left(\frac{4Fp}{k_BT}\right)^{-\frac{1}{2}} & \text{for } \frac{b}{p} < \frac{Fb}{k_BT} < \frac{p}{b} \\ 1 - \left(\frac{cFb}{k_BT}\right)^{-1} & \text{for } \frac{p}{b} < \frac{Fb}{k_BT} \end{cases}$$

Where *x* is the extension, *L* is the contour length, *F* is force, *a* is the Kuhn length and is given by Eq. (2), k_B is the Boltzmann constant, *T* is the temperature, *b* is the bond length, *p* is persistence length and is given by Eq. (3), and *c* is a constant of 2.

$$a = b\frac{1+\cos\gamma}{(1-\cos\gamma)\cos(\gamma/2)} \qquad \text{Eq. (2)}$$

Where Υ is the fixed angle connecting bonds in the FRC model.

$$p = b\frac{\cos(\gamma/2)}{|\ln(\cos\gamma)|} \qquad \text{Eq. (3)}$$

In terms of selecting a model for AFM data transformation and contour length mapping, our review article from 2020 (doi: 10.3389/fmolb.2020.00085) performed head-to-head comparisons of several non-linear spring models for polymer stretching that are commonly used in these types of experiments. For the force ranges that are relevant for this work, (~20-700 pN), the FRC with the selected parameters is quite an appropriate model. For completeness, we also tested the WLC model. However, we found it performed much worse than the FRC in terms of generating the correct contour length increments at Izumo1:Juno rupture following ELP extension and ddFLN4 unfolding. The ability of the FRC model to accurately reproduce the contour length increments for the fingerprint domains gives us confidence that it is suitable for our purposes. Nonetheless, at higher forces (>500 pN), we do expect some deviations between the stretching behavior of the ELP chains and the ideal FRC behavior which could limit our ability to pinpoint contour length changes in the high force range. The segment elasticity/bond-angle bending mentioned by the reviewer could in

theory also be considered, particularly at high forces. However, we see adequate fits and accurate detection of fingerprint domain sequence lengths using the model with the indicated parameters, and did not optimize this any further. This is an important point especially when pushing the detection limits of contour length mapping for identifying structural regions involved in, for example, intermediate infolding events with short contour length increments. The reviewer is also correct about the variable names, and we have now corrected and double checked the equations.

To address this comment, we have made the following revisions:

1. We added an explanation to the SI for our selection of the FRC model and the expected impact of bond-angle bending (SI, **p.8**).
2. Made the necessary corrections to Eq.1, 2, and 3 (SI, **p.8**).
3. We have reviewed the definitions of all variables for equations used in our study and we believe they are now all consistent.

4. "*p.8 "Under the first selection criteria, single molecule interactions between Izumo1 and Juno were identified as individual rupture events < 100 pN at contour lengths of ~135 nm This criterion was based on FRC-fitting of the expected contour length the polypeptide chains for this force range.*

The expected contour length of ~135 nm, needs to be carefully accounted for considering that the pulling handles have a complex composition involving unstructured ELPs and structured Spytag-Spycatcher modules that potentially may unfold in these measurements, in addition to the ddFLN4 domains. If this unfolding is prevented by the specific orientation of the SpyCatcher domains in the constructs, this should be explained and described in SI."

The reviewer is correct that the SpyCatcher domain in our constructs is oriented in such a way that it cannot unfold in the AFM-SMFS experiments. This was an important consideration in designing our experimental setup.

We revised the manuscript as follows:

1. We have updated the description of our AFM-handle constructs in the SI (**SI p.2**) to explicitly mention that the construct design prevents SpyCatcher unfolding due to the orientation.

Reviewer #4 (Remarks to the Author):

"*Izumo1:Juno mediate the adhesion between sperm and egg cells. This manuscript explores the role of mechanical forces in regulating the human Izumo1:Juno complex. While this study provides important insights into the mechanical underpinnings that regulate basis of human fertilization, there are numerous issues that need to be addressed before publication.*"

We thank the reviewer for taking the time to review the manuscript and their perspectives on how we can improve it.

"*Major Concerns*"

1. "*The manuscript is incredibly difficult to read and digest. Essential details required to interpret the experiments and simulations are missing from the manuscript. Key details and interpretations/discussion are buried a >60 page SI. Prior to publication, this manuscript will need to be completely overhauled.*"

We appreciate the reviewer's feedback and we understand that the SI is long. However, we present a detailed and thorough study on this molecular system that involves many advanced experimental and computational approaches, which takes time to explain in detail. In fact, other reviewers have stated that the manuscript is well structured and clear (see reviewer #1 comments), but we recognize the importance of ensuring accessibility for all readers. In response to this comment, we have streamlined key experimental, modeling, and simulation details throughout the main text and SI. We have taken particular care where other reviewers noted any unclear points. We hope these revisions address the reviewer's concerns while maintaining the level of detail necessary for scientific rigor.

2. "*While measuring catch bonds from AFM force clamp measurements are intuitive and rigorous, interpreting catch bonds from AFM single molecule force spectroscopy (SMFS) is fraught since interpretations are completely model dependent. Puzzlingly, while the authors invest so much effort in performing SMFS experiments (that are riddled with artifacts), they perform the force clamp measurements in an extremely superficial fashion (i.e. only Fig 3D). The force-clamp data is also described very cursorily. I would recommend that the authors perform force clamp measurements in a more rigorous and systematic manner and describe these measurements in far greater detail.*"

We appreciate the reviewer's concerns regarding the balance between force-clamp and constant speed atomic-force microscopy single-molecule force spectroscopy (AFM-SMFS) experiments in our study. However, we take issue with the blanket statement that the AFM measurements are "riddled with artifacts" based on no clear criticism or point of critique. Also, it is not clear what is meant when the reviewer writes that the force clamp measurements are performed in an "extremely superficial fashion". This is not true. It is possible that the reviewer did not understand the unfolding fingerprint approach that we use to validate single-molecule interactions (described below).

We agree that force-clamp AFM-SMFS measurements provide a rigorous means to detect catch bonds, but catch bonds can also be detected using constant speed AFM-SMFS in combination with kinetic modeling, as we have shown here and elsewhere. Since constant speed AFM-SMFS approaches are well-established and much easier to implement experimentally, they also provide valuable datasets for characterizing catch bonds. The combination of both force clamp and constant speed measurements is in our opinion a very rigorous approach, and we have undertaken both methods here to a very high standard.

Contrary to the impression of the reviewer that our force-clamp AFM-SMFS measurements were superficial, we report four distinct force-clamp AFM-SMFS experiments, each performed in biological triplicates (**Fig. 3D** and **5C**, **fig. S19** and **S23**), against six distinct constant speed AFM-SMFS experiments, also each in biological triplicate.

However, we recognise the emphasis placed on the constant speed AFM-SMFS approach in the main text. Here, we agree that constant speed AFM-SMFS and the corresponding theoretical landscape interpretations of catch bond behavior are less intuitive than force clamp AFM-SMFS bond lifetime measurements. We therefore felt that readers would benefit from additional clarification and a technical emphasis.

In response to this comment, we expanded the description of the force-clamp approach in the main text and sought to further clarify its role in this study (**p.11-12**). We thank the reviewer for their

perspective and believe these changes serve to emphasize the extensive efforts we have put into using both constant speed and force clamp AFM-SMFS to support our conclusions.

3. "*It is unclear how the authors decided from the SMFS experiments that Izumo1:Juno predominantly unbind along three distinct pathways. This is a critical point in this paper and is poorly justified. It appears that the authors determined multiple unbinding pathways solely using a Gaussian Mixture Model which is not convincing. At the very least, the authors need to provide orthogonal experimental evidence using a different experimental technique to justify their model.*"

We recognize the reviewer's concern regarding the justification for the three-pathway unbinding model. However, we would like to clarify that our interpretation is not based solely on Gaussian Mixture Model (GMM) fitting, but rather on a rigorous, multi-faceted approach the involves agreement between: 1) orthogonal modes of AFM-SMFS (constant speed and force clamp); 2) Monte Carlo force spectroscopy simulations and kinetic modeling; and 3) all-atom Steered Molecular Dynamics simulations. GMM fitting is simply used as a tool for robust and reproducible pathway classification across multiple biological replicates and Izumo1:Juno variants.

Given the convergence of experimental and computational evidence, we believe our three-pathway model is strongly justified. To further clarify this point for future readers, we sought to emphasize this multi-step validation process during our revision of the manuscript, in particular in the conclusion of our Monte Carlo force spectroscopy simulation section (**p.12**) **.** We hope our response and these clarifications address the reviewers' concern regarding the use of the GMM for unbinding pathway classification.

4. "It is also unclear how the authors determined from their AFM data that the P0 pathway involves no intermediate states while the P1 and P2 pathways involve intermediate states. It appears that the only justification for this were the small unbinding peaks in the SMFS force curves which is a wholly insufficient justification since it could be an artifact of surface functionalization or due to multiple unbinding events. Several additional controls and orthogonal experiments are needed to substantiate this claim."

We appreciate the reviewer's concern regarding the interpretation of the P0, P1, and P2 pathways and their association with intermediate states. However, as discussed throughout the manuscript, our conclusions are supported by multiple lines of evidence. Regarding the specific points raised, the reproducible loading-rate dependence of these events and contour length differences observed during mutational analysis contradict the randomness expected from non-specific adhesion or multiple-binding artifacts. Additionally, the conformational changes observed in wild-type Izumo1:Juno all-atom SMD simulations (13.4–14.1 nm) closely match those in AFM-SMFS experiments (14.8–15.1 nm at 3200 nm/s).

We used various experimental design strategies (*e.g.* ELP linkers, unfolding fingerprint domains (i.e. ddFLN4), and low-density site-specific immobilisation) to rule out artifacts from surface functionalization or multiple-binding events.

Nonetheless, to improve clarity for future readers, we have further elaborated on this rationale, emphasised the use of controls in describing how these intermediates manifest on force *vs*. extension curves, summarized our conclusions from the mutational analysis, and highlighted the convergence of the experimental and all-atom SMD data in the revised manuscript (main text: **p.6, 9,10. SI: p.25**).

5. “*In the Izumo1:Juno rupture force vs. loading rate plots, the P1 and P2 experimental data (purple and cyan in Fig 1F) do not fit to the DHS model. How do the authors rationalize these poor fits?*”

Due to the nature of how the Dudko-Hummer-Szabo (DHS) model is fitted, it does not always closely follow the median rupture force vs. median loading rate points. This is because the DHS model fits the full probability distribution of the pathway rupture forces rather than just the median rupture forces and median loading rates for each constant pulling speed. This effect is particularly evident in complex multi-state systems, where a complex passes through multiple states and pathways prior to unbinding. Furthermore, the DHS is as stated by the reviewer, simply a model. It may be the case that our system does not perfectly meet the underlying assumptions of the model. This is the case for all theoretical models used in this field. Each has its own limitations and domains of applicability. To address this concern we have further refined the SI description of how the DHS model was fit, and we explicitly state its relationship to the median loading-rate and median rupture forces (**SI p10-11**).

6. “*The authors should provide the probability of single and multiple unbinding events. These probabilities are needed to determine if the unbinding event rate follows a Poisson distribution in order to determine the likelihood that they are indeed single molecule unbinding events.*”

We appreciate the reviewer’s suggestion to assess single-versus multiple-molecule unbinding events using a Poisson distribution. However, a formal Poisson analysis would require manual reanalysis of tens of thousands of curves and be statistically uninformative given the low binding frequency in our AFM-SMFS datasets. This low frequency results from our stringent, low-density, site-specific surface functionalization strategy, which minimizes nonspecific interactions and ensures a low Izumo1:Juno binding probability (~1-5% of force *vs*. extension curves). As a result, multiple simultaneous Izumo1:Juno interactions are exceedingly rare. When binding occurs, single-molecule events are reliably identified by the characteristic contour length of the defined ELP linkers and the unfolding signature of the ddFLN4 fingerprint domain. These same features enable robust exclusion of multiple binding events, which would yield aberrant force profiles. If we form multiple tethers between Izumo1:Juno proteins during an AFM tip-sample surface interaction, the ddFLN4 unfolding fingerprint signature will include complex multi-protein unfolding events. These are then excluded in the curve filtering and data post-processing which avoids analyzing curves with multiple unbinding events. These techniques are very much established in our group and considered standard in the field.

To address the reviewer’s concern regarding the prevalence of multiple interaction events, we now explicitly highlight the absence of multi-event traces in our AFM-SMFS datasets, explicitly state the percentage of force *vs*. extension traces showing evidence of Izumo1:Juno interactions. We also clarify how our experimental design avoids analyzing multi-tether events (**SI p.8-9, 12**).

7. “Are the ‘histograms’ in Figure 1D, conventional histograms or are they kernel density estimators? Some of these histograms only have 22 events (for instance P1a) and seem remarkably normally distributed for such a low event rate. Also why do the authors refer to these as “force vs. contour length histograms”? These just seem like 1D histograms of the contour length (and not 2D force vs. contour length histograms). The authors need to be more precise in the language they use throughout the manuscript.”

We thank the reviewer for the attention to detail. The reviewer is correct that our phrase "force *vs*. contour length histograms" was not accurate. These are indeed 1D histograms of contour length. We have corrected this in the main text (**p.4)**. To construct these histograms, we transform the data

of a single force vs. extension trace into a contour length histogram, and then align multiple contour length histograms for many data traces using cross-correlation analysis before combining (summing) them into a single plot. This type of data analysis method has been presented in detail in prior review articles: (DOIs: 10.1529/biophysj.108.129999 ; and 10.3389/fmolb.2020.00085).

8. "*Several critical controls are lacking. For instance, how did the authors rule-out that P1 and P2 are separate pathways and do not merely arise from unbinding of multiple Izumo1:Juno molecules?*"

As outlined in the main text and SI, we have taken extensive experimental and analytical steps to rule out multiple-molecule artifacts:

- Single-molecule verification: We use ddFLN4 fingerprint domains as internal controls to confirm that each force *vs*. extension trace corresponds to a single molecular interaction. Traces deviating from the expected pattern, such as those indicative of multiple unbinding events, are excluded from the analysis.

- Low binding probability: Experiments were conducted on surfaces with a very low density of molecules. The resulting low binding frequency (~1–5%) means multiple simultaneous Izumo1:Juno interactions are exceedingly rare. If they occur, they are filtered out by the fingerprint domain method (bullet point above).

- Loading rate dependency: If P1 and P2 resulted from multiple-Izumo1:Juno complexes unbinding, their relative frequencies would be random across different pulling speeds. Instead, we observe a clear loading rate dependence, consistent with distinct unbinding pathways.

- Mutant behavior: Specific mutants (*e.g.* Izumo1:JunoH177Q) alter the relative frequency and mechanostability of P1 and P2 events, further supporting their origin as distinct, force-dependent, unbinding pathways.

- Simulation validation: Monte Carlo simulations of constant-speed and force-clamp AFM-SMFS accurately reproduce the observed P1 and P2 behavior. Simpler one- or two-pathway models fail to account for experimentally observed dataset.

- All-atom SMD support: Independent all-atom SMD simulations also provide a structurally plausible mechanism that could give rise to the three distinct unbinding pathways in Izumo1:Juno, reinforcing the experimental observations.

In summary, the combined evidence from internal controls, low binding frequency, mutational analysis, Monte Carlo kinetic modeling, and all-atom SMD simulations strongly supports that P1 and P2 arise from distinct unbinding pathways—not from multiple-molecule events. Nonetheless, in an effort to address this reviewer's concern and improve clarity for future readers we have revised the manuscript to emphasise this point further (**main text p. 3–4. SI p.8-9, 12**).

9. "*The SMD simulations performed with the JunoH177Q mutation are intriguing. I believe that further molecular details can be obtained from these simulations by performing additional analysis. For instance, the authors only analyze the contact area between Izumo and Juno (which I assume is the Solvent Accessible Surface Area, although this is never specified). However, I think they should also monitor the*

formation of hydrogen bonds and other electrostatic interactions that are abolished by the JunoH177Q mutation. For instance, in order to conclude that the increased mechanostability of JunoH177E is charge related, the authors should monitor salt-bridge formation across the simulation frames"

We would like to clarify that the "contact area" mentioned in our analysis refers to the "buried surface area" between Izumo1 and Juno, which is by definition no longer solvent accessible upon complex formation. The use of "contact area" over "buried surface area" is well established in the molecular dynamics field, particularly when emphasizing dynamic, per-residue contributions across simulation frames.

Regarding the reviewer's suggestion to analyze non-covalent interactions such as hydrogen bonds and salt bridges: this analysis was performed and is presented in the main text and SI. **Fig. 4** provides direct visualizations of the key terminal interactions between Izumo1N59&R56-JunoH177 and Izumo1K48-JunoH177. In addition, **fig. S6** (two-state unbinding) and **S8** (three-state unbinding) provide detailed time traces for all the non-covalent interactions between Izumo1 and Juno—including hydrogen bonds, salt bridges, and hydrophobic contacts. The corresponding loss of the key interactions in Izumo1:JunoH177Q is shown in **Fig. 4,** and a detailed time trace of all the non-covalent interactions for three-state unbinding is presented in **fig. S21**. However, we chose not to present the simulated interaction map for all variants/simulations studied. We saw that, due to the size and dynamic nature of the IZUMO–JUNO interface, identical interactions are not consistently maintained across all trajectories—particularly under the force-driven conditions of SMD. This variability makes aggregated counts less informative at our current sampling level.

Regarding the specific analysis of the Juno177E variant, two trajectories chosen of this system are shown below. They do not show clear differences as the E177 doesn't create interactions that pass previously established threshold. If there are salt-bridges formed they might be more transient in nature, or the key interactions are not with IZUMO but with neighbouring residues creating more dense network. Due tot he current length of the manuscript, at this point we do not feel it is necessary to include this as additional supplementary information.

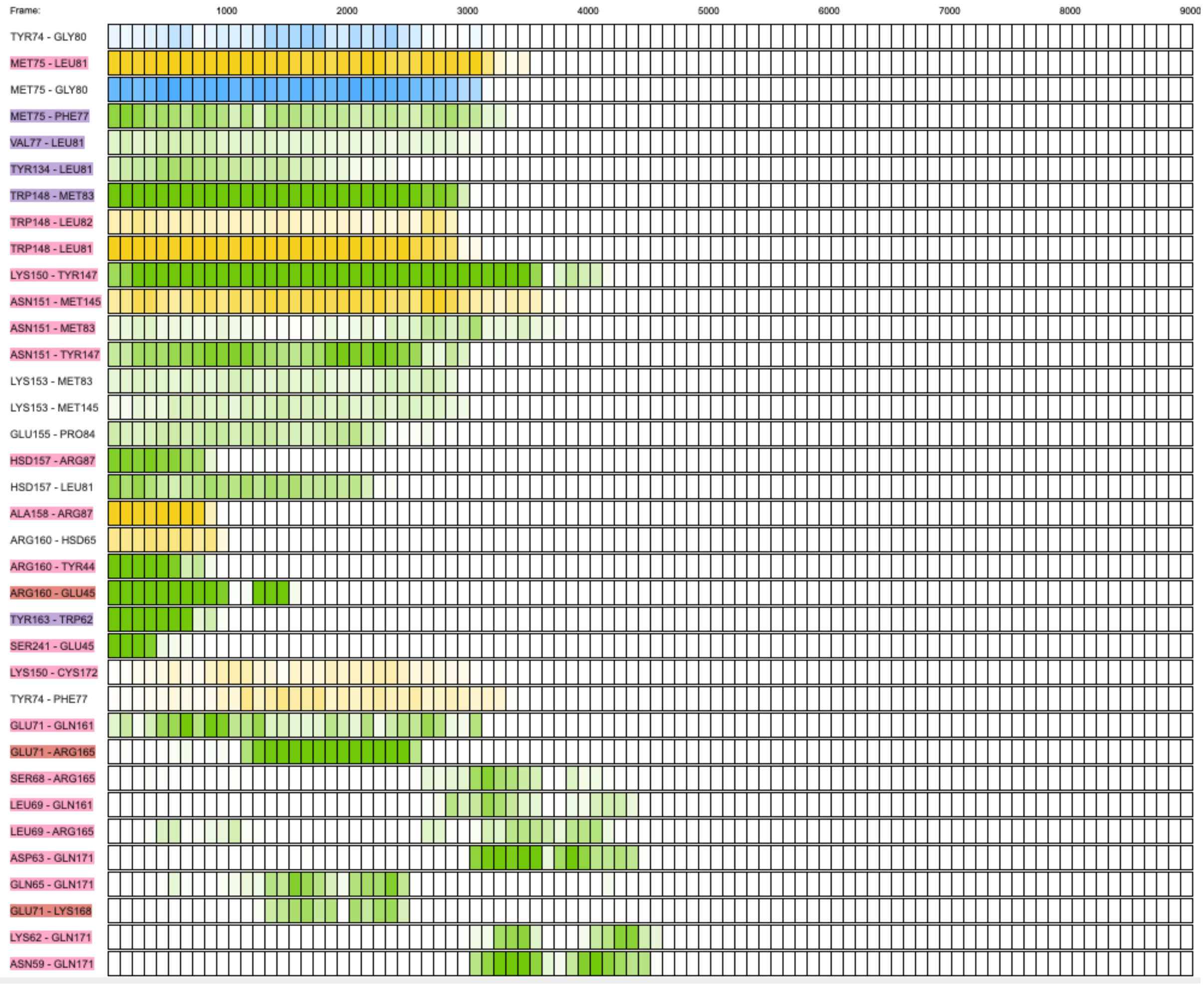

Exemplary time series of the non-covalent interactions as observed in the three-state unbinding pathway of the Izumo1:JunoH177E mutant complex in SMD simulations.

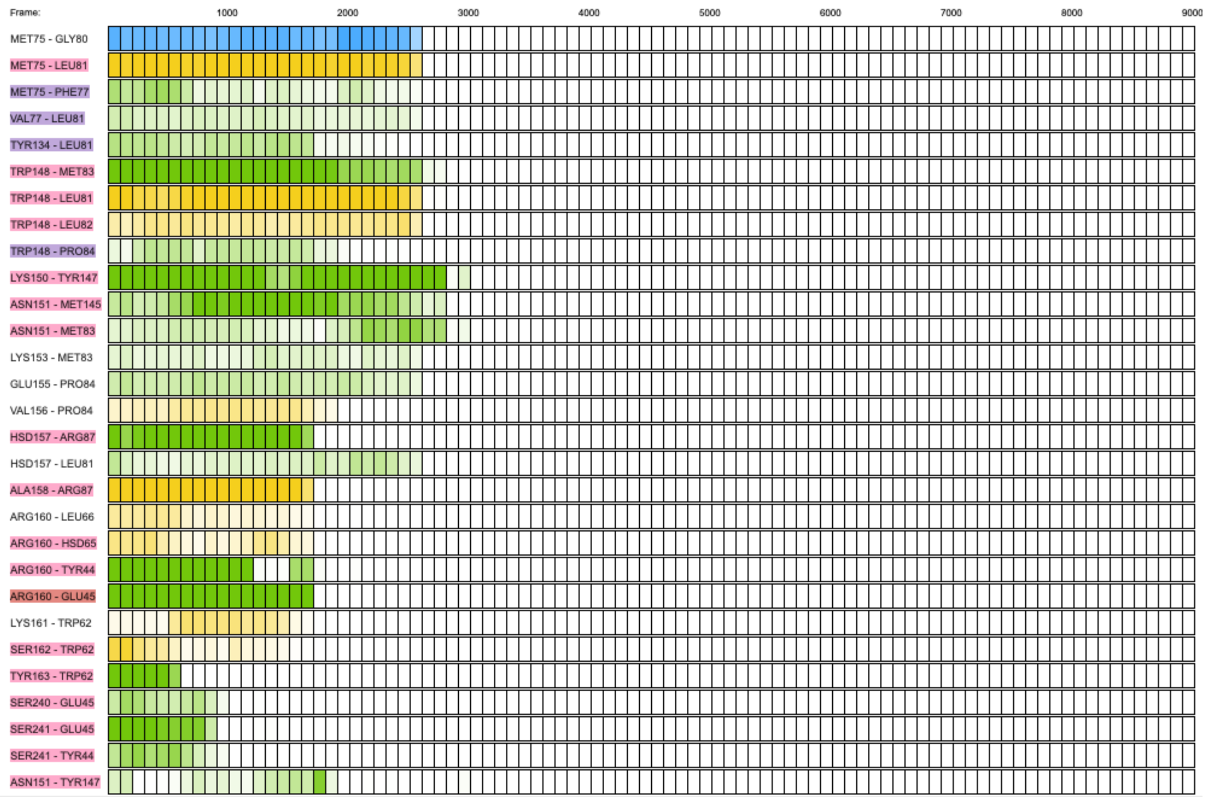

Figure. Second exemplary time series of the non-covalent interactions as observed in the two-state unbinding pathway of the Izumo1:JunoH177E mutant complex in SMD simulations in S-2.

"*Minor Concern*"

1. "*The statement on page 3 that "presence of C-terminal SpyTags were used to confirm the successful expression and purification of Izumo1 and Juno" is vague. The authors need to explicitly state what they mean by this statement.*"

The C-terminal SpyTag on Izumo1 and Juno were used as a tool for attaching the AFM-handles and fluorescent proteins for AFM-SMFS and binding affinity characterisation, respectively. However, SpyTags have also become well established as a genetically encoded tag for the high-fidelity identification of proteins on SDS-PAGE gels. We incubated purified SpyTagged protein samples with SpyCatcher containing proteins for 1 h. Where the SpyTag/SpyCatcher isopeptide bond forms, a molecular weight shift is observed for SpyTagged proteins proportional to the addition of the SpyCatcher protein on an SDS-PAGE gel.

To address this concern, we have added additional clarification to our original statement as requested by the reviewer (**p.3**).
